# Scope of Practice Laws Affecting Team‐Based Heart Disease Care in Rural America: Policy Challenges and Opportunities

**DOI:** 10.1161/JAHA.126.049493

**Published:** 2026-09-24

**Authors:** Jeff Lane, Signe Burchim, Amanda Li, Janessa M. Graves, Chris Longenecker, Sarah Masyuko, Jennifer Bacci, Sarah Gimbel, Allison Webel, Margaret Hammersla, Cassandra Leonard, Evan Foster Shalen, Bridget O'Connell, Rose Thoroughman, Joseph P. Ornato, Bianca K. Frogner

**Affiliations:** ^1^ Department of Global Health University of Washington Seattle WA USA; ^2^ Department of Family Medicine University of Washington Seattle WA USA; ^3^ School of Pharmacy, University of Washington Seattle WA USA; ^4^ School of Nursing, University of Washington Seattle WA USA; ^5^ Mark & Robyn Jones College of Nursing, Montana State University Bozeman MT USA; ^6^ Idaho Office of Underserved and Rural Medical Research, Idaho WWAMI Medical Education Program University of Idaho Moscow ID USA; ^7^ Division of Cardiovascular Medicine Knight Cardiovascular Institute, Oregon Health and Science University Portland OR USA; ^8^ Palo Alto Veterans Institute for Research (PAVIR) Palo Alto CA USA; ^9^ Heart Institute, Cincinnati Children's Hospital Medical Center Cincinnati OH USA; ^10^ Department of Emergency Medicine Virginia Commonwealth University Richmond VA USA

**Keywords:** cardiovascular disease, health policy, rural health, scope of practice, team‐based care, Ethics and Policy, Health Services

## Abstract

**Background:**

Heart disease is the leading cause of death in the United States with a disproportionate impact in rural America, and health worker shortages contribute to these disparities. Team‐based heart disease care models can help mitigate health worker shortages, but lack of role clarity and restrictive scope of practice laws can be a barrier to their implementation.

**Methods:**

To better understand the intersection of licensed scope of practice laws with rural heart disease care, we performed a legal epidemiology analysis to identify and describe licensed scope of practice laws for 9 health worker cadres in 13 state and federal jurisdictions for 8 heart disease services.

**Results:**

We found substantial ambiguity, variation, and some important gaps, which may undermine the predictability of regulatory and reimbursement environments and hinder implementation of innovative team‐based care models.

**Conclusions:**

Three policy opportunities to address the identified gaps include: (1) linking licensed scope of practice definitions to the scope of accredited training curricula, (2) shifting away from collaborative practice agreements to define licensed scope of practice, and (3) prohibiting insurance plans from conditioning reimbursement based solely on provider cadre.

**Nonstandard Abbreviations and Acronyms**
CHW: community health worker
LPN: licensed practical nurse
MA: medical assistant
NP: nurse practitioner
PA: physician assistant

**Research Perspective**

**What Is New?**

- This legal epidemiology analysis reveals substantial ambiguity, variation, and gaps in licensed scope of practice laws for 9 health worker cadres across 13 jurisdictions, contributing to regulatory uncertainty that may undermine team‐based heart disease care in rural areas.

**What Question Should be Addressed Next?**

- Future research should explore how health workers navigate scope of practice laws and how the regulatory environment impacts the implementation of team‐based heart disease care in rural communities.

Heart disease (eg, heart attacks, heart failure, coronary artery disease) is the leading cause of death in the United States, accounting for more than 900 000 deaths in 2023. Heart disease rates vary substantially by geography, race, and sex.^1^ Heart disease death rates are 21% higher in rural areas compared with urban areas, even after adjusting for age differences.^2^ People identifying as non‐Hispanic Black, Native Hawaiian or Other Pacific Islander, or White were 263%, 213%, and 210%, respectively, more likely to die from heart disease than those identifying as Asian (despite heart disease being the second leading cause of death for people identifying as non‐Hispanic Asian).^3^ Though it is the leading cause of death in both men and women, men are 60% more likely to die from heart disease than women.^4^, ^5^ When combining race, sex, and geography, rural Black men have the highest rates of age‐adjusted heart disease mortality.^6^, ^7^

## Rural Health Workforce and Heart Disease Care

To deliver a comprehensive heart disease care system, rural communities need to recruit and retain a diverse range of health workers that can provide preventive care, emergency care, and chronic disease management. Effective distribution of the health workforce across rural regions is also critical because of the time sensitive nature of cardiac emergencies and the need for ongoing, often lifelong, chronic disease management. Preventive care drives improved heart disease outcomes, but requires an adequate workforce trained in identifying and addressing a wide range of biological, behavioral, and environmental risk factors, including high blood pressure and high cholesterol, diabetes, obesity, poor nutrition, lack of physical activity, tobacco use, alcohol use, genetics,^8^ and air pollution.^9^

Health workforce shortages present a major barrier to implementing comprehensive heart disease prevention and treatment programs in rural areas. The Health Resources and Services Administration's National Center for Health Workforce Analysis estimates that the supply of cardiologists in rural areas in 2025 was 70% below need and is projected to worsen.^10^ In addition, the United States faces shortages of many other health workforce cadres essential for heart disease care, including primary care physicians, registered nurses (RNs), licensed practical nurses (LPNs), and pharmacists.^10^, ^11^ By 2027, rural areas are projected to have a 27% shortage of RNs versus 7% in urban areas.^12^ While the supply of emergency medical services (EMS) providers is projected to grow nationally relative to increasing demand,^10^ rural communities often rely on volunteer emergency medical technicians (EMTs),^13^ and EMT volunteerism is declining,^14^ which may compromise response times to cardiac emergencies. In contrast, there has been a marked increase in the supply of rural nurse practitioners (NPs) and physician assistants (PAs) over the past 20 years,^15^ highlighting the growing critical role these cadres have in delivering rural heart disease care.

## Scope of Practice

Team‐based care models that use multiple cadres of health workers are key to delivering comprehensive heart disease care amid health workforce shortages and have been successfully implemented in the United States and globally.^16^, ^17^, ^18^ The US Community Preventive Services Task Force recommends team‐based care as an effective strategy to improve blood pressure control.^19^

Optimizing team‐based care models requires team members to have clarity about the permitted scopes and roles of cross‐disciplinary members.^20^, ^21^, ^22^ A health worker's effective scope of practice is influenced by various governmental and non‐governmental actors. Health worker licensure statutes adopted by state legislatures and regulations adopted by state professional boards define a health worker's licensed scope of practice. Federal law defines scope of practice for prescribing controlled substances. Federal laws also determine the scope of practice for health workers serving in the federal Veterans Health Administration and Indian Health Service, preempting state health worker scope of practice laws. Health care facility and organizational policies can further restrict scope of practice by cadre. Federal and state health insurance reimbursement rules and private health insurance plans can also condition payment on services being provided by certain cadres.^23^ Some state licensing laws condition scope of practice by requiring health workers to have delegated authority agreements with a physician (eg, a collaborative practice agreement between a physician and a pharmacist), which may be harder for providers in rural areas to establish compared with their urban counterparts because of administrative and financial barriers and fewer physicians practicing in rural communities.^24^ All of these determinants can influence a health worker's effective scope of practice, and often the permitted scopes within these determinants do not align, creating confusion and administrative barriers to team‐based care and preventing health workers from practicing at the top of their license and to the full extent of their education and training.

## Objective

The purpose of this analysis was to characterize and compare the differences and similarities in the licensed scope of practice for health worker cadres in a purposively selected sample of jurisdictions, including state and federal jurisdictions, regarding a diverse set of heart disease care services. We present the results of this analysis and then discuss 3 policy opportunities that may help maximize the contributions of health workers from all cadres, improve administrative efficiency, and scale innovative team‐based care models for heart disease care nationwide.

## METHODS

The authors declare that all supporting data, including the full codebook, definitions, and coding guidelines, are available as online supplementary files. There are many definitions of the health worker scope of practice,^25^ but we use the term “licensed scope of practice” to refer to the services a health worker is authorized to provide under state and/or federal law. We applied legal epidemiology and policy coding methods, as described by Burris et al^26^ and Lane et al,^27^ to characterize the licensed scope of practice for 9 cadres in 13 jurisdictions relating to 8 heart disease care services. We used state legislature websites and the Westlaw legal database to identify laws, including statutes and regulations, in effect as of July 1, 2024. We selected the jurisdictions, cadres, and services based on a review of the innovative models being studied within the Rural PRO‐CARE Network. Rural PRO‐CARE is a research network funded by the American Heart Association aimed at identifying innovative care models to reduce heart disease health disparities by delivering heart disease screening, prevention, and/or treatment services to rural underserved communities. The cadres included: NP, PA, pharmacist, RN, LPN, medical assistant (MA), EMT, paramedic, and community health worker (CHW). The jurisdictions were selected based on the locations where Rural PRO‐CARE Network members are studying innovation models and included 11 states (Alaska, Arizona, California, Colorado, Idaho, Michigan, Montana, North Carolina, Oregon, Virginia, and Washington) and 2 federal health care delivery systems (Veterans Health Administration and Indian Health Service). A multidisciplinary team of clinicians and researchers participating in the network identified eight services that are essential to delivering comprehensive heart disease care: prescribing antihypertensive medicines; prescribing heart failure medicines; monitoring of International Normalized Ratio and modifying anticoagulant dosing; administering thrombolytics for ST‐segment–elevation myocardial infarction (STEMI); conducting point of care ultrasound screening for heart disease; conducting an ankle brachial index test; conducting an ECG; and conducting a blood pressure test. We subsequently grouped these services into four service categories: (1) prescribing heart disease medicines; (2) administering thrombolytics for STEMI; (3) conducting moderately complex diagnostic tests; and (4) conducting simpler diagnostic tests (Table 1).

**Table 1:** Jurisdictions, Cadres, and Services Analyzed

Jurisdictions	Cadres	Service category	Services (*Definition*)
Alaska Arizona California Colorado Idaho Michigan Montana North Carolina Oregon Virginia Washington Veterans Health Administration Indian Health Service	Nurse practitioner Physician assistant Pharmacist Registered nurse Licensed practical nurse Medical assistant Emergency medical technician Paramedic Community health worker	Prescribing medicines	Prescribing antihypertensive medicines (*medication for the management of high blood pressure*)
			Prescribing heart failure medicines (*multiple medications that need to be closely monitored and titrated for the management of heart failure*)
			Monitoring of INR and modifying anticoagulant dosing (*medication for the management of blood clots*)
		Administering thrombolytics	Administering thrombolytics for STEMI (*emergency medication administered to patients experiencing STEMI*)
		Moderately complex diagnostic tests	Conducting point of care ultrasound screening for heart disease (*uses a handheld ultrasound device to check the structure and function of the heart*)
			Conducting an ankle brachial index test (*uses inflatable cuff to measure and compare pressure in the arteries in the upper arm and ankle to detect peripheral artery disease*)
			Conducting an ECG (*uses temporary electrodes to monitor the heart's electrical activity*)
		Simpler diagnostic tests	Conducting a blood pressure test (*uses inflatable cuff to measure pressure in the arteries in the upper arm*)

INR indicates international normalized ratio; and STEMI, ST‐segment‐elevation myocardial infarction.

We used Microsoft Excel to extract laws on licensed scope of practice for each combination of cadre and service in our sample jurisdictions, including the following: statute citation, narrative summary, regulation citation (if any), and narrative summary of regulation (if applicable). Applicable laws were identified using Westlaw and Google searches of state legislature websites and secondary web sources. Applicable laws were authenticated using Westlaw and extracted from Westlaw. Our initial coding scheme included the following codes: Yes, No, Maybe, and Unclear. Coders (JL, SB, and AL) met weekly throughout the coding process to iteratively refine the coding framework. After completing coding for six jurisdictions, we expanded the coding scheme to include Likely yes and Likely no, which captured instances where we categorically interpreted the scope of practice as permitting or not permitting the service even if it was not explicitly referenced in statute or regulation. We recoded the pilot jurisdictions using the updated framework, which we also applied to the remaining jurisdictions (Table 2).

**Table 2:** Scope of Practice Coding Framework

Code	Definition
Yes	Service explicitly listed as within scope of practice
Likely yes	Service is not explicitly listed as within scope of practice or prohibited, but the categorical description suggests this service is within the scope of practice
Maybe	Additional training, supervision, or agreement may allow a person of this cadre to provide this service
Unclear	Scope of practice unclear relating to this service
No	Service explicitly prohibited or clearly outside the scope of practice based on categorical description
Likely no	Service is not explicitly listed as being within scope of practice or prohibited, but the categorical description suggests this service is outside scope of practice

We coded each combination of cadre, service, and jurisdiction as one of the following: Yes, Likely yes, Maybe (conditional), Unclear, Likely no, or No. One research team member (AL or SB) conducted initial coding, which was then reviewed and verified by a different team member (JL or SB). JL and SB discussed all differences in coding until consensus was reached. The full codebook, including definitions and coding guidelines, is available in the Supplemental Publication Material as Tables S1–S7. We used Microsoft Excel to calculate descriptive statistics to compare coding results within and across cadres and jurisdictions. This study analyzed publicly available state laws and did not involve human subjects; therefore, Institutional Review Board review was not required.

### Jurisdiction Alignment Score

We developed and calculated a Jurisdiction Alignment Score for each cadre to measure the degree to which different jurisdictions defined the licensed scope of practice for the same cadre similarly. A high Jurisdiction Alignment Score for a cadre would indicate that the licensed scope of practice for that cadre were very similar between jurisdictions. In contrast, a low Jurisdiction Alignment Score would indicate that the licensed scope of practice for that cadre varied substantially between jurisdictions. These scores were calculated by first combining Yes/Likely yes and No/Likely no into the same code to create 4 code categories: Yes/Likely yes, No/Likely no, Maybe, and Unclear. We identified the highest number of aligned codes for each cadre and then divided that number by the total number of jurisdictions possible. We also noted the number of total unique codes for each cadre and service combination. For example, for NP authority to prescribe antihypertensive medicines, if we coded 8 of 13 jurisdictions as Yes/Likely yes, 4 as Unclear, and 1 as No/Likely no. The Jurisdiction Alignment Score for this service would be 8/13 (0.62) with 3 unique codes, represented as 0.62 (3). Each cadre had an average Jurisdiction Alignment Score representing the alignment between jurisdictions across all 8 services.

## RESULTS

Our analysis covered 13 jurisdictions, 9 cadres, and 8 services, generating a total of 936 domains for coding. Figure 1 summarizes the key results for the four coding categories and four service categories. The full results of our coding are included in Table S8 of the supplemental files.

**Figure 1:**
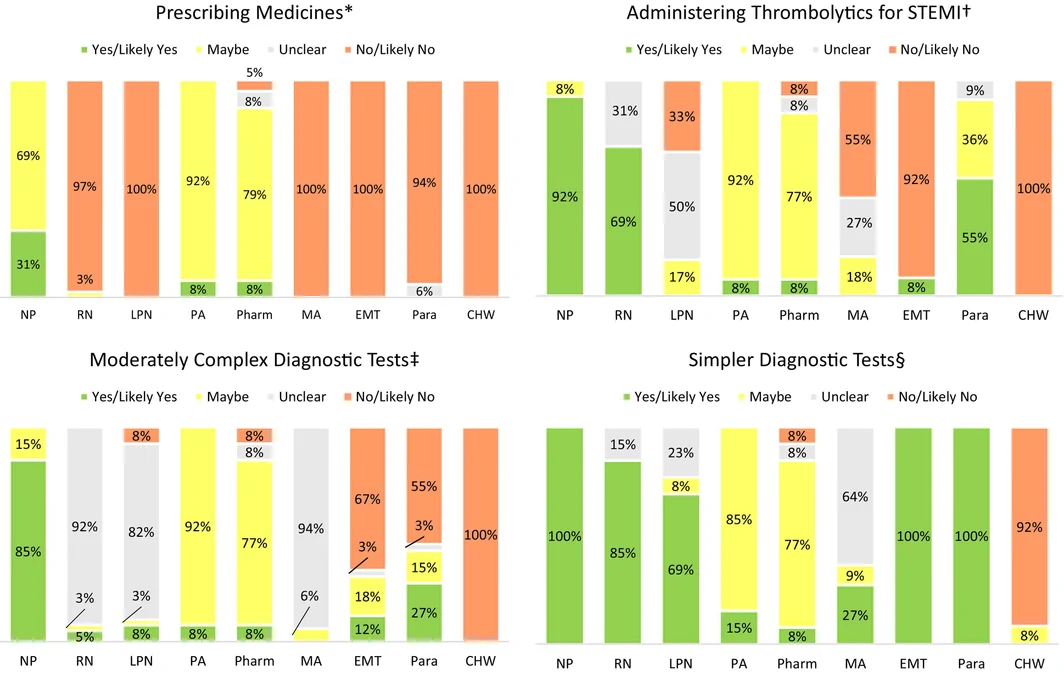
Summary results of scope of practice for heart disease services, effective July 1, 2024. CHW indicates community health worker; EMT, emergency medical technician; LPN, licensed practical nurse; MA, medical assistant; NP, nurse practitioner; PA, physician assistant; Para, paramedic; Pharm, pharmacist; and RN, registered nurse. *Includes the following services: prescribing antihypertensive medicines, prescribing heart failure medicines, and monitoring International Normalized Ratio (INR) and adjusting anticoagulant medicine dosing. ^†^Includes the following service: intravenous administration of thrombolytics for ST‐segment–elevation myocardial infarction (STEMI). ^‡^Includes the following service: conducting point of care ultrasound screening for heart disease, conducting an ankle brachial index test, and conducting an ECG. ^§^Includes the following service: conducting a blood pressure test.

### Characterizing Licensed Scope of Practice

#### Breadth and Variation in Licensed Scopes of Practice

NPs had the broadest licensed scope of practice, with 67% (*n*=70/104) of domains coded as Yes/Likely yes. With respect to prescribing authority, NPs had prescribing authority in all jurisdictions, though additional training or collaborative practice agreements were required in 9 of the 13 jurisdictions. RNs had narrower licensed scope of practice with 20% (n=21/104) of domains coded as Yes/Likely yes and LPNs had even narrower scope at 12% (n=12 /104) of domains coded as Yes/Likely yes (Figure 2). NPs and LPNs had the lowest average Jurisdiction Alignment Scores with jurisdictions having the same code only 82% of the time. PAs licensed scope of practice was conditional on a collaborative practice agreement in almost all jurisdictions (92%, n=12/13). Idaho was the only jurisdiction that included prescribing in the pharmacist's standard licensed scope of practice without a collaborative practice agreement. All EMS workers (EMTs and paramedics) had detailed but somewhat narrow scopes of practice (EMTs: 16% Yes/Likely yes, n=16/88; Paramedics: 30% Yes/Likely yes, n=26/88) as their work is primarily performed in prehospital transport settings. Some jurisdictions provided guidance for MAs for basic screening practices like conducting blood pressure tests, for which 3 jurisdictions were coded as Likely yes for MAs (27%). We did not identify laws defining licensed scopes of practice for CHWs in any of our sample jurisdictions, leaving a critical gap in the regulatory environment for an increasingly important cadre for delivering heart disease prevention and screening services.

**Figure 2:**
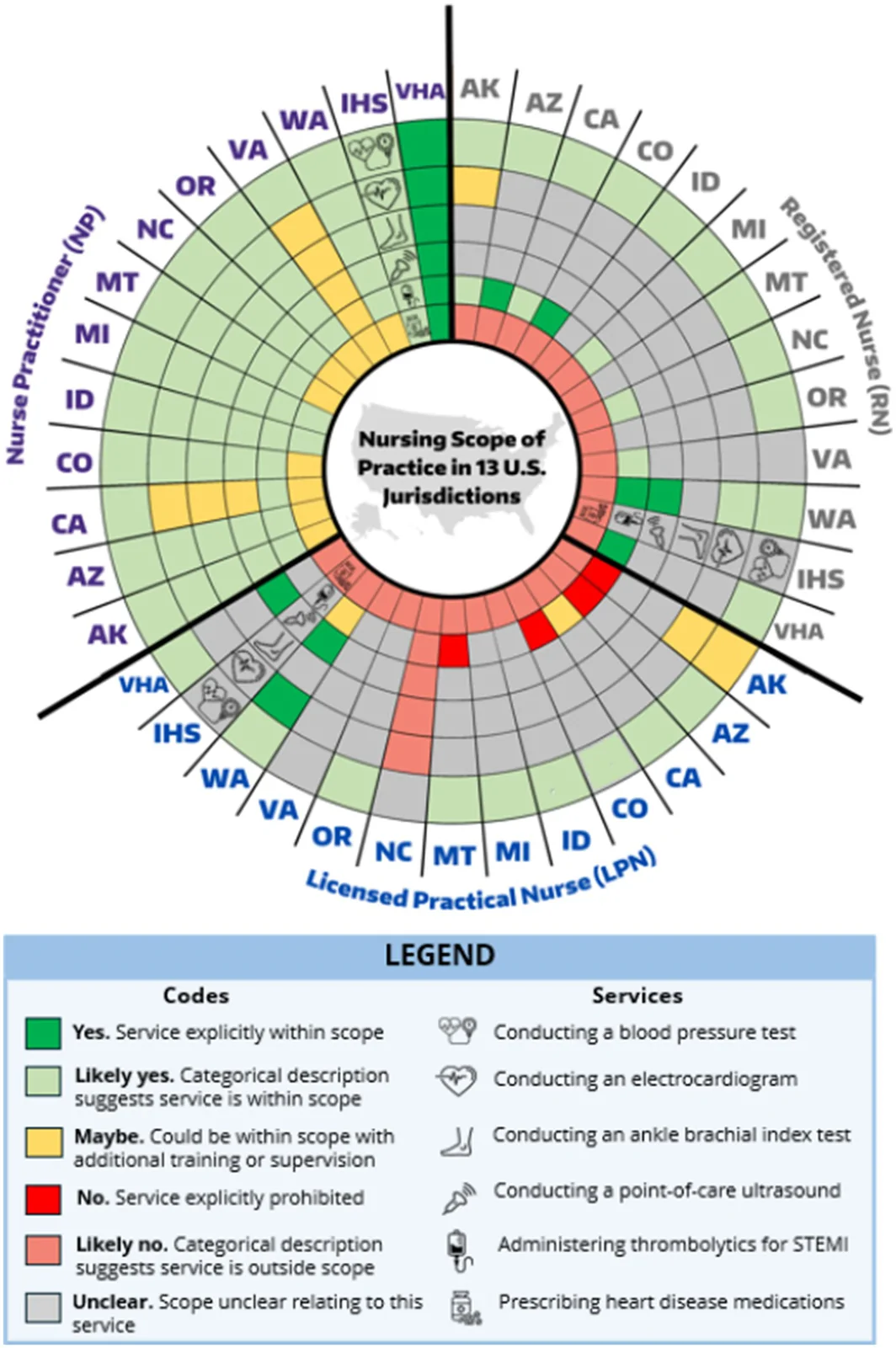
Full scope of practice coding results for nursing professions, effective July 1, 2024.

#### Ambiguity in Scopes of Practice

For cadres with identified licensed scopes of practice, only 18% of the domains were sufficiently clear to be coded as Yes or No (13% as No, n=107/832; 5% as Yes, n=43/832) (Figure 3). Notably, a large percentage of all domains had to be coded as Unclear (17%, n=157 /936) because of the ambiguous or general language used in the licensing statutes and how that language applies to a specific cardiovascular health service. The percentage of Unclear codes varied by cadre. The greatest percentages of unclear codes were for RNs, MAs, and LPNs performing moderately complex diagnostic tests (ie, ankle brachial index test, ECG, and ultrasound screening), with 92%, 85%, and 79% of the jurisdictions, respectively, being coded as Unclear. The cadres with the lowest percentage of Unclear were NPs, for whom no services were coded as Unclear. EMTs and paramedics also had a low percentage of Unclear codes (3%, n=5/176), largely attributable to the fact many jurisdictions had adopted detailed prehospital treatment protocols to define the scopes of practice of EMTs and paramedics.

**Figure 3:**
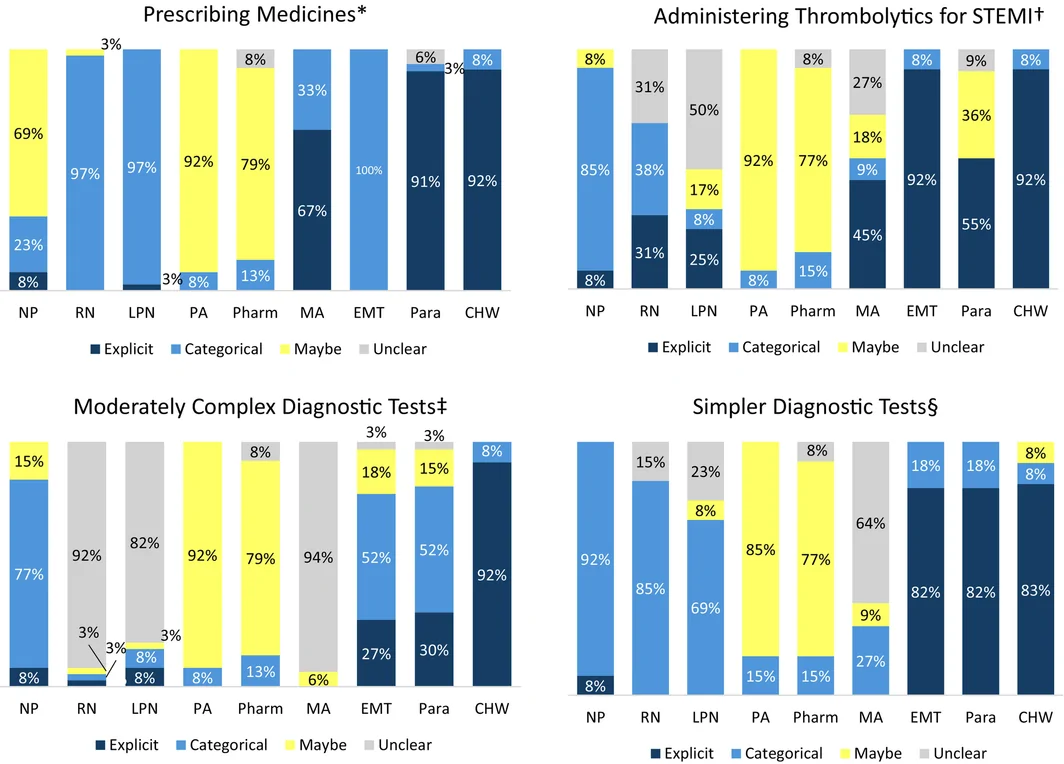
Clarity in scope of practice for heart disease services, effective July 1, 2024. CHW indicates community health worker; EMT, emergency medical technician; LPN, licensed practical nurse; MA, medical assistant; NP, nurse practitioner; PA, physician assistant; Para, paramedic; Pharm, pharmacist; and RN, registered nurse. *Includes the following services: prescribing antihypertensive medicines, prescribing heart failure medicines, and monitoring International Normalized Ratio (INR) and adjusting anticoagulant medicine dosing. ^†^Includes the following service: intravenous administration of thrombolytics for ST‐segment–elevation myocardial infarction (STEMI). ^‡^Includes the following service: conducting point of care ultrasound screening for heart disease, conducting an ankle brachial index test, and conducting an ECG. ^§^Includes the following service: conducting a blood pressure test.

#### Delegated Authority

The scopes of practice of some cadres were heavily reliant on receiving delegated authority from a physician or other licensed clinician. The most formal type of delegated authority was through delegated authority agreements (ie, collaborative practice agreements) between (1) PAs, pharmacists, or (less frequently) NPs and (2) physicians. PAs and pharmacists can potentially have broad scopes of practice, but only if those services are included in a delegated authority agreement with a physician; this occurred across most of the jurisdictions in our analysis, causing most domains for PAs and pharmacists to be coded as Maybe (85%, n=176/208), because authority to perform this service in most states in our sample is conditional on the scope of a collaborative practice agreement. In practice, the scope of authority of PAs under collaborative practice agreements will often include some level of diagnosing and prescribing under the supervision of a physician.^28^ In addition to formal delegated authority agreements, delegation was heavily referenced for MAs whose licensed scope of practice depends on delegation from licensed health workers and can vary at the discretion of the delegating health worker. Some jurisdictions also allowed state or county EMS medical directors to expand the licensed scope of practice for EMTs and paramedics. Instances where additional education or training were needed for other cadres were also coded as Maybe, such as NPs needing to apply for prescriptive authority, or EMTs needing additional training to conduct an ECG. Overall, Maybe, accounted for one‐fourth (26%, n=243/936) of all codes.

#### Jurisdiction Alignment Scores

The full Jurisdiction Alignment Scores are available as Table S9 of the supplemental files and varied by cadre. CHWs had the highest average alignment scores (0.99) because they lack a defined licensed scope of practice in most jurisdictions, and, therefore, almost all services were coded as No or Likely no. PAs also had high average alignment scores, mostly attributable to the large number of jurisdictions coded as Maybe because of delegated authority agreements (0.91). EMTs and RNs both had slight variation across services and high average alignment scores (both 0.90). NPs had one of the lowest average alignment scores of all cadres (0.82), illustrating differences in independent practice authority and jurisdictions with additional requirements for prescriptive authority. LPNs and MAs also had low average alignment scores (0.83 and 0.85, respectively), illustrating variation in the clarity of licensed scope of practice between jurisdictions. Pharmacists had all domains coded as Maybe in 10 jurisdictions, but the variation in Idaho (all Likely yes), Michigan (all Unclear), and North Carolina (7/8 Likely no) results in pharmacists having the lowest average jurisdiction alignment score (0.78).

## DISCUSSION

Critical health workforce shortages in the rural United States call for innovative solutions that integrate multiple cadres of health workers. Nonphysician health workers have an important role in expanding access to care in rural and underserved communities.^29^, ^30^, ^31^, ^32^, ^33^, ^34^ For example, 90% of Americans live within 5 miles of a community pharmacy,^35^ and individuals in rural communities visit community pharmacies significantly more often than they visit a primary care provider.^36^ Implementing team‐based care models requires a clear understanding of how scope of practice regulations define the services that health workers are authorized to provide. Our analysis found substantial variation, ambiguity, and gaps in how jurisdictions defined licensed scope of practice, which may undermine the clarity of the regulatory environment and consequently the ability of health workers to engage in collaborative team‐based heart disease care models. The level of detail sometimes varied substantially between jurisdictions and/or cadres within the same jurisdiction. For example, the licensed scope of practice for an RN in North Carolina was defined generally as, “Assessing the patient's physical and mental health, including the patient's reaction to illnesses and treatment regimens,” whereas the licensed scope of practice for EMS workers in North Carolina included a list of more than a hundred procedures and medications and indicated whether each fell within the licensed scope of practice of each of the four levels of EMS worker. Furthermore, PA and pharmacist licensed scopes of practice relating to heart disease prevention and treatment largely relied on the scope of collaborative practice agreements, the requirements of which also vary across jurisdictions. This lack of clarity and consistency of roles across settings and jurisdictions may undermine role optimization and lead to misalignment between actual service provision, training curricula, and quality oversight. In practice, patient care could be negatively impacted if providers are hesitant to provide necessary services that are within their education and training but not clearly defined in licensed scope of practice.

In addition to the traditional hospital or clinic‐based setting, there is a growing appreciation for the importance of community‐based health services.^37^, ^38^ While EMT and paramedic scopes of practice were often very detailed, they lacked clarity about emergent versus non‐emergent services which could hinder the implementation of community‐based EMS programs.^39^, ^40^ CHWs did not have defined licensed scopes of practice in any of our sample jurisdictions, potentially undermining their ability to deliver or seek reimbursement for basic heart disease screening or monitoring procedures, such as measuring blood pressure. While there have been efforts to increase the number of states that offer CHW certification,^37^ there are differing perspectives about whether certification would create barriers to entering the profession.^41^ This highlights how even the decision whether to define scope of practice can be an important policy question for some cadres of health workers.

Evidence suggests that nonphysician health workers can safely and effectively provide chronic disease management, including heart disease. Pharmacist‐and nurse‐led heart failure programs showed improved initiation and optimization of guideline‐directed medical therapy,^42^ and an international review of independent pharmacist prescribing found improved management of heart disease risk factors.^43^ Additional evidence from broader care models is consistent with these findings, as reviews have found that both NPs^44^ and PAs^45^ achieve clinical outcomes comparable to physicians, and independent practice authority for NPs has been shown to reduce avoidable hospitalizations for chronic conditions^46^ and decrease emergency room use.^47^

Based on these findings, we present 3 policy opportunities that may help address the ambiguity in licensed scope of practice laws and facilitate team‐based heart disease care models, particularly in rural areas.

### More Closely Link Licensed Scope of Practice Definitions With Completion of Accredited Training Programs

States could clarify definitions of licensed scopes of practice for health workers by explicitly linking licensed scope of practice definitions with the scope of accredited health worker training curricula, where available. Health care is going through a period of rapid change with new evidence changing how patients are cared for and new technologies (eg, artificial intelligence‐supported diagnostics, virtual care platforms, wearable and mobile health devices) changing where and who can provide that care. These changes make maintaining clarity and timeliness of licensed scope of practice definitions in statute extremely challenging. For this reason, it may be time to jettison the traditional frameworks for defining licensed scope of practice and instead adopt a “standard of care” or “full extent of education or training” framework whereby the health worker's licensed scope of practice is defined by their level of training and education. For example, Idaho has adopted this type of framework for pharmacists and Oregon has adopted this framework for NPs. Requiring the successful completion of accredited training programs would ensure that health workers have the minimum training needed to protect life and safety, which is the typical standard for licensure requirements. Linking licensed scope of practice with individual training experience would reinforce the relationship between education, training, and team roles, and remove the ambiguity that arises from outdated and often opaque statutory definitions.

### Shift Away From Reliance on Collaborative Practice Agreements

Jurisdictions could consider shifting away from collaborative practice agreements to define or supplement licensed scope of practice for health worker cadres (eg, NPs, PAs, and pharmacists) and instead link licensed scope of practice definitions to demonstrated skill‐based competencies evidenced through successful completion of accredited training programs. Collaborative practice agreements can be a useful mechanism to establish referral networks, but they can also create barriers to team‐based care by increasing administrative cost and burden^48^, ^49^ and restrict licensed scope of practice below the full extent of training. Furthermore, health workers in rural areas may not have access to physicians who are willing to enter into collaborative practice agreements and some states have restrictions on the distance a health worker can be from the collaborating physician.^50^, ^51^

### Prohibit Insurance Plans From Conditioning Reimbursement Based Solely on Provider Cadre

Ambiguity in licensed scope of practice may contribute to insurance plans instituting their own cadre restrictions which in turn can undermine the financial viability of team‐based care models. Because rural health systems rely heavily on nonphysician health workers, reimbursement policies that condition reimbursement on provider cadre may disproportionately affect access to heart disease care in rural communities. If states were to standardize licensed scope of practice definitions in a way that reduced ambiguity (eg, by linking licensed scope of practice to accredited training curricula), states could consider adopting laws prohibiting insurance plans from conditioning reimbursement based on a service being provided by a specific cadre, as long as the provider is in‐network and the service is within the provider's licensed scope of practice. This could include allowing clinicians, such as pharmacists, to bill under medical benefit plans as long as the service is included in their pre‐ or in‐service accredited training. Multiple states have adopted laws requiring reimbursement for some services provided by some cadres. For example, some states have adopted laws requiring commercial payors to recognize pharmacists as health providers, allowing them to bill directly for a broader range of health services, while other states require reimbursement for community paramedicine services provided by EMTs or paramedics.^52^, ^53^ Establishing an across‐the‐board reimbursement requirement grounded in standardized licensed scope of practice definitions could be simpler to administer than piecemeal approaches and could have far‐reaching benefits to increasing access to heart disease care in rural areas. A systematic review exploring the economics of team‐based blood pressure management programs found them to be cost effective, with a decreased cost of −$140 per patient per year following the intervention.^54^ These findings support payment models that reimburse services delivered by multiple health worker cadres.

This study is not without limitations. The coding of domains relied on our study team's interpretation of state and federal law, but we used legal epidemiology methods to help improve the reliability of the coding decisions. Furthermore, as highlighted above, state and federal laws that define licensed scope of practice are only one component of a health worker's effective scope of practice. In addition, our review included a purposively selected sample of jurisdictions and was cross‐sectional, capturing the law at a single point in time. As a result, the analysis does not capture longitudinal changes in licensed scope of practice and therefore cannot assess policy variation across jurisdictions as natural experiments to evaluate the effects of scope of practice laws on care delivery or health outcomes.

## CONCLUSIONS

Addressing disparities in heart disease outcomes will require an optimized and diverse health workforce that can meet the wide range of prevention, screening, diagnosis, and treatment needs of rural communities. Our analysis found that licensed scope of practice laws in a sample of jurisdictions frequently do not provide the clarity needed to establish team‐based care models that allow members to practice to the full extent of their education and training. To address this challenge, we encourage states to consider 3 policy opportunities: (1) link licensed scope of practice definitions to the scope of education and accredited training curricula; (2) shift away from relying on collaborative practice agreements to define or supplement definitions of licensed scope of practice; and (3) prohibit insurance plans from conditioning reimbursement based solely on provider cadre. Recommending modifications to licensed scope of practice laws is often politically contentious and some of the ambiguity in these laws may be attributable to political compromise. Yet, licensed scope of practice laws is now a significant barrier to scaling innovative team‐based care models, and we hope these findings and policy opportunities can move the conversation forward in rethinking how we define the health worker licensed scope of practice to improve heart disease care in rural America.

## Sources of Funding

This work was supported by American Heart Association/https://doi.org/10.58275/AHA.23HERNCRH1076858.pc.gr.173734/University of Washington School of Medicine/2025–2026.

## Disclosures

The authors have no disclosures to report.

## Supporting information

Tables S1–S9
References 55–88

## References

[1] CDC . Heart disease facts. Heart disease [Internet]. 2025. Accessed June 17, 2025. https://www.cdc.gov/heart‐disease/data‐research/facts‐stats/index.html.

[2] Curtin S , Spencer MR . Trends in death rates in urban and rural areas: United States, 1999–2019 [Internet]. National Center for Health Statistics (U.S); 2021. doi: 10.15620/cdc:109049 34582331

[3] Centers for Disease Control and Prevention, National Center for Health Statistics . Heart disease deaths [Internet]. 2024. Accessed January 14, 2025. https://www.cdc.gov/nchs/hus/topics/heart‐disease‐deaths.htm.

[4] Centers for Disease Control and Prevention . Lower your risk for the number 1 killer of women. Women's health [Internet]. 2024. Accessed January 14, 2025. https://www.cdc.gov/womens‐health/features/heart‐disease.html.

[5] Murphy S , Kochanek K , Xu J , Arias E . Mortality in the United States, 2020 [Data Brief] [Internet]. Data Brief no.: 427. Hyattsville, MD: National Center for Health Statistics (U.S.); 2021. doi: 10.15620/cdc:112079

[6] Glynn P , Lloyd‐Jones Donald M , Feinstein MJ , Carnethon M , Khan SS . Disparities in cardiovascular mortality related to heart failure in the United States. JACC. 2019;73:2354–2355. doi: 10.1016/j.jacc.2019.02.042 31072580

[7] Aggarwal R , Chiu N , Loccoh EC , Kazi DS , Yeh RW , Wadhera RK . Rural‐urban disparities: diabetes, hypertension, heart disease, and stroke mortality among Black and White adults, 1999–2018. J Am Coll Cardiol. 2021;77:1480–1481. doi: 10.1016/j.jacc.2021.01.032 PMC8210746 33736831

[8] CDC . Heart disease risk factors. Heart disease [Internet]. 2024. Accessed April 15, 2025. https://www.cdc.gov/heart‐disease/risk‐factors/index.html.

[9] CDC . About air quality. Air quality [Internet]. 2025. Accessed July 9, 2025. https://www.cdc.gov/air‐quality/about/index.html.

[10] Department of Health and Human Services , Health Resources and Services Administration , National Center for Health Workforce Analysis . Workforce projections [Internet]. Accessed January 14, 2025. https://data.hrsa.gov/topics/health‐workforce/workforce‐projections.

[11] DiPiro JT , Hoffman JM , Tichy E , Shea S , Sanborn M , Hung A , Fox ER , Watanabe JH , Torrise V , Abourjaily P , et al. ASHP and ASHP Foundation pharmacy forecast 2025: strategic planning guidance for pharmacy departments in hospitals and health systems. Am J Health‐Syst Pharm. 2025;82:17–47. doi: 10.1093/ajhp/zxae280 39657700

[12] National Center for Health Workforce Analysis . Nurse workforce projections, 2022‐2037 [Internet]. Health & Resources Services Administration; 2024. Accessed February 20, 2025. https://bhw.hrsa.gov/sites/default/files/bureau‐health‐workforce/data‐research/nursing‐projections‐factsheet.pdf

[13] Cash RE , Rivard MK , Chrzan K , Mercer CB , Camargo CA Jr , Panchal AR . Comparison of volunteer and paid EMS professionals in the United States. Prehosp Emerg Care. 2021;25:205–212. doi: 10.1080/10903127.2020.1752867 32271639

[14] Health Resources and Services Administration, National Advisory Committee on Rural Health and Human Services . Access to emergency medical services in rural communities ‐ policy brief and recommendations to the secretary [Policy Brief]. 2022.

[15] Andrilla C , Woolcock S , Garberson L , Keppel G , Graves J , Patterson D . Trends in the health workforce supply in the rural U.S. [Policy Report] [Internet]. 2024. https://familymedicine.uw.edu/rhrc/publications/trends‐in‐the‐health‐workforce‐supply‐in‐the‐rural‐u‐s/.

[16] Joshi R , Thrift AG , Smith C , Praveen D , Vedanthan R , Gyamfi J , Schwalm JD , Limbani F , Rubinstein A , Parker G , et al. Task‐shifting for cardiovascular risk factor management: lessons from the global alliance for chronic diseases. BMJ Glob Health. 2018;3(Suppl 3):e001092. doi: 10.1136/bmjgh-2018-001092 PMC6231102 30483414

[17] Irazola V , Prado C , Rosende A , Flood D , Tsuyuki R , Ojeda CN , Villatoro Reyes M , Otero J , Wellmann IA , Fajardo I , et al. Expanding team‐based care for hypertension and cardiovascular risk management with HEARTS in the Americas. Rev Panam Salud Pública. 2025;12:1. doi: 10.26633/rpsp.2025.43 PMC12065422 40357407

[18] CDC . Team‐based care to improve blood pressure control. High blood pressure [Internet]. 2025. Accessed April 18, 2025. https://www.cdc.gov/high‐blood‐pressure/php/data‐research/team‐based‐care/index.html.

[19] Guide to Community Preventive Services . Heart disease & stroke prevention: team‐based care to improve blood pressure control [Internet]. 2025. Accessed July 9, 2025. https://www.thecommunityguide.org/findings/heart‐disease‐stroke‐prevention‐team‐based‐care‐improve‐blood‐pressure‐control.html.

[20] Brault I , Kilpatrick K , D'Amour D , Contandriopoulos D , Chouinard V , Dubois CA , Perroux M , Beaulieu MD . Role clarification processes for better integration of nurse practitioners into primary healthcare teams: a multiple‐case study. Nurs Res Pract. 2014;2014:1–9. doi: 10.1155/2014/170514 PMC4322308 25692039

[21] Torrens C , Campbell P , Hoskins G , Strachan H , Wells M , Cunningham M , Bottone H , Polson R , Maxwell M . Barriers and facilitators to the implementation of the advanced nurse practitioner role in primary care settings: a scoping review. Int J Nurs Stud. 2020;104:103443. doi: 10.1016/j.ijnurstu.2019.103443 32120089

[22] Lambert LK , Havaei F , Beck SM , Ma A , Larmet J , Kaur J , Adhami N , Le D , Woods R . An early evaluation of team consistency and scope optimization in team‐based cancer care. BMC Cancer. 2025;25:371. doi: 10.1186/s12885-025-13644-9 PMC11869398 40022018

[23] Martin B , Reneau K . How collaborative practice agreements impede the Administration of Vital Women's Health Services. J Midwifery Womens Health. 2020;65:487–495. doi: 10.1111/jmwh.13088 32277575

[24] Salgado TM , Rosenthal MM , Coe AB , Kaefer TN , Dixon DL , Farris KB . Primary healthcare policy and vision for community pharmacy and pharmacists in the United States. Pharm Pract. 2020;18:2160. doi: 10.18549/PharmPract.2020.3.2160 PMC7523559 33029264

[25] Downie S , Walsh J , Kirk‐Brown A , Haines TP . How can scope of practice be described and conceptualised in medical and health professions? A systematic review for scoping and content analysis. Int J Health Plann Manage. 2023;38:1184–1211. doi: 10.1002/hpm.3678 37434288

[26] Burris S , Ashe M , Levin D , Penn M , Larkin M . A transdisciplinary approach to public health law: the emerging practice of legal epidemiology. Annu Rev Public Health. 2016;37:135–148. doi: 10.1146/annurev-publhealth-032315-021841 PMC5703193 26667606

[27] Lane J , Garrison MM , Kelley J , Sarma P , Katz A . Strengthening policy coding methodologies to improve COVID‐19 disease modeling and policy responses: a proposed coding framework and recommendations. BMC Med Res Methodol. 2020;20:298. doi: 10.1186/s12874-020-01174-w PMC7721792 33292170

[28] What is a PA? AAPA [Internet]. Accessed April 17, 2026. https://www.aapa.org/about/what‐is‐a‐pa/.

[29] Doescher MP , Andrilla CHA , Skillman SM , Morgan P , Kaplan L . The contribution of physicians, physician assistants, and nurse practitioners toward rural primary care: findings from a 13‐state survey. Med Care. 2014;52:549–556. doi: 10.1097/MLR.0000000000000135 24824539

[30] Barnes H , Richards MR , McHugh MD , Martsolf G . Rural and nonrural primary care physician practices increasingly rely on nurse practitioners. Health Aff (Proj Hope). 2018;37:908–914. doi: 10.1377/hlthaff.2017.1158 PMC6080248 29863933

[31] DePriest K , D'Aoust R , Samuel L , Commodore‐Mensah Y , Hanson G , Slade EP . Nurse practitioners' workforce outcomes under implementation of full practice authority. Nurs Outlook. 2020;68:459–467. doi: 10.1016/j.outlook.2020.05.008 PMC7581487 32593462

[32] Berini CR , Bonilha HS , Simpson AN . Impact of community health workers on access to care for rural populations in the United States: a systematic review. J Community Health. 2022;47:539–553. doi: 10.1007/s10900-021-01052-6 34817755

[33] Chan J , Griffith LE , Costa AP , Leyenaar MS , Agarwal G . Community paramedicine: a systematic review of program descriptions and training. CJEM. 2019;21:749–761. doi: 10.1017/cem.2019.14 30885280

[34] Patterson DG , Coulthard C , Garberson LA , Wingrove G , Larson EH . What is the potential of community paramedicine to fill rural health care gaps? J Health Care Poor Underserved. 2016;27(4A):144–158. doi: 10.1353/hpu.2016.0192 27818420

[35] Berenbrok LA , Gabriel N , Coley KC , Hernandez I . Evaluation of frequency of encounters with primary care physicians vs visits to community pharmacies among Medicare beneficiaries. JAMA Netw Open. 2020;3:e209132. doi: 10.1001/jamanetworkopen.2020.9132 PMC7364370 32667653

[36] Berenbrok LA , Tang S , Gabriel N , Guo J , Sharareh N , Patel N , Dickson S , Hernandez I . Access to community pharmacies: a nationwide geographic information systems cross‐sectional analysis. J Am Pharm Assoc. 2022;62:1816–1822.e2. doi: 10.1016/j.japh.2022.07.003 35965233

[37] Malcarney MB , Pittman P , Quigley L , Horton K , Seiler N . The changing roles of community health workers. Health Serv Res. 2017;52(S1):360–382. doi: 10.1111/1475-6773.12657 PMC5269544 28127766

[38] National Association of Emergency Medical Technicians (NAEMT) . 2023 National survey on MIH‐CP programs [Internet]. 2023. Accessed April 18, 2025. https://naemt.org/docs/default‐source/community‐paramedicine/toolkit/mihcp‐presentation_may‐2023.pdf.

[39] Glenn M , Zoph O , Weidenaar K , Barraza L , Greco W , Jenkins K , Paode P , Fisher J . State regulation of community paramedicine programs: a national analysis. Prehosp Emerg Care. 2018;22:244–251. doi: 10.1080/10903127.2017.1371260 29023167

[40] Gorndt K , Haverly K , Syverson T . Bridging the gap: a policy framework for sustainable community paramedicine in rural America. 2024.

[41] Miller J , Chapman S . Understanding California's community health worker/promotor workforce: a deep dive into CHW/P employer perspectives. The California Health Care Foundation; 2023.

[42] Zheng J , Mednick T , Heidenreich PA , Sandhu AT . Pharmacist‐ and nurse‐led medical optimization in heart failure: a systematic review and meta‐analysis. J Card Fail. 2023;29:1000–1013. doi: 10.1016/j.cardfail.2023.03.012 PMC10524094 37004867

[43] Vowles K , Garland C , McCarron M , Scott MG , Harrison C . Systematic review of independent pharmacist prescribing for the treatment of cardiometabolic factors in chronic non‐communicable diseases. Eur J Hosp Pharm. 2026. doi: 10.1136/ejhpharm-2025-004554. Online ahead of print. PMC13725632 41748318

[44] Stanik‐Hutt J , Newhouse RP , White KM , Johantgen M , Bass EB , Zangaro G , Wilson R , Fountain L , Steinwachs DM , Heindel L , et al. The quality and effectiveness of care provided by nurse practitioners. J Nurse Pract. 2013;9:492–500.e13. doi: 10.1016/j.nurpra.2013.07.004

[45] Cooper N , Agius S , Freeman K , Church H , Yoosoof F , Frain A , Frain J , McConnell R , Wilson E , Leonardi‐Bee J . Impact of physician assistants on quality of care: rapid review. BMJ. 2025;3:e086358. doi: 10.1136/bmj-2025-086358 PMC12225544 40610033

[46] McMichael BJ . The impact of nurse practitioner scope‐of‐practice laws on preventable hospitalizations. J Health Econ. 2025;1:103044. doi: 10.1016/j.jhealeco.2025.103044 40753838

[47] Traczynski J , Udalova V . Nurse practitioner independence, health care utilization, and health outcomes. J Health Econ. 2018;1:90–109. doi: 10.1016/j.jhealeco.2018.01.001 29475093

[48] Martin B , Alexander M . The economic burden and practice restrictions associated with collaborative practice agreements: a national survey of advanced practice registered nurses. J Nurs Regul. 2019;9:22–30. doi: 10.1016/S2155-8256(19)30012-2

[49] Lott BE , Anderson EJ , Villa Zapata L , Cooley J , Forbes S , Taylor AM , Manygoats T , Warholak T . Expanding pharmacists' roles: pharmacists' perspectives on barriers and facilitators to collaborative practice. J Am Pharm Assoc. 2021;61:213–220.e1. doi: 10.1016/j.japh.2020.11.024 33359117

[50] Markowitz S , Adams EK , Lewitt MJ , Dunlop AL . Competitive effects of scope of practice restrictions: public health or public harm? J Health Econ. 2017;1:201–218. doi: 10.1016/j.jhealeco.2017.07.004 28778349

[51] Adams AJ , Eid DD . Toward collaborative practice, not collaborative practice agreements. Am J Health‐Syst Pharm. 2024;81:e157–e158. doi: 10.1093/ajhp/zxad280 37948606

[52] Bell N , Hughes R , Fede ALD . Collaborative practice agreements and their geographic impact on where nurse practitioners can practice. J Nurs Regul. 2018;9:5–14. doi: 10.1016/S2155-8256(18)30149-2

[53] Wittenauer R , Bacci JL , Shah PD , Stergachis A . Vaccination payments in states with provider status for pharmacists: a claims analysis. J Am Pharm Assoc. 2025;65:102301. doi: 10.1016/j.japh.2024.102301 39581347

[54] Jacob V , Reynolds JA , Chattopadhyay SK , Nowak K , Hopkins DP , Fulmer E , Bhatt AN , Therrien NL , Cuellar AE , Kottke TE , et al. Economics of team‐based care for blood pressure control: updated community guide systematic review. Am J Prev Med. 2023;65:735–754. doi: 10.1016/j.amepre.2023.04.013 PMC10527860 37121447

[55] State of Alaska Department of Health and Social Services . State of Alaska EMS scope of practice document [Internet]. 2018. Accessed June 1, 2024. https://aws.state.ak.us/OnlinePublicNotices/Notices/Attachment.aspx?id=111777.

[56] Matanuska‐Susitna Borough Department of Emergency Services . EMS protocols & procedures. Adult & pediatric [Internet]. 2017. Accessed June 4, 2024. https://matsugov.us/docs/general/18549/emsstanding_orders_17.pdf.

[57] Community Health Aide Program Alaska . Community Health Aide Program certification board standards and procedures [Internet]. 2023. Accessed June 2, 2024. https://akchap.org/wp‐content/uploads/2023/06/CHAPCB‐Standards‐Procedures‐Amended‐2023‐6‐8.pdf.

[58] Alaska State Medical Board . Delegating to medical assistants (unlicensed assistive personnel). Board issues guidelines [Internet]. 2016. Accessed June 1, 2024. https://www.aama‐ntl.org/docs/default‐source/legal/ak‐bomed‐guide‐delegation.pdf?sfvrsn=2.

[59] Arizona Office of the Secretary of State R . Arizona Administrative Code. Title 9 health services. Chapter 25 Department of Health Services ‐ Emergency Medical Services [Internet]. 2024. Accessed June 1, 2024. https://apps.azsos.gov/public_services/Title_09/9‐25.pdf.

[60] Arizona State Board of Nursing . Advisory opinion ultrasounds: limited obstetric, gynecologic, and reproductive [Internet]. 2018. Accessed April 24, 2024. https://web.archive.org/web/20220310224604/https://www.azbn.gov/sites/default/files/advisory‐opinions/ao‐ultrasounds‐limited‐ob‐gyn‐and‐reproductive‐rev‐january‐2018.pdf.

[61] Arizona State Board of Nursing . Advisory opinion infusion therapy/venipuncture: the role of the licensed practical nurse [Internet]. 2016. Accessed April 24, 2024. https://web.archive.org/web/20220310224512/https://www.azbn.gov/sites/default/files/advisory‐opinions/ao‐infusiontherapy‐venipuncture‐the‐role‐of‐the‐lpn‐rev92016.pdf.

[62] U.S. Department of Health and Human Services . The Indian Health Manual (IHM). Chapter 4 ‐ Nursing. Accessed October 16, 2024. https://www.ihs.gov/ihm/pc/part‐3/p3c4/#3‐4.1E.

[63] U.S. Department of Health and Human Services . The Indian Health Manual (IHM). Chapter 28 ‐ Physician assistants | Part 3. 2019. Accessed June 1, 2024. https://www.ihs.gov/ihm/pc/part‐3/p3c28/.

[64] U.S. Department of Health and Human Services . The Indian Health Manual (IHM). Chapter 7 ‐ Pharmacy | Part 3. 2023. Accessed October 24, 2024. https://www.ihs.gov/ihm/pc/part‐3/p3c7/.

[65] Division of Behavioral Health, Indian Health Service . Community Health Representative (CHR) fact sheet.

[66] Indian Health Service . Division of Clinical and Community Services. Emergency Medical Services. Accessed October 24, 2024. https://www.ihs.gov/dccs/ems/.

[67] Secretary of State, State of Colorado B. Department of Public Health and Environment . Health facilities and emergency medical services division. Emergency Medical Services; 2018. Accessed June 1, 2024. https://www.sos.state.co.us/CCR/GenerateRulePdf.do?ruleVersionId=7408&fileName=6%20CCR%201015‐3

[68] Idaho Department of Health & Welfare . EMS physician coalition [Internet]. Accessed June 5, 2024. https://healthandwelfare.idaho.gov/about‐dhw/boards‐councils‐committees/ems‐physician‐commission.

[69] Scope of practice – Michigan Society of Medical Assistants [Internet]. Accessed June 1, 2024. https://msmaonline.org/scope‐of‐practice/.

[70] Bureau of Emergency Preparedness, EMS and Systems of Care, Michigan Department of Health & Human Services. Michigan EMS Scope of Practice [Internet]. 2024. Accessed June 1, 2024. https://www.michigan.gov/mdhhs/‐/media/Project/Websites/mdhhs/Inside‐MDHHS/Policy‐and‐Planning/EMS/New‐Uploads/23‐5‐30/24410‐Michigan‐EMS‐Scope‐of‐Practice‐042024.pdf?rev=23c27d56a31d4058a1ce0c7ea3d2b767&hash=AD7F7DC1369BD70E13D18E4F2F23ADEC.

[71] US Department of Veterans Affairs, Veterans Health Administration . VHA Directive 1063: utilization of physician assistants (PA) [Internet]. Accessed October 22, 2024. https://www.patientcare.va.gov/PA/DIRECTIVES_HANDBOOKS.asp.

[72] Department of Veterans Affairs . VHA handbook 1108.11(4). Clinical Pharmacy Services. 2024.

[73] Department of Veterans Affairs . VA handbook 5005/160. Staffing. 2024.

[74] Department of Veterans Affairs . VA nursing facts [Internet]. 2019. Accessed October 1, 2024. https://www.va.gov/nursing/docs/about/vansgfacts_www.doc.

[75] US Department of Veterans Affairs, Veterans Health Administration . VHA Directive 1108.13(1). Provision and use of nursing medication management protocols in outpatient team‐based practice settings. 2019.

[76] Department of Veterans Affairs . VA national standards of practice [Internet]. Accessed October 1, 2024. https://www.va.gov/STANDARDSOFPRACTICE/faq.asp.

[77] Montana Board of Medical Examiners . Montana prehospital treatment protocols [Internet]. 2020. Accessed June 6, 2024. https://boards.bsd.dli.mt.gov/medical‐examiners/emergency‐care‐provider/instructor/Curricula‐Practice‐Guidelines.

[78] North Carolina Medical Board . Delegating medical tasks to unlicensed personnel [Internet]. 2024. Accessed June 6, 2024. https://www.ncmedboard.org/resources‐information/professional‐resources/publications/forum‐newsletter/article/delegating‐medical‐tasks‐to‐unlicensed‐personnel.

[79] The North Carolina Office of EMS . North Carolina Medical Board approved medications for credentialed EMS personnel [Internet]. 2021. Accessed June 1, 2024. https://www.ncems.org/pdf/NCCEPandNCMB‐List‐Combined‐Revised.pdf.

[80] The North Carolina Office of EMS . North Carolina College of Emergency Physicians standards for medical oversight and data collection [Internet]. Accessed June 6, 2024. https://www.ncems.org/nccepstandards.html.

[81] Oregon Medical Board . Oregon Medical Board: use of unlicensed healthcare personnel: statements of philosophy: State of Oregon [Internet]. Accessed June 1, 2024. https://www.oregon.gov/omb/board/philosophy/pages/use‐of‐unlicensed‐healthcare‐personnel.aspx.

[82] Virginia Board of Nursing . Scope of practice for registered nurses and licensed practical nurses [Internet]. 2023. https://www.dhp.virginia.gov/media/dhpweb/docs/nursing/guidance/90‐6.pdf.

[83] Virginia Office of EMS Regulation & Compliance Enforcement Division . Guidance document scope of practice procedures & formulary. 2022.

[84] Washington State Board of Nursing . Licensed practical nurse | Washington State Board of Nursing [Internet]. Accessed June 2, 2024. https://web.archive.org/web/20240602131502/https://nursing.wa.gov/support‐practicing‐nurses/practice‐information/licensed‐practical‐nurse.

[85] Washington State Board of Nursing . Licensed practical nurse and medical assistant scope of practice comparison [Internet]. 2023. Accessed June 1, 2024. https://nursing.wa.gov/sites/default/files/2023‐05/LPN‐MA‐Scope‐of‐Practice‐comparison.pdf.

[86] Washington State Pharmacy Quality Assurance Commission . Guidance on collaborative drug therapy agreements [Internet]. 2020. Accessed June 2, 2024. https://doh.wa.gov/sites/default/files/legacy/Documents/Pubs/690327.pdf.

[87] Washington State Department of Health HHH . Who may prescribe and administer prescriptions in Washington State | Washington State Department of Health [Internet]. Accessed June 5, 2024. https://doh.wa.gov/licenses‐permits‐and‐certificates/facilities‐z/pharmacies‐and‐pharmaceutical‐firms/who‐may‐prescribe‐and‐administer‐prescriptions‐washington‐state.

[88] Washington State Department of Health . Approved procedures and skills for certified EMS providers [Internet]. 2016. Accessed June 2, 2024. https://www.whitmancountyems.org/images/Documents/WA‐Procedures‐and‐Skills‐June‐2016.pdf.

