# Supplementary material for: Scope of Practice Laws Affecting Team‐Based Heart Disease Care in Rural America: Policy Challenges and Opportunities
Source: J Am Heart Assoc. 2026 Sep 24;15(19):e049493. doi: 10.1161/JAHA.126.049493 (PMC13745156; doi:10.1161/JAHA.126.049493)
Supplement: Supplementary file 1 [file JAH3-15-e049493-s001.pdf]

Appendix A. Codebook and Coding Guidelines

Table S1. Codebook for Prescribing Antihypertensives and Heart Failure Medicines

Code	Definition
Yes	Specifically states that cadre has prescribing authority and lists one or more antihypertensives or specifically mentions antihypertensives and/or heart failure medicines
Likely Yes	Prescribing medicines explicitly mentioned as within scope, but antihypertensives and heart failure medicines not listed specifically
Maybe	(1) States that prescribing medicines is within scope but requires additional training, approval, or supervision or<br>(2) For pharmacists, physician assistants, and nurse practitioners may be able to prescribe but only if included in collaborative practice agreement with supervising physician
Unclear	Major point of ambiguity regarding prescribing authority that prevents applying any other codes with confidence
Likely No	Legal scope of practice but no mention of prescribing authority (e.g., no mention of prescribing authority in medical assistant with a legal scope of practice, licensed practical nurse, or registered scope of practice)
No	(1) Specifically states that cadre does not have prescribing authority; or<br>(2) No legal scope of practice

Table S2. Codebook for Monitoring INR and Modifying Anticoagulant Rx in Anticoagulation Clinics

Code	Definition
Yes	Specifically states that cadre has prescribing authority and lists warfarin
Likely Yes	Prescribing medicines explicitly mentioned as within scope, but warfarin not listed specifically
Maybe	(1) States that prescribing medicines is within scope but requires additional training, approval, or supervision; or<br>(2) For pharmacists, physician assistants, and nurse practitioners: May be able to provide the service but only if included in collaborative practice agreement with supervising physician
Unclear	Major point of ambiguity regarding prescribing authority that prevents applying any other codes with confidence
Likely No	No mention of prescribing authority (e.g., no mention of prescribing authority in medical assistant, licensed practical nurse, or registered nurse scope of practice)
No	(1) Specifically states that cadre does not have prescribing authority; or<br>(2) No legal scope of practice

NOTE: Interpretative Guidance - This service should be interpreted as the ability to write a new prescription to replace an existing prescription (not merely implementing a written order that states dosage should change based on INR)

Table S3. Codebook for Administering Thrombolytics for STEMI

Code	Definition
Yes	Specifically states that cadre can administer thrombolytics
Likely Yes	States that cadre can administer medicines or scope of practice otherwise strongly suggests that cadre can administer medicines, such as the cadre having the authority to prescribe medicines
Maybe	(1) States that cadre can administer medicines but requires additional training, approval, or supervision; or<br>(2) For pharmacists, physician assistants, and nurse practitioners: May be able to provide the service but only if included in collaborative practice agreement with supervising physician
Unclear	Major point of ambiguity that prevents applying any other code with confidence, such as no mention of medications in scope of practice
Likely No	Scope of practice strongly suggests that cadre cannot administer medicines
No	(1) Specifically states that cadre does not have authority to administer thrombolytics<br>(2) Specifically states that cadre cannot administer medicines intravenously; or<br>(3) No licensed scope of practice

Table S4. Codebook for Ultrasound Screening for Heart Disease

Code	Definition
Yes	Specifically states that cadre can conduct ultrasound screening for heart disease
Likely Yes	States that cadre can conduct imaging studies or for nurse practitioners, states that nurse practitioners have broad authority to diagnose illness
Maybe	(1) States that cadre can conduct ultrasound screening but requires additional training, approval, or supervision; or<br>(2) For pharmacists, physician assistants, and nurse practitioners: May be able to provide the service but only if included in collaborative practice agreement with supervising physician
Unclear	(1) Major point or ambiguity that prevents applying other codes with confidence; or<br>(2) For registered nurses, licensed practical nurses, medical assistants: Scope only mentions measuring/monitoring health generally or only mentions taking vitals; or<br>(3) For nurse practitioners: no mention of authority for diagnosis or imaging
Likely No	For all cadres (except nurse practitioners) no mention of conducting screening or diagnostics
No	(1) Specifically states that cadre does not have authority to conduct ultrasound screening for heart disease or (2) no legal scope of practice

Table S5. Codebook for Conducting and Ankle Brachial Index Test (ABI)

Code	Definition
Yes	Specifically states that cadre can conduct ABI
Likely Yes	For nurse practitioners - states that nurse practitioners have broad authority to monitor and diagnose illness (for non-NP cadres see definition in unclear below)
Maybe	(1) States that cadre can conduct ABI but requires additional training, approval, or supervision; or<br>(2) For pharmacists, physician assistants, and nurse practitioners may be able to provide the service but only if included in collaborative practice agreement with supervising physician
Unclear	(1) Major point or ambiguity that prevents applying other codes with confidence; or<br>(2) For registered nurses, licensed practical nurses, medical assistants: Scope only mentions measuring/monitoring health generally or only mentions taking vitals
Likely No	Scope of practice strongly suggests that cadre cannot conduct ABI
No	(1) Specifically states that cadre does not have authority to conduct ABI or (2) no legal scope of practice

Table S6. Codebook for Conducting an Electrocardiogram (ECG/EKG)

Code	Definition
Yes	Specifically states that cadre can conduct and interpret ECG
Likely Yes	For Nurse practitioners: States that Nurse practitioners have broad authority to diagnose and monitor health or illness (see below for other cadres)
Maybe	(1) States that cadre can conduct or interpret ECGs, but only certain kinds (12 vs. 15 leads); or<br>(2) Can only conduct ECGs if undergoes additional training or certification; or<br>(3) For pharmacists, Physician assistants, and Nurse practitioners: May be able to provide the service but only if included in collaborative practice agreement with supervising physician
Unclear	(1) Major point or ambiguity that prevents applying other codes with confidence, such as no mention of ECGs; or<br>(2) For registered nurses, licensed practical nurses, medical assistants: Scope only mentions measuring/monitoring health generally or only mentions taking vitals
Likely No	Scope of practice strongly suggests that cadre cannot conduct ECG, such as scope only including counseling/education or non-clinical services
No	(1) Specifically states that cadre does not have authority to conduct ECG or (2) no legal scope of practice

Table S7. Codebook for Conducting Blood Pressure Test

Code	Definition
Yes	Specifically states that cadre can conduct blood pressure test or take vitals
Likely Yes	States that cadre has authority to measure or monitor health or similar language
Maybe	(1) States that cadre can take blood pressure but requires additional training, approval, or supervision; or<br>(2) For pharmacists, physician assistants, and nurse practitioners: May be able to provide the service but only if included in collaborative practice agreement with supervising physician
Unclear	Major point or ambiguity or no legal scope of practice
Likely No	Scope of practice strongly suggests that cadre cannot take blood pressure, such as scope only including counseling/education or non-clinical services
No	(1) Specifically states that cadre does not have authority to take blood pressure or (2) provide clinical health care services

Table S8. Full Coding Results

Alaska	Prescribing antihypertensives	Prescribing heart failure medicines	Monitoring INR and modifying warfarin Rx	Administering thrombolytics for STEMI	Conducting point of care ultrasound screening for heart disease	Conducting Ankle Brachial Index Test	Conducting ECG	Conducting blood pressure test
Nurse Practitioner	Maybe, but must apply to board of nursing and be approved. 12 AAC 44.440 ("Prescriptive authority"), AS 08.68.850(1) ("Definitions").	Maybe, but must apply to board of nursing and be approved. 12 AAC 44.440 ("Prescriptive authority"), AS 08.68.850(1) ("Definitions").	Maybe, but must apply to board of nursing and be approved. 12 AAC 44.440 ("Prescriptive authority"), AS 08.68.850(1) ("Definitions").	Likely Yes, definition of ARNP states can "perform acts of medical diagnosis and the prescription and dispensing of medical, therapeutics, or corrective measures". AS 08.68.850 ("Definitions")	Likely Yes, can "perform acts of medical diagnosis". AS 08.68.850 ("Definitions")	Likely Yes, definition of ARNP states can "perform acts of medical diagnosis". AS 08.68.850 ("Definitions")	Likely Yes, definition of ARNP states can "perform acts of medical diagnosis". AS 08.68.850 ("Definitions")	Likely Yes, definition of ARNP states can "perform acts of medical diagnosis". AS 08.68.850 ("Definitions")
Registered Nurse	Likely no. No policy found explicitly including this in scope of practice.	Likely no. No policy found explicitly including this in scope of practice.	Likely no. They cannot prescribe, but "medication management for unstable medical conditions requiring ongoing assessment and adjustment of dosage or timing of administration" only referring to "nurse". 12 AAC 44.970. ("Nursing duties that may not be delegated.")	Likely Yes. "Nursing duties that may not be delegated [...] include IV drugs". 12 AAC 44.970 .("Nursing duties that may not be delegated.") AS 08.68.850 ("Definitions")	Unclear [58] "assessment of problems, counseling, and teaching … execution of a medical regimen as prescribed by a person authorized by the state to practice medicine… performance of acts of medical diagnosis…" AS 08.68.850 ("Definitions")	Unclear. "Routine nursing duties that may be delegated include [...] (2) taking and recording vital signs". 12 AAC 44.955. ("Delegation of routine nursing duties") AS 08.68.850 ("Definitions")	Maybe "Specialized nursing tasks that may be delegated include [...] placing electrodes and leads for electrocardiogram" 12 AAC 44.960. ("Delegation of specialized nursing duties.")	Likely Yes. "Routine nursing duties that may be delegated include [...] (2) taking and recording vital signs". 12 AAC 44.955. ("Delegation of routine nursing duties") AS 08.68.850
Licensed Practical Nurse	Likely no. No policy found explicitly including this in scope of practice.	Likely no. No policy found explicitly including this in scope of practice.	No. non delegatable nursing tasks. 12 AAC 44.970. ("Nursing duties that may not be delegated.")	No AS 08.68.850 ("Definitions")	Unclear. AS 08.68.850 ("Definitions")	Unclear. Must work under supervision of another licensed provider. AS. 08.68.265. ("Supervision of practical nurses") AS 08.68.850 ("Definitions")	Maybe "Specialized nursing tasks that may be delegated include [...] placing electrodes and leads for electrocardiogram" 12 AAC 44.960. ("Delegation of specialized nursing duties.") AS 08.68.850 ("Definitions")	Maybe. Must work under supervision of another licensed provider. AS. 08.68.265. ("Supervision of practical nurses") AS 08.68.850 ("Definitions")
Physician Assistant	Maybe - with the authorization of the PA's primary collaborating physician. 12 AAC 40.450. ("Authority to prescribe, order, administer, and dispense medications.")	Maybe - with the authorization of the PA's primary collaborating physician. 12 AAC 40.450. ("Authority to prescribe, order, administer, and dispense medications.")	Maybe - with the authorization of the PA's primary collaborating physician. 12 AAC 40.450. ("Authority to prescribe, order, administer, and dispense medications.")	Maybe, under collaborative practice agreements. 12 AAC 40.430 ("Performance and assessment of practice")	Maybe, under collaborative practice agreements. 12 AAC 40.430 ("Performance and assessment of practice")	Maybe, under collaborative practice agreements. 12 AAC 40.430 ("Performance and assessment of practice")	Maybe, under collaborative practice agreements. 12 AAC 40.430 ("Performance and assessment of practice")	Maybe, under collaborative practice agreements. 12 AAC 40.430 ("Performance and assessment of practice")
Pharmacist	Maybe. 12 AAC 52.240 ("Pharmacist collaborative practice authority")	Maybe. 12 AAC 52.240 ("Pharmacist collaborative practice authority")	Maybe. 12 AAC 52.240 ("Pharmacist collaborative practice authority")	Maybe. AS Sec. 08.80.337. ("Other patient care services")	Maybe. AS Sec. 08.80.337. ("Other patient care services")	Maybe. AS Sec. 08.80.337. ("Other patient care services")	Maybe. AS Sec. 08.80.337. ("Other patient care services")	Maybe. AS Sec. 08.80.337. ("Other patient care services")
Medical Assistant	Likely No [55] MA's considered "unlicensed assistive personnel that perform office and clinical functions"	Likely No [55] MA's considered "unlicensed assistive personnel that perform office and clinical functions"	No. The statute lists "medication management for unstable medical conditions requiring ongoing assessment..." as not to be delegated.. 12 AAC 40.920. ("Standards for delegation of routine duties.")	No. AS 08.68.850 ("Definitions") initiation, administration, and monitoring of intravenous therapy specifically listed as not delegatable. 12 AAC 40.920 ("Standards for delegation of routine duties")	Unclear. (all tasks must be delegated in order for MA's to do them). 12 AAC 40.920. ("Standards for delegation of routine duties.") AS 08.68.850 ("Definitions")	Unclear. (all tasks must be delegated in order for MA's to do them). 12 AAC 40.920. ("Standards for delegation of routine duties.") AS 08.68.850 ("Definitions")	Unclear. (all tasks must be delegated in order for MA's to do them). 12 AAC 40.920. ("Standards for delegation of routine duties.") AS 08.68.850 ("Definitions")	Likely Yes, if task is delegated to MA (all tasks must be delegated in order for MA's to do them). 12 AAC 40.920. ("Standards for delegation of routine duties.") AS 08.68.850 ("Definitions")
Emergency Medical Technician	No. No policy found explicitly including this in scope of practice. [56]	No. No policy found explicitly including this in scope of practice. [56]	No. No policy found explicitly including this in scope of practice. [56]	Yes[56], "intravenous access" is listed under duties. 7 AAC 26.240 ("Scope of certified activities.")	Likely no. Not included in scope of practice.[57]	Likely no. Not included in scope of practice.[57]	Yes. EMT-2 and above[56] 7 AAC 26.240. ("Scope of certified activities.")	Likely, yes. Not stated, but basic care guidelines include knowing blood pressure.[57]
Paramedic	No. No policy found explicitly including this in scope of practice. [56]	No. No policy found explicitly including this in scope of practice. [56]	No. No policy found explicitly including this in scope of practice. [56]	Yes[56], "intravenous access" is listed under duties. 7 AAC 26.240 ("Scope of certified activities.")	Likely no. Not included in scope of practice.[57]	Likely no. Not included in scope of practice.[57]	Yes. 7 AAC 26.890 ("Scope of authorized activities.")	Likely, yes. Not stated, but basic care guidelines include knowing blood pressure.[57]
Community Heath Worker	No. Not included in specicifc scope. [58]	No. Not included in specicifc scope. [58]	No. Not included in specicifc scope. [58]	No. Not included in specicifc scope. [58]	No. Not included in specicifc scope. [58]	No. Not included in specicifc scope. [58]	No. Not included in specicifc scope. [58]	Maybe. If the CHW is a Community Health Aide through the CHAP program. [58]

Coding Framework = Yes, Likely Yes, Maybe, Unclear, Likely No, No

Arizona	Prescribing antihypertensives	Prescribing heart failure medicines	Monitoring INR and modifying warfarin Rx	Administering thrombolytics for STEMI	Conducting point of care ultrasound screening for heart disease	Conducting Ankle Brachial Index Test	Conducting ECG	Conducting blood pressure test
**Nurse Practitioner**	Maybe A.R.S. 32-1601 (23) ("Definitions") , A.A.C. R4-19-508. ("Standards Related to RNP, CNM, and CNS Scope of Practice") Has to prove 45 hours of pharmacology and apply for prescriptive authority per A.A.C. R4-19-511.	Maybe A.R.S. 32-1601 (23) ("Definitions") , A.A.C. R4-19-508. ("Standards Related to RNP, CNM, and CNS Scope of Practice") Has to prove 45 hours of pharmacology and apply for prescriptive authority per A.A.C. R4-19-511.	Maybe A.R.S. 32-1601 (23) ("Definitions") , A.A.C. R4-19-508. ("Standards Related to RNP, CNM, and CNS Scope of Practice") Has to prove 45 hours of pharmacology and apply for prescriptive authority per A.A.C. R4-19-511.	Likely Yes. A.R.S. 32-1601 (23) ("Definitions") , A.A.C. R4-19-508. ("Standards Related to RNP, CNM, and CNS Scope of Practice")	Likely Yes. A.R.S. 32-1601 (23) ("Definitions") , A.A.C. R4-19-508. ("Standards Related to RNP, CNM, and CNS Scope of Practice")	Likely Yes. A.R.S. 32-1601 (23) ("Definitions") , A.A.C. R4-19-508. ("Standards Related to RNP, CNM, and CNS Scope of Practice")	Likely Yes. A.R.S. 32-1601 (23) ("Definitions") , A.A.C. R4-19-508. ("Standards Related to RNP, CNM, and CNS Scope of Practice")	Likely Yes. A.R.S. 32-1601 (23) ("Definitions") , A.A.C. R4-19-508. ("Standards Related to RNP, CNM, and CNS Scope of Practice")
**Registered Nurse**	Likely No. A.R.S. § 32-1601 (24) ("Definitions")	Likely No. A.R.S. § 32-1601 (24) ("Definitions")	Likely No, but maybe can assist someone with prescribing power? [10] A.A.C. R4-19-402 ("Standards Related to Registered Nurse Scope of Practice") "administers prescribed aspects of care including treatments, therapies, and medications" which to me means it might be ok with a written order. A.A.C. R4-19-402. (4a) ("Standards Related to Registered Nurse Scope of Practice")	Yes. RNs can administer drugs. ("Standards Related to Registered Nurse Scope of Practice"). Document from Board of Nursing states that thrombolytics require close RN monitoring which to me means an RN is permitted to administer. [60]	Unclear. Not sure if advisory opinion is saying that ONLY OBGYN ultrasounds are allowed under certain conditions, or if OBYGYN ultrasounds are ONLY allowed under certain conditions and there are no specifics laws re: other ultrasounds. ("Advisory Opinion Ultrasounds: Limited Obstetric, Gynecologic, and Reproductive") [61] A.R.S. 32-1601 (24)	Unclear. "Gathers or reviews comprehensive subjective and objective data" A.A.C. R4-19-402 ("Standards Related to Registered Nurse Scope of Practice")	Unclear. "Gathers or reviews comprehensive subjective and objective data and detects changes or missing information" A.A.C.R4-19-402. ("Standards Related to Registered Nurse Scope of Practice")	Likely Yes. "Gathers or reviews comprehensive subjective and objective data" A.A.C. R4-19-402. ("Standards Related to Registered Nurse Scope of Practice")
**Licensed Practical Nurse**	Likely No. A.A.C. R4-19-401. ("Standards Related to Licensed Practical Nurse Scope of Practice")	Likely No. A.A.C. R4-19-401. ("Standards Related to Licensed Practical Nurse Scope of Practice")	Likely No. A.A.C. R4-19-401. ("Standards Related to Licensed Practical Nurse Scope of Practice")	No. Thrombolytics specifically not under LPN Scope of practice [60]	Unclear, document states that LPNs with advanced training can perform ultrasound to gain peripheral access for peripheral short IV catheter, which suggests that they may be able to use ultrasound in other contexts with advanced training. [60]	Unclear, only reads "contribute to the assessment of the health status of clients by gathering and recording assessment data" A.A.C. R4-19-401 ("Standards Related to Licensed Practical Nurse Scope of Practice")	Unclear, only reads "gathering and recording assessment data." A.A.C. R4-19-401 ("Standards Related to Licensed Practical Nurse Scope of Practice")	Likely yes, under "contribute to the assessment of the health status of clients by gathering and recording assessment data" A.A.C. R4-19-401 ("Standards Related to Licensed Practical Nurse Scope of Practice")
**Physician Assistant**	Maybe. PA with less than 8,000 hours required to have supervision agreement with physician. A.R.S. 32-2531 ("Physician assistant scope of practice; health care tasks; supervision agreements; supervising physician duties; civil penalty")	Maybe. A.R.S. 32-2531 ("Physician assistant scope of practice; health care tasks; supervision agreements; supervising physician duties; civil penalty")	Maybe. A.R.S. 32-2531 ("Physician assistant scope of practice; health care tasks; supervision agreements; supervising physician duties; civil penalty")	Maybe. A.R.S. 32-2531 ("Physician assistant scope of practice; health care tasks; supervision agreements; supervising physician duties; civil penalty")	Maybe. A.R.S. 32-2531 ("Physician assistant scope of practice; health care tasks; supervision agreements; supervising physician duties; civil penalty")	Maybe. A.R.S. 32-2531 ("Physician assistant scope of practice; health care tasks; supervision agreements; supervising physician duties; civil penalty")	Maybe. A.R.S. 32-2531 ("Physician assistant scope of practice; health care tasks; supervision agreements; supervising physician duties; civil penalty")	Maybe. A.R.S. 32-2531 ("Physician assistant scope of practice; health care tasks; supervision agreements; supervising physician duties; civil penalty")
**Pharmacist**	Maybe. Collaborative practice agreement: "initiate, monitor, or modify drug therapy or assist in disease management assistance" A.R.S. § 32-1970. ("Collaborative practice agreements; requirements; rules; definitions")	Maybe. Collaborative practice agreement: "initiate, monitor, or modify drug therapy or assist in disease management assistance" A.R.S. § 32-1970. ("Collaborative practice agreements; requirements; rules; definitions")	Maybe. Collaborative practice agreement: "initiate, monitor, or modify drug therapy or assist in disease management assistance" A.R.S. § 32-1970. ("Collaborative practice agreements; requirements; rules; definitions")	Maybe. Collaborative practice agreement: "initiate, monitor, or modify drug therapy or assist in disease management assistance" A.R.S. § 32-1970. ("Collaborative practice agreements; requirements; rules; definitions")	Maybe. Collaborative practice agreement: "initiate, monitor, or modify drug therapy or assist in disease management assistance" A.R.S. § 32-1970. ("Collaborative practice agreements; requirements; rules; definitions")	Maybe. Collaborative practice agreement: "initiate, monitor, or modify drug therapy or assist in disease management assistance" A.R.S. § 32-1970. ("Collaborative practice agreements; requirements; rules; definitions")	Maybe. Collaborative practice agreement: "initiate, monitor, or modify drug therapy or assist in disease management assistance" A.R.S. § 32-1970. ("Collaborative practice agreements; requirements; rules; definitions")	Maybe. Collaborative practice agreement: "initiate, monitor, or modify drug therapy or assist in disease management assistance" A.R.S. § 32-1970. ("Collaborative practice agreements; requirements; rules; definitions")
**Medical Assistant**	No. "Assists in medical practice under the supervision of [doctor, PA, NP] and performs delegated procedures commensurate with the MAs … training but does not diagnose, interpret, design, or modify established treatment programs or perform any functions that would violate any statute applicable to the practice of medicine." A.R.S. 32-1401 (16). ("Definitions")	No. "Assists in medical practice under the supervision of [doctor, PA, NP] and performs delegated procedures commensurate with the MAs … training but does not diagnose, interpret, design, or modify established treatment programs or perform any functions that would violate any statute applicable to the practice of medicine." A.R.S. 32-1401 (16). ("Definitions")	No. "Assists in medical practice under the supervision of [doctor, PA, NP] and performs delegated procedures commensurate with the MAs … training but does not diagnose, interpret, design, or modify established treatment programs or perform any functions that would violate any statute applicable to the practice of medicine." A.R.S. 32-1401 (16). ("Definitions")	No. if IV because under delegation law , MA's can be delegated tasks that: "Apply pharmacology principles to prepare and administer oral, inhalant, topical, otic, optic, rectal, vaginal and parenteral medications (excluding intravenous medications)" A.A.C. R4-19-509. ("Delegation to Medical Assistants")	Unclear. "Assists in medical practice under the supervision of [doctor, PA, NP] and performs delegated procedures commensurate with the MAs … training but does not diagnose, interpret, design, or modify established treatment programs or perform any functions that would violate any statute applicable to the practice of medicine." A.R.S. 32-1401 (16). ("Definitions") Ultrasound therapy permitted (reads differently than diagnostic ultrasound) A.A.C. R4-22-402. ("Medical Assistants - Authorized Procedures")	Unclear. ABI not listed in authorized procedures. A.A.C. R4-22-402. ("Medical Assistants - Authorized Procedures")	Maybe. Perform EKG listed under authorized procedure. A.A.C. R4-22-402. ("Medical Assistants - Authorized Procedures")	Likely Yes. If we consider blood pressure a vital sign. A.A.C. R4-22-402. ("Medical Assistants - Authorized Procedures")
**Emergency Medical Technician**	No. No policy found explicitly including this in scope of practice.	No. No policy found explicitly including this in scope of practice.	No. No policy found explicitly including this in scope of practice.	No. [59]	Unclear. A.A.C. R9-25-202. ("On-line Medical Direction") [59]	Likely no. A.A.C. R9-25-202. ("On-line Medical Direction") [59]	Maybe. If an EMT-intermediate. Cardiac monitoring - 12-lead ECG acquisition and transmission. Any level of EMT can do acquisition and transmission, only EMT-I can interpret. [59]	Yes. A.A.C. R9-25-202. ("On-line Medical Direction") [59]

**Paramedic**	No. No policy found explicitly including this in scope of practice.	No. No policy found explicitly including this in scope of practice.	No. No policy found explicitly including this in scope of practice.	Maybe with additional training. A.A.C. R9-25-202. ("On-line Medical Direction") [59]	Unclear. A.A.C. R9-25-202. ("On-line Medical Direction") [59]	Likely no. A.A.C. R9-25-202. ("On-line Medical Direction") [59]	Yes. A.A.C. R9-25-202. ("On-line Medical Direction") [59]	Yes. A.A.C. R9-25-202. ("On-line Medical Direction") [59]
**Community Heath Worker**	No. "A certified CHW shall not provide physical health services or behavioral health services to a client." A.A.C. R9-16-802. ("Community Health Workers Eligibility and Scope of Practice")	No. "A certified CHW shall not provide physical health services or behavioral health services to a client." A.A.C. R9-16-802. ("Community Health Workers Eligibility and Scope of Practice")	No. "A certified CHW shall not provide physical health services or behavioral health services to a client." A.A.C. R9-16-802. ("Community Health Workers Eligibility and Scope of Practice")	No. "A certified CHW shall not provide physical health services or behavioral health services to a client." A.A.C. R9-16-802. ("Community Health Workers Eligibility and Scope of Practice")	No. "A certified CHW shall not provide physical health services or behavioral health services to a client." A.A.C. R9-16-802. ("Community Health Workers Eligibility and Scope of Practice")	No. "A certified CHW shall not provide physical health services or behavioral health services to a client." A.A.C. R9-16-802. ("Community Health Workers Eligibility and Scope of Practice")	No. "A certified CHW shall not provide physical health services or behavioral health services to a client." A.A.C. R9-16-802. ("Community Health Workers Eligibility and Scope of Practice")	No. "A certified CHW shall not provide physical health services or behavioral health services to a client." A.A.C. R9-16-802. ("Community Health Workers Eligibility and Scope of Practice")

Coding Framework = Yes, Likely Yes, Maybe, Unclear, Likely No, No

Indian Health Service	Prescribing antihypertensives	Prescribing heart failure medicines	Monitoring INR and modifying warfarin Rx	Conducting point of care ultrasound screening for heart disease	Administering thrombolytics for STEMI	Conducting Ankle Brachial Index Test	Conducting ECG	Conducting blood pressure test
**Nurse Practitioner**	Likely yes - "Prescribes and dispenses medications and durable medical devices and supplies" [62]	Likely yes - "Prescribes and dispenses medications and durable medical devices and supplies" [62]	Likely yes - "Prescribes and dispenses medications and durable medical devices and supplies" [62]	Likely yes - "orders, conducts, and interprets screening studies, tests, and diagnostic procedures to assess and diagnose problems and establish management/treatment plans." [62]	Likely yes - "Prescribes and dispenses medications and durable medical devices and supplies" [62] [note - assuming adminsitration because they have prescribing authority)	Likely yes - "orders, conducts, and interprets screening studies, tests, and diagnostic procedures to assess and diagnose problems and establish management/treatment plans." [62]	Likely yes - "orders, conducts, and interprets screening studies, tests, and diagnostic procedures to assess and diagnose problems and establish management/treatment plans." [62]	Likely yes - "orders, conducts, and interprets screening studies, tests, and diagnostic procedures to assess and diagnose problems and establish management/treatment plans." [62]
**Registered Nurse**	Likely no. Definition of professional nursing includes "assessment, diagnosis, planning, intervention, and evaluation of nursing care." [62]	Likely no. Definition of professional nursing includes "assessment, diagnosis, planning, intervention, and evaluation of nursing care." [62]	Likely no. Definition of professional nursing includes "assessment, diagnosis, planning, intervention, and evaluation of nursing care." [62]	Unclear. Definition of professional nursing includes "assessment, diagnosis, planning, intervention, and evaluation of nursing care." [62]	Unclear. Definition of professional nursing includes "assessment, diagnosis, planning, intervention, and evaluation of nursing care." [62]	Unclear. Definition of professional nursing includes "assessment, diagnosis, planning, intervention, and evaluation of nursing care." [62]	Unclear. Definition of professional nursing includes "assessment, diagnosis, planning, intervention, and evaluation of nursing care." [62]	Unclear. Definition of professional nursing includes "assessment, diagnosis, planning, intervention, and evaluation of nursing care." [62]
**Licensed Practical Nurse**	Likely no. Definition of practical nursing includes "performance of skilled nursing services under the supervision of a registered nurse" [62]	Likely no. Definition of practical nursing includes "performance of skilled nursing services under the supervision of a registered nurse" [62]	Likely no. Definition of practical nursing includes "performance of skilled nursing services under the supervision of a registered nurse" [62]	Unclear. Likely no. Definition of practical nursing includes "performance of skilled nursing services under the supervision of a registered nurse" [62]	Unclear. Likely no. Definition of practical nursing includes "performance of skilled nursing services under the supervision of a registered nurse" [62]	Unclear. Definition of practical nursing includes "performance of skilled nursing services under the supervision of a registered nurse" [62]	Unclear. Likely no. Definition of practical nursing includes "performance of skilled nursing services under the supervision of a registered nurse" [62]	Unclear. Likely no. Definition of practical nursing includes "performance of skilled nursing services under the supervision of a registered nurse" [62]
**Physician Assistant**	Maybe - "Prescribe and dispense medications and durable medical devices and supplies" … "The PA's clinical privileges shall be commensurate with their education, experiences, competencies, and operational needs for the service to which they are assigned." Chapter 28. Physician Assistants [63]	Maybe - "Prescribe and dispense medications and durable medical devices and supplies" … "The PA's clinical privileges shall be commensurate with their education, experiences, competencies, and operational needs for the service to which they are assigned." Chapter 28. Physician Assistants [63]	Maybe - "Prescribe and dispense medications and durable medical devices and supplies" … "The PA's clinical privileges shall be commensurate with their education, experiences, competencies, and operational needs for the service to which they are assigned." Chapter 28. Physician Assistants [63]	Maybe - "order and interpret diagnostic studies such as laboratory tests, radiological exams, electrocardiograms, or other studies as appropriate and specified in each PA's clinical privileges" Chapter 28. Physician Assistants [63]	Maybe - "prescribing privileges includes administering pharmaceuticals, where appropriate to do so. Chapter 28. Physician Assistants [63]	Maybe - "order and interpret diagnostic studies such as laboratory tests, radiological exams, electrocardiograms, or other studies as appropriate and specified in each PA's clinical privileges" Chapter 28. Physician Assistants [63]	Maybe - "order and interpret diagnostic studies such as laboratory tests, radiological exams, electrocardiograms, or other studies as appropriate and specified in each PA's clinical privileges" Chapter 28. Physician Assistants [63]	Likely yes - perform initial and/or periodic histories and physical examinations Chapter 28. Physician Assistants [63]
**Pharmacist**	Maybe - if included in collaborative practice agreement. [64]	Maybe - if included in collaborative practice agreement. [64]	Maybe - if included in collaborative practice agreement. [64]	Maybe - if included in collaborative practice agreement. [64]	Maybe - if included in collaborative practice agreement. [64]	Maybe - if included in collaborative practice agreement. [64]	Maybe - if included in collaborative practice agreement. [64]	Maybe - if included in collaborative practice agreement. [64]
**Medical Assistant**	Could not find an IHS scope of practice.	Could not find an IHS scope of practice.	Could not find an IHS scope of practice.	Could not find an IHS scope of practice.	Could not find an IHS scope of practice.	Could not find an IHS scope of practice.	Could not find an IHS scope of practice.	Could not find an IHS scope of practice.
**Emergency Medical Technician**	Unclear. EMS is housed in the Division of Clinical & Community Services, but could not find any scope of practice or EMS protocols. Per website, "National Medical Direction and National EMS training functions are carried out by staff assigned to field locations." [65]	Unclear. EMS is housed in the Division of Clinical & Community Services, but could not find any scope of practice or EMS protocols. Per website, "National Medical Direction and National EMS training functions are carried out by staff assigned to field locations." [65]	Unclear. EMS is housed in the Division of Clinical & Community Services, but could not find any scope of practice or EMS protocols. Per website, "National Medical Direction and National EMS training functions are carried out by staff assigned to field locations." [65]	Unclear. EMS is housed in the Division of Clinical & Community Services, but could not find any scope of practice or EMS protocols. Per website, "National Medical Direction and National EMS training functions are carried out by staff assigned to field locations." [65]	Unclear. EMS is housed in the Division of Clinical & Community Services, but could not find any scope of practice or EMS protocols. Per website, "National Medical Direction and National EMS training functions are carried out by staff assigned to field locations." [65]	Unclear. EMS is housed in the Division of Clinical & Community Services, but could not find any scope of practice or EMS protocols. Per website, "National Medical Direction and National EMS training functions are carried out by staff assigned to field locations." [65]	Unclear. EMS is housed in the Division of Clinical & Community Services, but could not find any scope of practice or EMS protocols. Per website, "National Medical Direction and National EMS training functions are carried out by staff assigned to field locations." [65]	Unclear. EMS is housed in the Division of Clinical & Community Services, but could not find any scope of practice or EMS protocols. Per website, "National Medical Direction and National EMS training functions are carried out by staff assigned to field locations." [65]
**Paramedic**	Unclear. EMS is housed in the Division of Clinical & Community Services, but could not find any scope of practice or EMS protocols. Per website, "National Medical Direction and National EMS training functions are carried out by staff assigned to field locations." [65]	Unclear. EMS is housed in the Division of Clinical & Community Services, but could not find any scope of practice or EMS protocols. Per website, "National Medical Direction and National EMS training functions are carried out by staff assigned to field locations." [65]	Unclear. EMS is housed in the Division of Clinical & Community Services, but could not find any scope of practice or EMS protocols. Per website, "National Medical Direction and National EMS training functions are carried out by staff assigned to field locations." [65]	Unclear. EMS is housed in the Division of Clinical & Community Services, but could not find any scope of practice or EMS protocols. Per website, "National Medical Direction and National EMS training functions are carried out by staff assigned to field locations." [65]	Unclear. EMS is housed in the Division of Clinical & Community Services, but could not find any scope of practice or EMS protocols. Per website, "National Medical Direction and National EMS training functions are carried out by staff assigned to field locations." [65]	Unclear. EMS is housed in the Division of Clinical & Community Services, but could not find any scope of practice or EMS protocols. Per website, "National Medical Direction and National EMS training functions are carried out by staff assigned to field locations." [65]	Unclear. EMS is housed in the Division of Clinical & Community Services, but could not find any scope of practice or EMS protocols. Per website, "National Medical Direction and National EMS training functions are carried out by staff assigned to field locations." [65]	Unclear. EMS is housed in the Division of Clinical & Community Services, but could not find any scope of practice or EMS protocols. Per website, "National Medical Direction and National EMS training functions are carried out by staff assigned to field locations." [65]

**Community Heath Worker**	Likely no. Community Health Representative. "They assist by increasing health knowledge of patients and communities … transportation, outreach, community education, informal counseling, social support, and advocacy." [66]	Likely no. Community Health Representative. "They assist by increasing health knowledge of patients and communities … transportation, outreach, community education, informal counseling, social support, and advocacy." [66]	Likely no. Community Health Representative. "They assist by increasing health knowledge of patients and communities … transportation, outreach, community education, informal counseling, social support, and advocacy." [66]	Likely no. Community Health Representative. "They assist by increasing health knowledge of patients and communities … transportation, outreach, community education, informal counseling, social support, and advocacy." [66]	Likely no. Community Health Representative. "They assist by increasing health knowledge of patients and communities … transportation, outreach, community education, informal counseling, social support, and advocacy." [66]	Likely no. Community Health Representative. "They assist by increasing health knowledge of patients and communities … transportation, outreach, community education, informal counseling, social support, and advocacy." [66]	Likely no. Community Health Representative. "They assist by increasing health knowledge of patients and communities … transportation, outreach, community education, informal counseling, social support, and advocacy." [66]	Likely no. Community Health Representative. "They assist by increasing health knowledge of patients and communities … transportation, outreach, community education, informal counseling, social support, and advocacy." [66]

Coding Framework = Yes, Likely Yes, Maybe, Unclear, Likely No, No

California	Prescribing antihypertensives	Prescribing heart failure medicines	Monitoring INR and modifying warfarin Rx	Administering thrombolytics for STEMI	Conducting point of care ultrasound screening for heart disease	Conducting Ankle Brachial Index Test	Conducting ECG	Conducting blood pressure test
**Nurse Practitioner**	Maybe. There are three categories of NP: NP, 103 NP, and 104 NP. A "regular" NP must practice under standardized procedure functions (16 CCR § 1472) and "103 NP" can practice in a group setting with at least one physician and surgeon within the population focus of their national certification and "104 NP" may work independently within the population focus of their national certification. 3 years as a 103 NP is required before applying to be a 104 NP. CCR 16 § 1481. ("Categories and Scope of Practice of Nurse Practitioners.") In CA NPs can only practice in the area of their national certification. See also "An explanation of standardized procedure requirements for nurse practitioner practice (5) Statue on NP furnishing drugs (West's Ann.Cal.Bus. & Prof.Code § 2836.1)	Maybe. There are three categories of NP: NP, 103 NP, and 104 NP. A "regular" NP must practice under standardized procedure functions (16 CCR § 1472) and "103 NP" can practice in a group setting with at least one physician and surgeon within the population focus of their national certification and "104 NP" may work independently within the population focus of their national certification. 3 years as a 103 NP is required before applying to be a 104 NP. CCR 16 § 1481. ("Categories and Scope of Practice of Nurse Practitioners.") In CA NPs can only practice in the area of their national certification. See also "An explanation of standardized procedure requirements for nurse practitioner practice (5) Statue on NP furnishing drugs (West's Ann.Cal.Bus. & Prof.Code § 2836.1)	Maybe. There are three categories of NP: NP, 103 NP, and 104 NP. A "regular" NP must practice under standardized procedure functions (16 CCR § 1472) and "103 NP" can practice in a group setting with at least one physician and surgeon within the population focus of their national certification and "104 NP" may work independently within the population focus of their national certification. 3 years as a 103 NP is required before applying to be a 104 NP. CCR 16 § 1481. ("Categories and Scope of Practice of Nurse Practitioners.") In CA NPs can only practice in the area of their national certification. See also "An explanation of standardized procedure requirements for nurse practitioner practice (5) Statue on NP furnishing drugs (West's Ann.Cal.Bus. & Prof.Code § 2836.1)	Likely Yes. There are three NP categories of NP: NP, 103 NP, and 104 NP. A "regular" NP must practice under standardized procedure functions (16 CCR § 1472) and "103 NP" can practice in a group setting with at least one physician and surgeon within the population focus of their national certification and "104 NP" may work independently within the population focus of their national certification. 3 years as a 103 NP is required before applying to be a 104 NP. CCR 16 § 1481 ("Categories and Scope of Practice of Nurse Practitioners."). In CA NPs can only practice in the area of their national certification. See also "An explanation of standardized procedure requirements for nurse practitioner practice (5)	Maybe. There are three NP categories of NP: NP, 103 NP, and 104 NP. A "regular" NP must practice under standardized procedure functions (16 CCR § 1472) and "103 NP" can practice in a group setting with at least one physician and surgeon within the population focus of their national certification and "104 NP" may work independently within the population focus of their national certification. 3 years as a 103 NP is required before applying to be a 104 NP. CCR 16 § 1481 ("Categories and Scope of Practice of Nurse Practitioners."). In CA NPs can only practice in the area of their national certification. See also "An explanation of standardized procedure requirements for nurse practitioner practice (5)	Maybe. There are three NP categories of NP: NP, 103 NP, and 104 NP. A "regular" NP must practice under standardized procedure functions (16 CCR § 1472) and "103 NP" can practice in a group setting with at least one physician and surgeon within the population focus of their national certification and "104 NP" may work independently within the population focus of their national certification. 3 years as a 103 NP is required before applying to be a 104 NP. CCR 16 § 1481 ("Categories and Scope of Practice of Nurse Practitioners."). In CA NPs can only practice in the area of their national certification. See also "An explanation of standardized procedure requirements for nurse practitioner practice (5)	Maybe. There are three NP categories of NP: NP, 103 NP, and 104 NP. A "regular" NP must practice under standardized procedure functions (16 CCR § 1472) and "103 NP" can practice in a group setting with at least one physician and surgeon within the population focus of their national certification and "104 NP" may work independently within the population focus of their national certification. 3 years as a 103 NP is required before applying to be a 104 NP. CCR 16 § 1481 ("Categories and Scope of Practice of Nurse Practitioners."). In CA NPs can only practice in the area of their national certification. See also "An explanation of standardized procedure requirements for nurse practitioner practice (5)	Likely Yes. There are three NP categories of NP: NP, 103 NP, and 104 NP. A "regular" NP must practice under standardized procedure functions (16 CCR § 1472) and "103 NP" can practice in a group setting with at least one physician and surgeon within the population focus of their national certification and "104 NP" may work independently within the population focus of their national certification. 3 years as a 103 NP is required before applying to be a 104 NP. CCR 16 § 1481 ("Categories and Scope of Practice of Nurse Practitioners."). In CA NPs can only practice in the area of their national certification. See also "An explanation of standardized procedure requirements for nurse practitioner practice (5)
**Registered Nurse**	Likely No. CA BUS & PROF § 2725. ("Legislative declaration; practice of nursing; functions")	Likely No. CA BUS & PROF § 2725. ("Legislative declaration; practice of nursing; functions")	Likely No. CA BUS & PROF § 2725. ("Legislative declaration; practice of nursing; functions")	Likely yes. CA BUS & PROF § 2725. ("Legislative declaration; practice of nursing; functions")	Unclear. CA BUS & PROF § 2725. ("Legislative declaration; practice of nursing; functions"	Unclear. CA BUS & PROF § 2725. ("Legislative declaration; practice of nursing; functions"	Unclear. CA BUS & PROF § 2725. ("Legislative declaration; practice of nursing; functions"	Likely yes. CA BUS & PROF § 2725. ("Legislative declaration; practice of nursing; functions")
**Licensed Practical Nurse***	Likely No. 16 CCR § 2518.5. ("Scope of Vocational Nursing Practice")	Likely No. 16 CCR § 2518.5. ("Scope of Vocational Nursing Practice")	Likely No. 16 CCR § 2518.5. ("Scope of Vocational Nursing Practice")	Maybe. a LVN must be certified by the Board to perform intravenous therapy. 16 CCR § 2518.5. ("Scope of Vocational Nursing Practice")	Unclear. "...uses and practices basic assessment (data collection), participates in planning, executes interventions in accordance with care plan or treatment plan..." 16 CCR § 2518.5. ("Scope of Vocational Nursing Practice")	Unclear. "...uses and practices basic assessment (data collection), participates in planning, executes interventions in accordance with care plan or treatment plan..." 16 CCR § 2518.5. ("Scope of Vocational Nursing Practice")	Unclear. "...uses and practices basic assessment (data collection), participates in planning, executes interventions in accordance with care plan or treatment plan..." 16 CCR § 2518.5. ("Scope of Vocational Nursing Practice")	Likely yes. "...uses and practices basic assessment (data collection), participates in planning, executes interventions in accordance with care plan or treatment plan..." 16 CCR § 2518.5. ("Scope of Vocational Nursing Practice")
**Physician Assistant**	Maybe. PA can furnish and order a drug in accordance with their practice agreement. CA BUS & PROF CODE 3502.1. ("Furnishing or ordering drugs or devices; practice agreement; requirements and supervision by physician and surgeon; furnishing Schedule II through Schedule V controlled substances; educational requirements")	Maybe. PA can furnish and order a drug in accordance with their practice agreement. CA BUS & PROF CODE 3502.1. ("Furnishing or ordering drugs or devices; practice agreement; requirements and supervision by physician and surgeon; furnishing Schedule II through Schedule V controlled substances; educational requirements")	Maybe. PA can furnish and order a drug in accordance with their practice agreement. CA BUS & PROF CODE 3502.1. ("Furnishing or ordering drugs or devices; practice agreement; requirements and supervision by physician and surgeon; furnishing Schedule II through Schedule V controlled substances; educational requirements")	Maybe. Can practice under a practice agreement with a physician. CA BUS & PROF 3502.3 ("Practice agreements; contents; authorized activities") "Order, transmit an order for, perform, or assist in the performance of laboratory procedures, screening procedures and therapeutic procedures." 16 CCR § 1399.541. ("Medical Services Performable.")	Maybe. Can practice under a practice agreement with a physician. CA BUS & PROF 3502.3 ("Practice agreements; contents; authorized activities") "Order, transmit an order for, perform, or assist in the performance of laboratory procedures, screening procedures and therapeutic procedures." 16 CCR § 1399.541. ("Medical Services Performable.")	Maybe. Can practice under a practice agreement with a physician. CA BUS & PROF 3502.3 ("Practice agreements; contents; authorized activities") "Order, transmit an order for, perform, or assist in the performance of laboratory procedures, screening procedures and therapeutic procedures." 16 CCR § 1399.541. ("Medical Services Performable.")	Maybe. Can practice under a practice agreement with a physician. CA BUS & PROF 3502.3 ("Practice agreements; contents; authorized activities") "Order, transmit an order for, perform, or assist in the performance of laboratory procedures, screening procedures and therapeutic procedures." 16 CCR § 1399.541. ("Medical Services Performable.")	Maybe. Can practice under a practice agreement with a physician. CA BUS & PROF 3502.3 ("Practice agreements; contents; authorized activities") "Order, transmit an order for, perform, or assist in the performance of laboratory procedures, screening procedures and therapeutic procedures." 16 CCR § 1399.541. ("Medical Services Performable.")
**Pharmacist**	Maybe. If under a collaborative practice agreement. CA BUS & PROF § 4052 ("Power to perform procedures and functions")	Maybe. If under a collaborative practice agreement. CA BUS & PROF § 4052 ("Power to perform procedures and functions")	Maybe "Initiating or adjusting the drug regimen of a patient pursuant to an order or authorization made by the patient's prescriber and in accordance with the policies, procedures, or protocols of the licensed health care facility." CA BUS & PROF § 4052.1. ("Authorized procedures performed in a health care facility")	Maybe. If under a collaborative practice agreement. CA BUS & PROF § 4052 ("Power to perform procedures and functions")	Maybe. If under a collaborative practice agreement. CA BUS & PROF § 4052 ("Power to perform procedures and functions")	Maybe. If under a collaborative practice agreement. CA BUS & PROF § 4052 ("Power to perform procedures and functions")	Maybe. If under a collaborative practice agreement. CA BUS & PROF § 4052 ("Power to perform procedures and functions")	Maybe. If under a collaborative practice agreement. CA BUS & PROF § 4052 ("Power to perform procedures and functions")

**Medical Assistant**	No. "simple routine medical tasks and procedures that may be safely performed by a medical assistant who has limited training" CA BUS & PROF § 2069 ("Medical assistants; authorized tasks under direction of certain medical professionals; delegation of supervision; authorization of activities which medical assistant is not authorized to perform prohibited; inpatient care in general acute care hospitals prohibited")	No. "simple routine medical tasks and procedures that may be safely performed by a medical assistant who has limited training" CA BUS & PROF § 2069 ("Medical assistants; authorized tasks under direction of certain medical professionals; delegation of supervision; authorization of activities which medical assistant is not authorized to perform prohibited; inpatient care in general acute care hospitals prohibited")	No. "simple routine medical tasks and procedures that may be safely performed by a medical assistant who has limited training" CA BUS & PROF § 2069 ("Medical assistants; authorized tasks under direction of certain medical professionals; delegation of supervision; authorization of activities which medical assistant is not authorized to perform prohibited; inpatient care in general acute care hospitals prohibited")	No. "MA may adminster medication only by intradermal, subcutaneous, or intramuscular injections..." CA BUS & PROF § 2069 ("Medical assistants; authorized tasks under direction of certain medical professionals; delegation of supervision; authorization of activities which medical assistant is not authorized to perform prohibited; inpatient care in general acute care hospitals prohibited")	Unclear. physician, NP, PA, CNM, may provide written instructions to be followed by MA. "simple routine medical tasks and procedures that may be safely performed by a medical assistant who has limited training" CA BUS & PROF § 2069 ("Medical assistants; authorized tasks under direction of certain medical professionals; delegation of supervision; authorization of activities which medical assistant is not authorized to perform prohibited; inpatient care in general acute care hospitals prohibited")	Unclear. physician, NP, PA, CNM, may provide written instructions to be followed by MA. "simple routine medical tasks and procedures that may be safely performed by a medical assistant who has limited training" CA BUS & PROF § 2069 ("Medical assistants; authorized tasks under direction of certain medical professionals; delegation of supervision; authorization of activities which medical assistant is not authorized to perform prohibited; inpatient care in general acute care hospitals prohibited")	Likely yes. physician, NP, PA, CNM, may provide written instructions to be followed by MA. "simple routine medical tasks and procedures that may be safely performed by a medical assistant who has limited training" CA BUS & PROF § 2069 ("Medical assistants; authorized tasks under direction of certain medical professionals; delegation of supervision; authorization of activities which medical assistant is not authorized to perform prohibited; inpatient care in general acute care hospitals prohibited")	Unclear. physician, NP, PA, CNM, may provide written instructions to be followed by MA. "simple routine medical tasks and procedures that may be safely performed by a medical assistant who has limited training" CA BUS & PROF § 2069 ("Medical assistants; authorized tasks under direction of certain medical professionals; delegation of supervision; authorization of activities which medical assistant is not authorized to perform prohibited; inpatient care in general acute care hospitals prohibited")
**Emergency Medical Technician**	No. 22 CCR s 100106. ("Advanced EMT Scope of Practice.")	No. 22 CCR s 100106. ("Advanced EMT Scope of Practice.")	No. 22 CCR s 100106. ("Advanced EMT Scope of Practice.")	No. 22 CCR s 100106. ("Advanced EMT Scope of Practice.")	Likely No. ultrasound not listed in scope of practice of EMT. 22 CCR § 100063 ("Basic Scope of Practice of Emergency Medical Technician") or AEMT 22 CCR s 100106. ("Advanced EMT Scope of Practice.")	Likely no. 22 CCR § 100063. ("Basic Scope of Practice of Emergency Medical Technician.") 22 CCR s 100106. ("Advanced EMT Scope of Practice.")	Likely No. 22 CCR § 100063 ("Basic Scope of Practice of Emergency Medical Technician") 22 CCR s 100106. ("Advanced EMT Scope of Practice.")	Yes. 22 CCR § 100063. ("Basic Scope of Practice of Emergency Medical Technician.")
**Paramedic**	No. 22 CCR § 100146. ("Scope of Practice of Paramedic.")	No. 22 CCR § 100146. ("Scope of Practice of Paramedic.")	No. 22 CCR § 100146. ("Scope of Practice of Paramedic.")	Yes. 22 CCR § 100146. ("Scope of Practice of Paramedic.")	Likely No. 22 CCR § 100146. ("Scope of Practice of Paramedic.")	Likely no. 2 CCR § 100146. ("Scope of Practice of Paramedic.") and 22 CCR § 100063. ("Basic Scope of Practice of Emergency Medical Technician.")	Yes. 22 CCR § 100146. ("Scope of Practice of Paramedic.")	Yes. 22 CCR § 100146. ("Scope of Practice of Paramedic.") says scope also encompasses Advanced EMT or basic EMT scopes and basic scope of practice includes blood pressure. 22 CCR § 100063. ("Basic Scope of Practice of Emergency Medical Technician.")
**Community Heath Worker**	No. Cannot locate scope of practice.	No. Cannot locate scope of practice.	No. Cannot locate scope of practice.	No. Cannot locate scope of practice.	No. Cannot locate scope of practice.	No. Cannot locate scope of practice.	No. Cannot locate scope of practice.	No. Cannot locate scope of practice.

Coding Framework = Yes, Likely Yes, Maybe, Unclear, Likely No, No

* LVN (licensed vocational nurse) in CA

Colorado	Prescribing antihypertensives	Prescribing heart failure medicines	Monitoring INR and modifying warfarin Rx	Administering thrombolytics for STEMI	Conducting point of care ultrasound screening for heart disease	Conducting Ankle Brachial Index Test	Conducting ECG	Conducting blood pressure test
**Nurse Practitioner**	Maybe. "Provisional Prescriptive Authority" (Full Prescriptive Authority requirements must be fulfilled in addition) 3 CCR 716-1:1.15-B; Section 12-255-112(4), C.R.S. ("Rules and Regulations for Prescriptive Authority for Advanced Practice Registered Nurses") Must work for 3 years as a professional nurse or APRN, then can obtain provisional prescriptive authority (RXN-P), and within three years of receiving RXN-P must complete 750 mentorship with physician or full prescriptive authority APRN. If requirements not met, provisional prescriptive authority expires after 3 years. See also C.R.S.A. 12-255-112.	Maybe. "Provisional Prescriptive Authority" (Full Prescriptive Authority requirements must be fulfilled in addition) 3 CCR 716-1:1.15-B; Section 12-255-112(4), C.R.S. ("Rules and Regulations for Prescriptive Authority for Advanced Practice Registered Nurses") Must work for 3 years as a professional nurse or APRN, then can obtain provisional prescriptive authority (RXN-P), and within three years of receiving RXN-P must complete 750 mentorship with physician or full prescriptive authority APRN. If requirements not met, provisional prescriptive authority expires after 3 years. See also C.R.S.A. 12-255-112.	Maybe. "Provisional Prescriptive Authority" (Full Prescriptive Authority requirements must be fulfilled in addition) 3 CCR 716-1:1.15-B; Section 12-255-112(4), C.R.S. ("Rules and Regulations for Prescriptive Authority for Advanced Practice Registered Nurses") Must work for 3 years as a professional nurse or APRN, then can obtain provisional prescriptive authority (RXN-P), and within three years of receiving RXN-P must complete 750 mentorship with physician or full prescriptive authority APRN. If requirements not met, provisional prescriptive authority expires after 3 years. See also C.R.S.A. 12-255-112.	Likely Yes. "The scope of Advanced Practice Registered Nursing may include, but is not limited to: performing acts of advanced assessment, diagnosing, treating, prescribing, ordering, selecting, administering, and dispensing diagnostic and therapeutic measures." 3 CCR 716-1:1.14-F. ("SCOPE OF ADVANCED PRACTICE REGISTERED NURSING")	Likely Yes. "The scope of Advanced Practice Registered Nursing may include, but is not limited to: performing acts of advanced assessment, diagnosing, treating, prescribing, ordering, selecting, administering, and dispensing diagnostic and therapeutic measures." 3 CCR 716-1:1.14-F. ("SCOPE OF ADVANCED PRACTICE REGISTERED NURSING")	Likely Yes. "The scope of Advanced Practice Registered Nursing may include, but is not limited to: performing acts of advanced assessment, diagnosing, treating, prescribing, ordering, selecting, administering, and dispensing diagnostic and therapeutic measures." 3 CCR 716-1:1.14-F. ("SCOPE OF ADVANCED PRACTICE REGISTERED NURSING")	Likely Yes. "The scope of Advanced Practice Registered Nursing may include, but is not limited to: performing acts of advanced assessment, diagnosing, treating, prescribing, ordering, selecting, administering, and dispensing diagnostic and therapeutic measures." 3 CCR 716-1:1.14-F. ("SCOPE OF ADVANCED PRACTICE REGISTERED NURSING")	Likely Yes. "The scope of Advanced Practice Registered Nursing may include, but is not limited to: performing acts of advanced assessment, diagnosing, treating, prescribing, ordering, selecting, administering, and dispensing diagnostic and therapeutic measures." 3 CCR 716-1:1.14-F. ("SCOPE OF ADVANCED PRACTICE REGISTERED NURSING")
**Registered Nurse**	Likely No. Only reference to scope: "14.5 Nursing staff shall conduct initial and ongoing assessments and screenings of the patient's physical, cognitive, behavioral, emotional, and psychosocial status in sufficient scope and detail to meet the needs of the patient, according to hospital policy and professional standards of practice." 6 CCR 1011-1:4-14. ("NURSING SERVICES")	Likely No. Only reference to scope: "14.5 Nursing staff shall conduct initial and ongoing assessments and screenings of the patient's physical, cognitive, behavioral, emotional, and psychosocial status in sufficient scope and detail to meet the needs of the patient, according to hospital policy and professional standards of practice." 6 CCR 1011-1:4-14. ("NURSING SERVICES")	Likely No. Only reference to scope: "14.5 Nursing staff shall conduct initial and ongoing assessments and screenings of the patient's physical, cognitive, behavioral, emotional, and psychosocial status in sufficient scope and detail to meet the needs of the patient, according to hospital policy and professional standards of practice." 6 CCR 1011-1:4-14. ("NURSING SERVICES")	Unclear. "Providing therapy and treatment that is supportive......" CRSA 12-255-104. ("Definitions")	Unclear. "evaluating health status through the collection and assessment of health data..." CRSA 12-255-104. ("Definitions")	Unclear. "evaluating health status through the collection and assessment of health data..." CRSA 12-255-104. ("Definitions")	Unclear. "evaluating health status through the collection and assessment of health data..." CRSA 12-255-104. ("Definitions")	Likely yes. "evaluating health status through the collection and assessment of health data..." CRSA 12-255-104. ("Definitions")
**Licensed Practical Nurse**	Likely no. the only reference to scope of practice is with IV authority. 3 CCR 716-1 ("NURSING RULES AND REGULATIONS.")	Likely no. the only reference to scope of practice is with IV authority. 3 CCR 716-1 ("NURSING RULES AND REGULATIONS.")	Likely no. the only reference to scope of practice is with IV authority. 3 CCR 716-1 ("NURSING RULES AND REGULATIONS.")	No. Administration of thrombolytic to declot a catheter specifically listed as outside LPN scope. 3 CCR 716-1:1.9-E ("OUTSIDE THE SCOPE OF IV AUTHORITY")	Unclear. "...participating in the assessment, planning, implementation, and evaluation of the delivery of patient care services..." CRSA 12-255-104. ("Definitions")	Unclear. "...participating in the assessment, planning, implementation, and evaluation of the delivery of patient care services..." CRSA 12-255-104. ("Definitions")	Unclear. "...participating in the assessment, planning, implementation, and evaluation of the delivery of patient care services..." CRSA 12-255-104. ("Definitions")	Likely yes. "...participating in the assessment, planning, implementation, and evaluation of the delivery of patient care services..."CRSA 12-255-104. ("Definitions")
**Physician Assistant**	Maybe, with collaborative practice agreement 3 CCR 713-1:1.15 ("RULES AND REGULATIONS REGARDING THE LICENSURE OF AND PRACTICE BY PHYSICIAN ASSISTANTS")	Maybe, with collaborative practice agreement 3 CCR 713-1:1.15 ("RULES AND REGULATIONS REGARDING THE LICENSURE OF AND PRACTICE BY PHYSICIAN ASSISTANTS")	Maybe, with collaborative practice agreement 3 CCR 713-1:1.15 ("RULES AND REGULATIONS REGARDING THE LICENSURE OF AND PRACTICE BY PHYSICIAN ASSISTANTS")	Maybe. "With a collaborative agreement in place, a physician assistant licensed by the board may perform acts [...] that constitute the practice of medicine and acts that physicians are authorized by law to perform to the extent and in the manner authorized by rules promulgated by the board. C.R.S.A. § 12-240-107 ("Practice of medicine defined--exemptions from licensing requirements--unauthorized practice by physician assistants and anesthesiologist assistants--penalties--definitions--rules")	Maybe. "With a collaborative agreement in place, a physician assistant licensed by the board may perform acts [...] that constitute the practice of medicine and acts that physicians are authorized by law to perform to the extent and in the manner authorized by rules promulgated by the board. C.R.S.A. § 12-240-107 ("Practice of medicine defined--exemptions from licensing requirements--unauthorized practice by physician assistants and anesthesiologist assistants--penalties--definitions--rules")	Maybe. "With a collaborative agreement in place, a physician assistant licensed by the board may perform acts [...] that constitute the practice of medicine and acts that physicians are authorized by law to perform to the extent and in the manner authorized by rules promulgated by the board. C.R.S.A. § 12-240-107 ("Practice of medicine defined--exemptions from licensing requirements--unauthorized practice by physician assistants and anesthesiologist assistants--penalties--definitions--rules")	Maybe. "With a collaborative agreement in place, a physician assistant licensed by the board may perform acts [...] that constitute the practice of medicine and acts that physicians are authorized by law to perform to the extent and in the manner authorized by rules promulgated by the board. C.R.S.A. § 12-240-107 ("Practice of medicine defined--exemptions from licensing requirements--unauthorized practice by physician assistants and anesthesiologist assistants--penalties--definitions--rules")	Maybe. "With a collaborative agreement in place, a physician assistant licensed by the board may perform acts [...] that constitute the practice of medicine and acts that physicians are authorized by law to perform to the extent and in the manner authorized by rules promulgated by the board. C.R.S.A. § 12-240-107 ("Practice of medicine defined--exemptions from licensing requirements--unauthorized practice by physician assistants and anesthesiologist assistants--penalties--definitions--rules")
**Pharmacist**	Maybe. "Prescribing and consultative services pursuant to statewide protocols as defined under Rule 17.00.50 and Appendices, including but not limited to [...]" with no mention of cardiovascular health related drugs. 3 CCR 719-1:17.00.00. ("COLLABORATIVE PHARMACY PRACTICE.")	Maybe. "Prescribing and consultative services pursuant to statewide protocols as defined under Rule 17.00.50 and Appendices, including but not limited to [...]" with no mention of cardiovascular health related drugs. 3 CCR 719-1:17.00.00. ("COLLABORATIVE PHARMACY PRACTICE.")	Maybe. "Prescribing and consultative services pursuant to statewide protocols as defined under Rule 17.00.50 and Appendices, including but not limited to [...]" with no mention of cardiovascular health related drugs. 3 CCR 719-1:17.00.00. ("COLLABORATIVE PHARMACY PRACTICE.")	Maybe, if in collaborative practice agreement. 3 CCR 719-1:17.00.00. ("COLLABORATIVE PHARMACY PRACTICE.")	Maybe, if in collaborative practice agreement. 3 CCR 719-1:17.00.00. ("COLLABORATIVE PHARMACY PRACTICE.")	Maybe, if in collaborative practice agreement. 3 CCR 719-1:17.00.00. ("COLLABORATIVE PHARMACY PRACTICE.")	Maybe, if in collaborative practice agreement. 3 CCR 719-1:17.00.00. ("COLLABORATIVE PHARMACY PRACTICE.")	Maybe, if in collaborative practice agreement. 3 CCR 719-1:17.00.00. ("COLLABORATIVE PHARMACY PRACTICE.")
**Medical Assistant**	No. Prescribing of drugs may not be delegated. CCR 713-1:1.17. ("DELEGATION AND SUPERVISION OF MEDICAL SERVICES TO UNLICENSED PERSONS")	No. Prescribing of drugs may not be delegated. CCR 713-1:1.17. ("DELEGATION AND SUPERVISION OF MEDICAL SERVICES TO UNLICENSED PERSONS")	No. Prescribing of drugs may not be delegated. CCR 713-1:1.17. ("DELEGATION AND SUPERVISION OF MEDICAL SERVICES TO UNLICENSED PERSONS")	Unclear. CCR 713-1:1.17. ("DELEGATION AND SUPERVISION OF MEDICAL SERVICES TO UNLICENSED PERSONS")	Unclear. CCR 713-1:1.17. ("DELEGATION AND SUPERVISION OF MEDICAL SERVICES TO UNLICENSED PERSONS")	Unclear. CCR 713-1:1.17. ("DELEGATION AND SUPERVISION OF MEDICAL SERVICES TO UNLICENSED PERSONS")	Unclear. CCR 713-1:1.17. ("DELEGATION AND SUPERVISION OF MEDICAL SERVICES TO UNLICENSED PERSONS")	Unclear. CCR 713-1:1.17. ("DELEGATION AND SUPERVISION OF MEDICAL SERVICES TO UNLICENSED PERSONS")

**Emergency Medical Technician**	No. CCR 1015–3:2 ("Rules Pertaining to EMS Practice and Medical Director Oversight")	No. CCR 1015–3:2 ("Rules Pertaining to EMS Practice and Medical Director Oversight")	No. CCR 1015–3:2 ("Rules Pertaining to EMS Practice and Medical Director Oversight")	No. CCR 1015-3:2 ("APPENDIX D. FORMULARY OF MEDICATIONS ALLOWED -- INTERFACILITY TRANSPORT") [67]	Likely no. Only reference to ultraound is in Appendix G on page 51 -- "Out of hospital medical services allowed," ultrasound - assist procedures is yes, ultrasound diagnosis is no. [67] CCR 1015–3:2 ("Rules Pertaining to EMS Practice and Medical Director Oversight")	Likely no. CCR 1015–3:2 ("Rules Pertaining to EMS Practice and Medical Director Oversight") [67]	Maybe, at highest EMT level. 6 CCR 1015-3:2 ("APPENDIX A. APPENDIX A. MEDICAL ACTS ALLOWED") [67]	Likely yes. CCR 1015–3:2 ("Rules Pertaining to EMS Practice and Medical Director Oversight") [67]
**Paramedic**	No. CCR 1015–3:2 ("Rules Pertaining to EMS Practice and Medical Director Oversight")	No. CCR 1015–3:2 ("Rules Pertaining to EMS Practice and Medical Director Oversight")	No. CCR 1015–3:2 ("Rules Pertaining to EMS Practice and Medical Director Oversight")	Unclear. Lytics not listed in this document. "Monitoring and Maintenance of thrombolytics allowed" CCR 1015-3:2 ("APPENDIX D. FORMULARY OF MEDICATIONS ALLOWED -- INTERFACILITY TRANSPORT") [67]	Likely No. "ultrasound diagnosis" not allowed but can assist with procedure. CCR 1015-3:2. ("APPENDIX G. OUT-OF-HOSPITAL MEDICAL SERVICES ALLOWED") [67]	Likely no. CCR 1015–3:2 ("Rules Pertaining to EMS Practice and Medical Director Oversight") [67]	Yes. 6 CCR 1015-3:2 ("APPENDIX A. APPENDIX A. MEDICAL ACTS ALLOWED")	Likely yes. CCR 1015–3:2 ("Rules Pertaining to EMS Practice and Medical Director Oversight") [67]
**Community Heath Worker**	No. "facilitate access to physical, behavioral, or dental health-related services, or services to address social determinants of health, and who improves the quality and cultural responsiveness of health-related service delivery." C.R.S.A. § 25.5-5-334. ("Community health worker services--federal authorization--reporting--rules--definition")	No. "facilitate access to physical, behavioral, or dental health-related services, or services to address social determinants of health, and who improves the quality and cultural responsiveness of health-related service delivery." C.R.S.A. § 25.5-5-334. ("Community health worker services--federal authorization--reporting--rules--definition")	No. "facilitate access to physical, behavioral, or dental health-related services, or services to address social determinants of health, and who improves the quality and cultural responsiveness of health-related service delivery." C.R.S.A. § 25.5-5-334. ("Community health worker services--federal authorization--reporting--rules--definition")	No. "facilitate access to physical, behavioral, or dental health-related services, or services to address social determinants of health, and who improves the quality and cultural responsiveness of health-related service delivery." C.R.S.A. § 25.5-5-334. ("Community health worker services--federal authorization--reporting--rules--definition")	No. "facilitate access to physical, behavioral, or dental health-related services, or services to address social determinants of health, and who improves the quality and cultural responsiveness of health-related service delivery." C.R.S.A. § 25.5-5-334. ("Community health worker services--federal authorization--reporting--rules--definition")	No. "facilitate access to physical, behavioral, or dental health-related services, or services to address social determinants of health, and who improves the quality and cultural responsiveness of health-related service delivery." C.R.S.A. § 25.5-5-334. ("Community health worker services--federal authorization--reporting--rules--definition")	No. "facilitate access to physical, behavioral, or dental health-related services, or services to address social determinants of health, and who improves the quality and cultural responsiveness of health-related service delivery." C.R.S.A. § 25.5-5-334. ("Community health worker services--federal authorization--reporting--rules--definition")	No. "facilitate access to physical, behavioral, or dental health-related services, or services to address social determinants of health, and who improves the quality and cultural responsiveness of health-related service delivery." C.R.S.A. § 25.5-5-334. ("Community health worker services--federal authorization--reporting--rules--definition")

Coding Framework = Yes, Likely Yes, Maybe, Unclear, Likely No, No

Idaho	Prescribing antihypertensives	Prescribing heart failure medicines	Monitoring INR and modifying warfarin Rx	Administering thrombolytics for STEMI	Conducting point of care ultrasound screening for heart disease	Conducting Ankle Brachial Index Test	Conducting ECG	Conducting blood pressure test
Nurse Practitioner	Likely Yes - advanced nursing practice may include prescribing, administering, and dispensing of therapeutic pharmacologic agents. I.C. 54-1402. ("Definitions") and IDAPA 24.34.01.200 "Standards of Practice for Advanced Practice Registered Nursing" stating in part "The advanced practice registered nurse shall assume responsibility and accountability for health promotion and maintenance as well as the assessment, diagnosis and management of patient conditions to include the use of pharmacologic and non-pharmacologic interventions and the prescribing and dispensing of pharmacologic and non-pharmacologic agents."	Likely Yes - advanced nursing practice may include prescribing, administering, and dispensing of therapeutic pharmacologic agents. I.C. 54-1402. ("Definitions") and IDAPA 24.34.01.200 "Standards of Practice for Advanced Practice Registered Nursing" stating in part "The advanced practice registered nurse shall assume responsibility and accountability for health promotion and maintenance as well as the assessment, diagnosis and management of patient conditions to include the use of pharmacologic and non-pharmacologic interventions and the prescribing and dispensing of pharmacologic and non-pharmacologic agents."	Likely Yes - advanced nursing practice may include prescribing, administering, and dispensing of therapeutic pharmacologic agents. I.C. 54-1402. ("Definitions") and IDAPA 24.34.01.200 "Standards of Practice for Advanced Practice Registered Nursing" stating in part "The advanced practice registered nurse shall assume responsibility and accountability for health promotion and maintenance as well as the assessment, diagnosis and management of patient conditions to include the use of pharmacologic and non-pharmacologic interventions and the prescribing and dispensing of pharmacologic and non-pharmacologic agents."	Likely Yes - CNP provides initial and ongoing comprehensive primary care services to patients including, but not limited to, diagnosis and management of acute and chronic disease, and health promotion, disease prevention, health education counseling, and identification and management of the effects of illness on patients and their families. IDAPA 24.34.01.002 subsection 07. Certified Nurse Practitioner. ("002. Definitions") and advanced nursing practice may include prescribing, administering, and dispensing of therapeutic pharmacologic agents. I.C. 54-1402. ("Definitions")	Likely Yes - CNP provides initial and ongoing comprehensive primary care services to patients including, but not limited to, diagnosis and management of acute and chronic disease, and health promotion, disease prevention, health education counseling, and identification and management of the effects of illness on patients and their families. IDAPA 24.34.01.002 subsection 07. Certified Nurse Practitioner. ("002. Definitions")	Likely Yes - CNP provides initial and ongoing comprehensive primary care services to patients including, but not limited to, diagnosis and management of acute and chronic disease, and health promotion, disease prevention, health education counseling, and identification and management of the effects of illness on patients and their families. IDAPA 24.34.01.002 subsection 07. Certified Nurse Practitioner. ("002. Definitions")	Likely Yes - CNP provides initial and ongoing comprehensive primary care services to patients including, but not limited to, diagnosis and management of acute and chronic disease, and health promotion, disease prevention, health education counseling, and identification and management of the effects of illness on patients and their families. IDAPA 24.34.01.002 subsection 07. Certified Nurse Practitioner. ("002. Definitions")	Likely Yes - CNP provides initial and ongoing comprehensive primary care services to patients including, but not limited to, diagnosis and management of acute and chronic disease, and health promotion, disease prevention, health education counseling, and identification and management of the effects of illness on patients and their families. IDAPA 24.34.01.002 subsection 07. Certified Nurse Practitioner. ("002. Definitions")
Registered Nurse	Likely No. I.C. 654-1402 subsection 4 ("Definitions")	Likely No. I.C. 654-1402 subsection 4 ("Definitions")	Likely No. I.C. 654-1402 subsection 4 ("Definitions")	Yes - Licensed registered nurse practices nursing by "implementing the strategy of care, including adminstering medications and treatments as prescribed by those health care providers authorized to prescribe medication." I.C. 654-1402 subsection 4. ("Definitions")	Unclear - Licensed registered nurse practices nursing by "assessing health status of groups and individuals…" I.C. 654-1402 subsection 4 ("Definitions")	Unclear - "Licensed registered nurse practices nursing by assessing health status of groups and individuals…" I.C. 654-1402 subsection 4 ("Definitions")	Unclear - "Licensed registered nurse practices nursing by assessing health status of groups and individuals…" I.C. 654-1402 subsection 4 ("Definitions")	Likely yes - "Licensed registered nurse practices nursing by assessing health status of groups and individuals…" I.C. 654-1402 subsection 4 ("Definitions")
Licensed Practical Nurse	Likely No. I.C. 54-1402. Subsection 3. ("Definitions")	Likely No. I.C. 54-1402. Subsection 3. ("Definitions")	Likely No. I.C. 54-1402. Subsection 3. ("Definitions")	Unclear - LPNs practice nursing by "implementing the appropriate aspects of the strategy of care as defined by the board, including administering medications and treatments as prescribed by those health care providers authorized to prescribe medications" No mention of intravenous administration. I.C. 54-1402 subsection 3. ("Definitions")	Unclear - LPN practices nursing by "contributing to the assessment of the health status of individuals or groups of individuals." Work under supervision of RN or higher cadre. I.C. 54-1402. Subsection 3. ("Definitions")	Unclear - LPN practices nursing by "contributing to the assessment of the health status of individuals or groups of individuals." Work under supervision of RN or higher cadre. I.C. 54-1402. Subsection 3. ("Definitions")	Unclear - LPN practices nursing by "contributing to the assessment of the health status of individuals or groups of individuals." Work under supervision of RN or higher cadre. I.C. 54-1402. Subsection 3. ("Definitions"	Likely yes - LPN practices nursing by "contributing to the assessment of the health status of individuals or groups of individuals." Work under supervision of RN or higher cadre. I.C. 54-1402. Subsection 3. ("Definitions")
Physician Assistant	Maybe - if included in collaborative practice agreement. IDAPA 24.33.02.028 ("Scope of Practice"). No longer need to obtain prescribing authority from the board as of 7/1/2021.	Maybe - if included in collaborative practice agreement. IDAPA 24.33.02.028 ("Scope of Practice"). No longer need to obtain prescribing authority from the board as of 7/1/2021.	Maybe - if included in collaborative practice agreement. IDAPA 24.33.02.028 ("Scope of Practice"). No longer need to obtain prescribing authority from the board as of 7/1/2021.	Maybe - If included in collaborative practice agreement. IDAPA 24.33.02.028 ("Scope of Practice")	Maybe - If included in collaborative practice agreement. IDAPA 24.33.02.028 ("Scope of Practice")	Maybe - If included in collaborative practice agreement. IDAPA 24.33.02.028 ("Scope of Practice")	Maybe - If included in collaborative practice agreement. IDAPA 24.33.02.028 ("Scope of Practice")	Maybe - If included in collaborative practice agreement. IDAPA 24.33.02.028 ("Scope of Practice")
Pharmacist	Likely yes - "if act is consistent with the individual's education, training, and experience; and performance of the act is within the accepted standard of care…" I.C. 54-1705 ("Practice of pharmacy--General approach")	Likely yes - "if act is consistent with the individual's education, training, and experience; and performance of the act is within the accepted standard of care…" I.C. 54-1705 ("Practice of pharmacy--General approach")	Likely yes - "if act is consistent with the individual's education, training, and experience; and performance of the act is within the accepted standard of care…" I.C. 54-1705 ("Practice of pharmacy--General approach")	Likely yes - "if act is consistent with the individual's education, training, and experience; and performance of the act is within the accepted standard of care…" I.C. 54-1705 ("Practice of pharmacy--General approach")	Likely yes - "if act is consistent with the individual's education, training, and experience; and performance of the act is within the accepted standard of care…" I.C. 54-1705 ("Practice of pharmacy--General approach")	Likely yes - "if act is consistent with the individual's education, training, and experience; and performance of the act is within the accepted standard of care…" I.C. 54-1705 ("Practice of pharmacy--General approach")	Likely yes - "if act is consistent with the individual's education, training, and experience; and performance of the act is within the accepted standard of care…" I.C. 54-1705 ("Practice of pharmacy--General approach")	Likely yes - "if act is consistent with the individual's education, training, and experience; and performance of the act is within the accepted standard of care…" I.C. 54-1705 ("Practice of pharmacy--General approach")
Medical Assistant	No. IDAPA 24.34.01.002 (subsection 31) ("002. Definitions")	No. IDAPA 24.34.01.002 (subsection 31) ("002. Definitions")	No. IDAPA 24.34.01.002 (subsection 31) ("002. Definitions")	Unclear - considered Unlicensed Assistive Personnel (UAP) who can be delegated to do nursing tasks. IDAPA 24.34.01.002 (subsection 31) ("002. Definitions")	Unclear - considered Unlicensed Assistive Personnel (UAP) who can be delegated to do nursing tasks. IDAPA 24.34.01.002 (subsection 31) ("002. Definitions")	Unclear - considered Unlicensed Assistive Personnel (UAP) who can be delegated to do nursing tasks. IDAPA 24.34.01.002 (subsection 31) ("002. Definitions")	Unclear - considered Unlicensed Assistive Personnel (UAP) who can be delegated to do nursing tasks. IDAPA 24.34.01.002 (subsection 31) ("002. Definitions")	Likely yes - considered Unlicensed Assistive Personnel (UAP) who can be delegated to do nursing tasks. IDAPA 24.34.01.002 (subsection 31) ("002. Definitions")
Emergency Medical Technician	No. ("Idaho EMSPC Scope of Practice - 2020 - 1"). [68]	No. ("Idaho EMSPC Scope of Practice - 2020 - 1"). [68]	No. ("Idaho EMSPC Scope of Practice - 2020 - 1"). [68]	No - not listed at EMT level. Idaho EMSPC. ("Idaho EMSPC Scope of Practice - 2020 - 1"). [68]	No - not listed at EMT level. Idaho EMSPC. ("Idaho EMSPC Scope of Practice - 2020 - 1"). [68]	Likely no. Idaho EMSPC. ("Idaho EMSPC Scope of Practice - 2020 - 1"). [68]	Yes - 12-lead acquisition and transmission. Idaho EMSPC. ("Idaho EMSPC Scope of Practice - 2020 - 1"). [68]	Yes -listed in scope of practice. Idaho EMSPC. ("Idaho EMSPC Scope of Practice - 2020 - 1"). [68]

**Paramedic**	No. ("Idaho EMSPC Scope of Practice - 2020 - 1"). [68]	No. ("Idaho EMSPC Scope of Practice - 2020 - 1"). [68]	No. ("Idaho EMSPC Scope of Practice - 2020 - 1"). [68]	Yes - listed in formulary. Idaho EMSPC. ("Idaho EMSPC Scope of Practice - 2020 - 1"). [68]	Maybe - with additional training and subject to additional standards. Idaho EMSPC. ("Idaho EMSPC Scope of Practice - 2020 - 1"). [68]	Likely no. Idaho EMSPC. ("Idaho EMSPC Scope of Practice - 2020 - 1"). [68]	Yes - 12-lead acquisition, transmission, and interpretation. 3-lead rhythm interpretation. Idaho EMSPC. ("Idaho EMSPC Scope of Practice - 2020 - 1"). [68]	Yes. Listed in scope of practice. Idaho EMSPC. ("Idaho EMSPC Scope of Practice - 2020 - 1"). [68]
**Community Heath Worker**	No - no legal scope of practice	No - no legal scope of practice	No - no legal scope of practice	No - no legal scope of practice	No - no legal scope of practice	No - no legal scope of practice	No - no legal scope of practice	No - no legal scope of practice

Coding Framework = Yes, Likely Yes, Maybe, Unclear, Likely No, No

Michigan	Prescribing antihypertensives	Prescribing heart failure medicines	Monitoring INR and modifying warfarin Rx	Administering thrombolytics for STEMI	Conducting point of care ultrasound screening for heart disease	Conducting Ankle Brachial Index Test	Conducting ECG	Conducting blood pressure test
**Nurse Practitioner**	Likely Yes - prescribing pharmacological interventions listed in definition of NP. MI Admin Code R 338.10401 ("Definitions", subsection d) and MCLA 333.17211a. ("Advanced practice registered nurses; prescriptions"). Must include physician's DEA number.	Likely Yes - prescribing pharmacological interventions listed in definition of NP. MI Admin Code R 338.10401 ("Definitions", subsection d) and MCLA 333.17211a. ("Advanced practice registered nurses; prescriptions"). Must include physician's DEA number.	Likely Yes - prescribing pharmacological interventions listed in definition of NP. MI Admin Code R 338.10401 ("Definitions", subsection d) and MCLA 333.17211a. ("Advanced practice registered nurses; prescriptions"). Must include physician's DEA number.	Likely Yes - "diagnosing, treating, and managing patients with acute and chronic illnesses and diseases" listed in defintion of NP. MI Admin Code R 338.10401 ("Definitions", subsection d)	Likely Yes - "ordering, performing, supervising, and interpreting laboratory and imaging studies" listed in defintion of NP. MI Admin Code R 338.10401 ("Definitions", subsection d)	Likely Yes - "providing physical examinations and other health assessments and screening activities". MI Admin Code R 338.10401 ("Definitions", subsection d)	Likely Yes - ordering, performing, supervising, and interpreting laboratory and imaging studies. MI Admin Code R 338.10401 ("Definitions", subsection d)	Likely Yes - "providing physical examinations and other health assessments and screening activities". MI Admin Code R 338.10401 ("Definitions", subsection d)
**Registered Nurse**	Likely No - prescribing not listed in scope of practice . MCLA 333.17201. ("Definitions; principles of construction")	Likely No - prescribing not listed in scope of practice . MCLA 333.17201. ("Definitions; principles of construction")	Likely No - prescribing not listed in scope of practice . MCLA 333.17201. ("Definitions; principles of construction")	Likely Yes- can administer controlled substances under the order of a licensed prescriber . MCLA 333.7303a (d). ("Powers, duties, and limitations of licensed prescriber; waiver of license requirement"). RN can supervise intravenous administration in CAHs. 42 CFR 485.635. ("Condition of participation: Provision of services")	Unclear "...care, treatment, counsel, and health teaching of individuals..." MCLA 333.17201. ("Definitions; principles of construction")	Unclear "...care, treatment, counsel, and health teaching of individuals..." MCLA 333.17201. ("Definitions; principles of construction")	Unclear - "...care, treatment, counsel, and health teaching of individuals..." MCLA 333.17201. ("Definitions; principles of construction")	Likely Yes - "...care, treatment, counsel, and health teaching of individuals..." MCLA 333.17201. ("Definitions; principles of construction"
**Licensed Practical Nurse**	Likely No - prescribing not listed in scope of practice. MCLA 333.17201. ("Definitions; principles of construction")	Likely No - prescribing not listed in scope of practice. MCLA 333.17201. ("Definitions; principles of construction")	Likely No - prescribing not listed in scope of practice. MCLA 333.17201. ("Definitions; principles of construction")	Unclear- can administer controlled substances under the order of a licensed prescriber . MCLA 333.7303a (d). ("Powers, duties, and limitations of licensed prescriber; waiver of license requirement").	Unclear - "...care, treatment, counsel, and health teaching of individuals..." under the supervision of a registered professional nurse, physician, or dentist. MCLA 333.17201. ("Definitions; principles of construction")	Unclear- "...care, treatment, counsel, and health teaching of individuals..." under the supervision of a registered professional nurse, physician, or dentist. MCLA 333.17201. ("Definitions; principles of construction")	Unclear - "...care, treatment, counsel, and health teaching of individuals..." under the supervision of a registered professional nurse, physician, or dentist. MCLA 333.17201. ("Definitions; principles of construction")	Likely Yes - "...care, treatment, counsel, and health teaching of individuals..." under the supervision of a registered professional nurse, physician, or dentist. MCLA 333.17201. ("Definitions; principles of construction")
**Physician Assistant**	Maybe - a PA may prescribe drugs under a practice agreement with a physician. MCLA 333.17076 ("Physician's assistant; making calls or going on rounds in accordance with practice agreement; prescribing drugs; ordering, receiving, and dispensing complimentary starter dose drugs")	Maybe - a PA may prescribe drugs under a practice agreement with a physician. MCLA 333.17076 ("Physician's assistant; making calls or going on rounds in accordance with practice agreement; prescribing drugs; ordering, receiving, and dispensing complimentary starter dose drugs")	Maybe - a PA may prescribe drugs under a practice agreement with a physician. MCLA 333.17076 ("Physician's assistant; making calls or going on rounds in accordance with practice agreement; prescribing drugs; ordering, receiving, and dispensing complimentary starter dose drugs")	Maybe - can practice medicine with a participating physician under a practice agreement. MCLA 333.17001 (k). ("Definitions; principles of construction")	Maybe - can practice medicine with a participating physician under a practice agreement. MCLA 333.17001 (k). ("Definitions; principles of construction")	Maybe - can practice medicine with a participating physician under a practice agreement. MCLA 333.17001 (k). ("Definitions; principles of construction")	Maybe - can practice medicine with a participating physician under a practice agreement. MCLA 333.17001 (k). ("Definitions; principles of construction")	Maybe - can practice medicine with a participating physician under a practice agreement. MCLA 333.17001 (k). ("Definitions; principles of construction")
**Pharmacist**	Unclear - Michigan law does not explicitly allow pharmacy collaborative practice agreements, but some third part websites indicate pharmacy collaborative practice agreement exist in Michigan MCLA 333-1707 (8) ("Definitions; terms commencing "pa" to "pra").	Unclear - Michigan law does not explicitly allow pharmacy collaborative practice agreements, but some third part websites indicate pharmacy collaborative practice agreement exist in Michigan MCLA 333-1707 (8) ("Definitions; terms commencing "pa" to "pra").	Unclear - Michigan law does not explicitly allow pharmacy collaborative practice agreements, but some third part websites indicate pharmacy collaborative practice agreement exist in Michigan MCLA 333-1707 (8) ("Definitions; terms commencing "pa" to "pra").	Unclear - Michigan law does not explicitly allow pharmacy collaborative practice agreements, but some third part websites indicate pharmacy collaborative practice agreement exist in Michigan MCLA 333-1707 (8) ("Definitions; terms commencing "pa" to "pra").	Unclear - Michigan law does not explicitly allow pharmacy collaborative practice agreements, but some third part websites indicate pharmacy collaborative practice agreement exist in Michigan MCLA 333-1707 (8) ("Definitions; terms commencing "pa" to "pra").	Unclear - Michigan law does not explicitly allow pharmacy collaborative practice agreements, but some third part websites indicate pharmacy collaborative practice agreement exist in Michigan MCLA 333-1707 (8) ("Definitions; terms commencing "pa" to "pra").	Unclear - Michigan law does not explicitly allow pharmacy collaborative practice agreements, but some third part websites indicate pharmacy collaborative practice agreement exist in Michigan MCLA 333-1707 (8) ("Definitions; terms commencing "pa" to "pra").	Unclear - Michigan law does not explicitly allow pharmacy collaborative practice agreements, but some third part websites indicate pharmacy collaborative practice agreement exist in Michigan MCLA 333-1707 (8) ("Definitions; terms commencing "pa" to "pra").
**Medical Assistant**	No -rely on delegation. MCLA 333.16215 (1). Not listed in delegatable tasks ("Tasks Delegable to Medical Assistants") [69]	No -rely on delegation. MCLA 333.16215 (1). Not listed in delegatable tasks ("Tasks Delegable to Medical Assistants") [69]	No -rely on delegation. MCLA 333.16215 (1). Not listed in delegatable tasks ("Tasks Delegable to Medical Assistants") [69]	Likely no - tasks may be delegated to unlicensed professionals. MCLA 333.16215 (1). However, Michigan Society of Medical Assistants states excludes IV adminsitration of drugs in the tasks which may be delegated. ("Tasks Delegable to Medical Assistants") [69]	Unclear - tasks may be delegated to unlicensed professionals. MCLA 333.16215 (1). ("Delegation of acts, tasks, and functions; power of licensees; promulgation of rules")	Unclear - tasks may be delegated to unlicensed professionals. MCLA 333.16215 (1). ("Delegation of acts, tasks, and functions; power of licensees; promulgation of rules")	Unclear - tasks may be delegated to unlicensed professionals. MCLA 333.16215 (1). ("Delegation of acts, tasks, and functions; power of licensees; promulgation of rules")	Unclear - tasks may be delegated to unlicensed professionals. MCLA 333.16215 (1). ("Delegation of acts, tasks, and functions; power of licensees; promulgation of rules")

**Emergency Medical Technician**	No - 333.20956. ("Michigan EMS Scope of Practice") [70]	No - 333.20956. ("Michigan EMS Scope of Practice") [70]	No - 333.20956. ("Michigan EMS Scope of Practice") [70]	No - 333.20956. ("Michigan EMS Scope of Practice") [70]	No - 333.20956. ("Michigan EMS Scope of Practice") [70]	Likely no - 333.20956. ("Michigan EMS Scope of Practice") [70]	Yes - 333.20956. ("Michigan EMS Scope of Practice") [70]	Yes - 333.20956. ("Michigan EMS Scope of Practice") [70]
**Paramedic**	No - 333.20956. ("Michigan EMS Scope of Practice") [70]	No - 333.20956. ("Michigan EMS Scope of Practice") [70]	No - 333.20956. ("Michigan EMS Scope of Practice") [70]	Yes - 333.20956. ("Michigan EMS Scope of Practice") [70]	No - 333.20956. ("Michigan EMS Scope of Practice") [70]	Likely no - 333.20956. ("Michigan EMS Scope of Practice") [70]	Yes - 333.20956. ("Michigan EMS Scope of Practice") [70]	Yes - 333.20956. ("Michigan EMS Scope of Practice") [70]
**Community Heath Worker**	No - no legal scope of practice	No - no legal scope of practice	No - no legal scope of practice	No - no legal scope of practice	No - no legal scope of practice	No - no legal scope of practice	No - no legal scope of practice	No - no legal scope of practice

Coding Framework = Yes, Likely Yes, Maybe, Unclear, Likely No, No

Veterans Health Administration	Prescribing antihypertensives	Prescribing heart failure medicines	Monitoring INR and modifying warfarin Rx	Conducting point of care ultrasound screening for heart disease	Administering thrombolytics for STEMI	Conducting Ankle Brachial Index Test	Conducting ECG	Conducting blood pressure test
**Nurse Practitioner**	Yes - NPs have full practice authority including prescribing, screening, diagnosing, and treating. 38 C.F.R. 17.415. ("Full practice authority for advanced practice registered nurses")	Yes - NPs have full practice authority including prescribing, screening, diagnosing, and treating. 38 C.F.R. 17.415. ("Full practice authority for advanced practice registered nurses")	Yes - NPs have full practice authority including prescribing, screening, diagnosing, and treating. 38 C.F.R. 17.415. ("Full practice authority for advanced practice registered nurses")	Yes - NPs have full practice authority including prescribing, screening, diagnosing, and treating. 38 C.F.R. 17.415. ("Full practice authority for advanced practice registered nurses")	Yes - NPs have full practice authority including prescribing, screening, diagnosing, and treating. 38 C.F.R. 17.415. ("Full practice authority for advanced practice registered nurses")	Yes - NPs have full practice authority including prescribing, screening, diagnosing, and treating. 38 C.F.R. 17.415. ("Full practice authority for advanced practice registered nurses")	Yes - NPs have full practice authority including prescribing, screening, diagnosing, and treating. 38 C.F.R. 17.415. ("Full practice authority for advanced practice registered nurses")	Yes - NPs have full practice authority including prescribing, screening, diagnosing, and treating. 38 C.F.R. 17.415. ("Full practice authority for advanced practice registered nurses")
**Registered Nurse**	Likely no. Practice description for RN varies and Nurse I - V do not list prescribing. VA Handbook 5005/160 (pages 6 - 14) [71]	Likely no. Practice description for RN varies and Nurse I - V do not list prescribing. VA Handbook 5005/160 (pages 6 - 14) [71]	Maybe. "before implementing use of nursing medication management protocols … review the individual RN's state license and the corresponding state board of nursing statutes and regulations…" VHA Directive 1108.13(1) Background (c)(page 2) - [72]	Unclear. Practice for all Nurse levels lists the nursing process as "assessment, diagnosis, outcome identification, planning implementation and evaluation" VA Handbook 5005/160 (pages 6 - 14) [71]	Yes. Permitted for LVN supervised by RN. [74] Practice for all Nurse levels lists the nursing process as "assessment, diagnosis, outcome identification, planning implementation and evaluation" VA Handbook 5005/160 (pages 6 - 14) [71]	Unclear. Practice for all Nurse levels lists the nursing process as "assessment, diagnosis, outcome identification, planning implementation and evaluation" VA Handbook 5005/160 (pages 6 - 14) [71]	Unclear. Practice for all Nurse levels lists the nursing process as "assessment, diagnosis, outcome identification, planning implementation and evaluation" VA Handbook 5005/160 (pages 6 - 14) [71]	Likely yes. Practice for all Nurse levels lists the nursing process as "assessment, diagnosis, outcome identification, planning implementation and evaluation" VA Handbook 5005/160 (pages 6 - 14) [71]
**Licensed Practical Nurse**	Likely no. Prescribing not listed in duties of LPN/LVN. VA Nursing Service: FACT SHEET. [73]	Likely no. Prescribing not listed in duties of LPN/LVN. VA Nursing Service: FACT SHEET. [73]	Likely no. Prescribing not listed in duties of LPN/LVN. VA Nursing Service: FACT SHEET. [73]	Unclear. "Provide a full range of services … personal care, diagnostic support duties, medication administration, treatments and procedures, under the supervision of an RN." VA Nursing Service: FACT SHEET. [73]	Yes. "Provide a full range of services … personal care, diagnostic support duties, medication administration, treatments and procedures, under the supervision of an RN." VA Nursing Service: FACT SHEET. [73]	Unclear. "Provide a full range of services … personal care, diagnostic support duties, medication administration, treatments and procedures, under the supervision of an RN." VA Nursing Service: FACT SHEET. [73]	Unclear. "Provide a full range of services … personal care, diagnostic support duties, medication administration, treatments and procedures, under the supervision of an RN." VA Nursing Service: FACT SHEET. [73]	Likely yes. "Provide a full range of services … personal care, diagnostic support duties, medication administration, treatments and procedures, under the supervision of an RN." VA Nursing Service: FACT SHEET. [73]
**Physician Assistant**	Maybe. PA must work with a collaborating physician and Initial, renewal, and modification of Scopes of Practice must be approved by the Executive Committee of the Medical Staff and the medical facility Director, using the Medical Staff process. PAs must not function in a patient care role without an approved Scope of Practice. ("Utilization of Physician Assistants (PA)") [74]	Maybe. PA must work with a collaborating physician and Initial, renewal, and modification of Scopes of Practice must be approved by the Executive Committee of the Medical Staff and the medical facility Director, using the Medical Staff process. PAs must not function in a patient care role without an approved Scope of Practice. ("Utilization of Physician Assistants (PA)") [74]	Maybe. PA must work with a collaborating physician and Initial, renewal, and modification of Scopes of Practice must be approved by the Executive Committee of the Medical Staff and the medical facility Director, using the Medical Staff process. PAs must not function in a patient care role without an approved Scope of Practice. ("Utilization of Physician Assistants (PA)") [74]	Maybe. PA must work with a collaborating physician and Initial, renewal, and modification of Scopes of Practice must be approved by the Executive Committee of the Medical Staff and the medical facility Director, using the Medical Staff process. PAs must not function in a patient care role without an approved Scope of Practice. ("Utilization of Physician Assistants (PA)") [74]	Maybe. PA must work with a collaborating physician and Initial, renewal, and modification of Scopes of Practice must be approved by the Executive Committee of the Medical Staff and the medical facility Director, using the Medical Staff process. PAs must not function in a patient care role without an approved Scope of Practice. ("Utilization of Physician Assistants (PA)") [74]	Maybe. PA must work with a collaborating physician and Initial, renewal, and modification of Scopes of Practice must be approved by the Executive Committee of the Medical Staff and the medical facility Director, using the Medical Staff process. PAs must not function in a patient care role without an approved Scope of Practice. ("Utilization of Physician Assistants (PA)") [74]	Maybe. PA must work with a collaborating physician and Initial, renewal, and modification of Scopes of Practice must be approved by the Executive Committee of the Medical Staff and the medical facility Director, using the Medical Staff process. PAs must not function in a patient care role without an approved Scope of Practice. ("Utilization of Physician Assistants (PA)") [74]	Maybe. PA must work with a collaborating physician and Initial, renewal, and modification of Scopes of Practice must be approved by the Executive Committee of the Medical Staff and the medical facility Director, using the Medical Staff process. PAs must not function in a patient care role without an approved Scope of Practice. ("Utilization of Physician Assistants (PA)") [74]
**Pharmacist**	Maybe. Collaborative practice agreement can include, "the authority to presribe … order related lab tests and diagnostic studies, perform physical measurements and objective assessments, and other necessary actions to facilitate patient care." ("VA Handbook 1108.11 (pages 1-2)") [75]	Maybe. Collaborative practice agreement can include, "the authority to presribe … order related lab tests and diagnostic studies, perform physical measurements and objective assessments, and other necessary actions to facilitate patient care." ("VA Handbook 1108.11 (pages 1-2)") [75]	Maybe. Collaborative practice agreement can include, "the authority to presribe … order related lab tests and diagnostic studies, perform physical measurements and objective assessments, and other necessary actions to facilitate patient care." ("VA Handbook 1108.11 (pages 1-2)") [75]	Maybe. Collaborative practice agreement can include, "the authority to presribe … order related lab tests and diagnostic studies, perform physical measurements and objective assessments, and other necessary actions to facilitate patient care." ("VA Handbook 1108.11 (pages 1-2)") [75]	Maybe. Collaborative practice agreement can include, "the authority to presribe … order related lab tests and diagnostic studies, perform physical measurements and objective assessments, and other necessary actions to facilitate patient care." ("VA Handbook 1108.11 (pages 1-2)") [75]	Maybe. Collaborative practice agreement can include, "the authority to presribe … order related lab tests and diagnostic studies, perform physical measurements and objective assessments, and other necessary actions to facilitate patient care." ("VA Handbook 1108.11 (pages 1-2)") [75]	Maybe. Collaborative practice agreement can include, "the authority to presribe … order related lab tests and diagnostic studies, perform physical measurements and objective assessments, and other necessary actions to facilitate patient care." ("VA Handbook 1108.11 (pages 1-2)") [75]	Maybe. Collaborative practice agreement can include, "the authority to presribe … order related lab tests and diagnostic studies, perform physical measurements and objective assessments, and other necessary actions to facilitate patient care." ("VA Handbook 1108.11 (pages 1-2)") [75]
**Medical Assistant**	Does not have VA National Standard of Practice. ("VA National Standards of Practice") [76]	Does not have VA National Standard of Practice. ("VA National Standards of Practice") [76]	Does not have VA National Standard of Practice. ("VA National Standards of Practice") [76]	Does not have VA National Standard of Practice. ("VA National Standards of Practice") [76]	Does not have VA National Standard of Practice. ("VA National Standards of Practice") [76]	Does not have VA National Standard of Practice. ("VA National Standards of Practice") [76]	Does not have VA National Standard of Practice. ("VA National Standards of Practice") [76]	Does not have VA National Standard of Practice. ("VA National Standards of Practice") [76]
**Emergency Medical Technician**	Unclear. Document suggests that there is a national standard of practice for EMS in the VA, but cannot find online. ("VA National Standards of Practice")[76]	Unclear. Document suggests that there is a national standard of practice for EMS in the VA, but cannot find online. ("VA National Standards of Practice")[76]	Unclear. Document suggests that there is a national standard of practice for EMS in the VA, but cannot find online. ("VA National Standards of Practice")[76]	Unclear. Document suggests that there is a national standard of practice for EMS in the VA, but cannot find online. ("VA National Standards of Practice")[76]	Unclear. Document suggests that there is a national standard of practice for EMS in the VA, but cannot find online. ("VA National Standards of Practice")[76]	Unclear. Document suggests that there is a national standard of practice for EMS in the VA, but cannot find online. ("VA National Standards of Practice")[76]	Unclear. Document suggests that there is a national standard of practice for EMS in the VA, but cannot find online. ("VA National Standards of Practice")[76]	Unclear. Document suggests that there is a national standard of practice for EMS in the VA, but cannot find online. ("VA National Standards of Practice")[76]

**Paramedic**	Unclear. Document suggests that there is a national standard of practice for EMS in the VA, but cannot find online. [76] USAJOBS.gov listing suggests VA paramedics are called "Medical Transportation Techs"	Unclear. Document suggests that there is a national standard of practice for EMS in the VA, but cannot find online. [76] USAJOBS.gov listing suggests VA paramedics are called "Medical Transportation Techs"	Unclear. Document suggests that there is a national standard of practice for EMS in the VA, but cannot find online. [76] USAJOBS.gov listing suggests VA paramedics are called "Medical Transportation Techs"	Unclear. Document suggests that there is a national standard of practice for EMS in the VA, but cannot find online. [76] USAJOBS.gov listing suggests VA paramedics are called "Medical Transportation Techs"	Unclear. Document suggests that there is a national standard of practice for EMS in the VA, but cannot find online. [76] USAJOBS.gov listing suggests VA paramedics are called "Medical Transportation Techs"	Unclear. Document suggests that there is a national standard of practice for EMS in the VA, but cannot find online. [76] USAJOBS.gov listing suggests VA paramedics are called "Medical Transportation Techs"	Unclear. Document suggests that there is a national standard of practice for EMS in the VA, but cannot find online. [76] USAJOBS.gov listing suggests VA paramedics are called "Medical Transportation Techs"	Unclear. Document suggests that there is a national standard of practice for EMS in the VA, but cannot find online. [76] USAJOBS.gov listing suggests VA paramedics are called "Medical Transportation Techs"
**Community Heath Worker**	Does not have VA National Standard of Practice [76]	Does not have VA National Standard of Practice [76]	Does not have VA National Standard of Practice [76]	Does not have VA National Standard of Practice [76]	Does not have VA National Standard of Practice [76]	Does not have VA National Standard of Practice [76]	Does not have VA National Standard of Practice [76]	Does not have VA National Standard of Practice [76]

Coding Framework = Yes, Likely Yes, Maybe, Unclear, Likely No, No

Montana	Prescribing antihypertensives	Prescribing heart failure medicines	Monitoring INR and modifying warfarin Rx in anticoagulation clinics	Administering thrombolytics for STEMI	Conducting point of care ultrasound screening for heart disease	Conducting Ankle Brachial Index Test	Conducting an ECG/EKG	Conducting blood pressure test
**Nurse Practitioner**	Maybe - APRN must apply for prescriptive authority and complete graduate training in pharmacology. Montana Administrative Rule 24.159.1463 ("Application for prescriptive authority"); and Montana Administrative Regulation 24.159.1406 (stating "(ii) prescribing legend and controlled substances when prescriptive authority is successfully applied for and obtained")	Maybe - APRN must apply for prescriptive authority and complete graduate training in pharmacology. Montana Administrative Rule 24.159.1463 ("Application for prescriptive authority"); and Montana Administrative Regulation 24.159.1406 (stating "(ii) prescribing legend and controlled substances when prescriptive authority is successfully applied for and obtained")	Maybe - APRN must apply for prescriptive authority and complete graduate training in pharmacology. Montana Administrative Rule 24.159.1463 ("Application for prescriptive authority"); and Montana Administrative Regulation 24.159.1406 (stating "(ii) prescribing legend and controlled substances when prescriptive authority is successfully applied for and obtained")	Likely Yes - APRNs have full practice authority in Montana. Montana Administrative Rule 24.159.1406. ("APRN practice").	Likely Yes - APRN practice "stating scope includes "(i) physical examinations, health assessments, and/or other screening activities;" Mont. Admin. R. 24.159.1406 ("APRN PRACTICE")	Likely Yes - APRN practice "stating scope includes "(i) physical examinations, health assessments, and/or other screening activities;" Mont. Admin. R. 24.159.1406 ("APRN PRACTICE")	Likely Yes - APRN practice "stating scope includes "(i) physical examinations, health assessments, and/or other screening activities;" Mont. Admin. R. 24.159.1406 ("APRN PRACTICE")	Likely Yes - APRN practice "stating scope includes "(i) physical examinations, health assessments, and/or other screening activities;" Mont. Admin. R. 24.159.1406 ("APRN PRACTICE")
**Registered Nurse**	Likely No - scope of practice does not include prescribing (Montana Administrative Rule 24.159.1204).	Likely No - scope of practice does not include prescribing (Montana Administrative Rule 24.159.1204).	Likely No - scope of practice does not include prescribing (Montana Administrative Rule 24.159.1204).	Unclear - No mention of medication administration. Montana Administrative Rule 24.159.1204. ("Standards related to the registered nurse's responsibility to apply the nursing process")	Unclear - "collecting objective and subjective data from observations, examinations…" Montana Administrative Rule 24.159.1204. ("Standards related to the registered nurse's responsibility to apply the nursing process")	Unclear - "collecting objective and subjective data from observations, examinations…" Montana Administrative Rule 24.159.1204. ("Standards related to the registered nurse's responsibility to apply the nursing process")	Unclear - "collecting objective and subjective data from observations, examinations…" Montana Administrative Rule 24.159.1204. ("Standards related to the registered nurse's responsibility to apply the nursing process")	Likely Yes - "collecting objective and subjective data from observations, examinations…" Montana Administrative Rule 24.159.1204. ("Standards related to the registered nurse's responsibility to apply the nursing process")
**Licensed Practical Nurse**	Likely No - prescribing not included in practical nurse scope of practice (STANDARDS RELATED TO THE PRACTICAL NURSE'S APPLICATION OF THE NURSING PROCESS Mont. Admin. R. 24.159.1004)	Likely No - prescribing not included in practical nurse scope of practice (STANDARDS RELATED TO THE PRACTICAL NURSE'S APPLICATION OF THE NURSING PROCESS Mont. Admin. R. 24.159.1004)	Likely No - prescribing not included in practical nurse scope of practice (STANDARDS RELATED TO THE PRACTICAL NURSE'S APPLICATION OF THE NURSING PROCESS Mont. Admin. R. 24.159.1004)	No - thrombolytics listed as prohibited intravenous therapy for LPNs. Montana Rule 24.159.1011 . ("Prohibited intravenous therapies")	Unclear- "collecting, reporting, and recording objective and subjective data. Data collection includes: observation about the condition or change in condition of the client; and signs and symptoms of deviation of normal health status." Montana Administrative Rule 24.159.1004. ("Standards related to the practical nurse's applicaiton of the nursing process")	Unclear- "collecting, reporting, and recording objective and subjective data. Data collection includes: observation about the condition or change in condition of the client; and signs and symptoms of deviation of normal health status." Montana Administrative Rule 24.159.1004. ("Standards related to the practical nurse's applicaiton of the nursing process")	Unclear- "collecting, reporting, and recording objective and subjective data. Data collection includes: observation about the condition or change in condition of the client; and signs and symptoms of deviation of normal health status." Montana Administrative Rule 24.159.1004. ("Standards related to the practical nurse's applicaiton of the nursing process")	Likely Yes - "collecting, reporting, and recording objective and subjective data. Data collection includes: observation about the condition or change in condition of the client; and signs and symptoms of deviation of normal health status." Montana Administrative Rule 24.159.1004. ("Standards related to the practical nurse's applicaiton of the nursing process")
**Physician Assistant**	Likely Yes - PA may prescribe, dispense, and administer drugs. Montana Code Annotated 37-20-404. ("Prescribing and dispensing authority" stating "(1) A physician assistant may prescribe, dispense, and administer drugs.")	Likely Yes - PA may prescribe, dispense, and administer drugs. Montana Code Annotated 37-20-404. ("Prescribing and dispensing authority" stating "(1) A physician assistant may prescribe, dispense, and administer drugs.")	Likely Yes - PA may prescribe, dispense, and administer drugs. Montana Code Annotated 37-20-404. ("Prescribing and dispensing authority" stating "(1) A physician assistant may prescribe, dispense, and administer drugs.")	Likely Yes - PA may prescribe, dispense, and administer drugs. Montana Code Annotated 37-20-404. ("Prescribing and dispensing authority" stating "(1) A physician assistant may prescribe, dispense, and administer drugs.")	Likely Yes - a physician assistant may "diagnose, examine, and treat human conditions, ailments, disease, injuries, or infirmities, either physical or mental, by any means, method, device, or instrumentality." Montana Code Annotated 37-20-403. ("Physician assistant scope of practice"). Physician assistants with less than 8,000 postgrad clinical hours must practice under a collaborative practice agreement or PA with 8,000+ clinical hours. Montana Code Annotated 37-20-203. ("Licenseing of physician assistants - collaborative agreements")	Likely Yes - a physician assistant may "diagnose, examine, and treat human conditions, ailments, disease, injuries, or infirmities, either physical or mental, by any means, method, device, or instrumentality." Montana Code Annotated 37-20-403. ("Physician assistant scope of practice"). Physician assistants with less than 8,000 postgrad clinical hours must practice under a collaborative practice agreement or PA with 8,000+ clinical hours. Montana Code Annotated 37-20-203. ("Licenseing of physician assistants - collaborative agreements")	Likely Yes - a physician assistant may "diagnose, examine, and treat human conditions, ailments, disease, injuries, or infirmities, either physical or mental, by any means, method, device, or instrumentality." Montana Code Annotated 37-20-403. ("Physician assistant scope of practice"). Physician assistants with less than 8,000 postgrad clinical hours must practice under a collaborative practice agreement or PA with 8,000+ clinical hours. Montana Code Annotated 37-20-203. ("Licenseing of physician assistants - collaborative agreements")	Likely yes - a physician assistant may "diagnose, examine, and treat human conditions, ailments, disease, injuries, or infirmities, either physical or mental, by any means, method, device, or instrumentality." Montana Code Annotated 37-20-403. ("Physician assistant scope of practice"). Physician assistants with less than 8,000 postgrad clinical hours must practice under a collaborative practice agreement or PA with 8,000+ clinical hours. Montana Code Annotated 37-20-203. ("Licenseing of physician assistants - collaborative agreements")

**Pharmacist**	Maybe - pharmacist prescribing authority limited to "not requiring a new diagnosis" and "minor and generally self-limiting." Montana Code Annotated. 37-7-106. (Pharmacist prescribing authority -- exception"). Otherwise maybe if included in terms of collaborative practice agreement. Montana Administrative Rule 24.174.524 "Collaborative practice agreement requirements")	Maybe - pharmacist prescribing authority limited to "not requiring a new diagnosis" and "minor and generally self-limiting." Montana Code Annotated. 37-7-106. (Pharmacist prescribing authority -- exception"). Otherwise maybe if included in terms of collaborative practice agreement. Montana Administrative Rule 24.174.524 "Collaborative practice agreement requirements")	Maybe - pharmacist prescribing authority limited to "not requiring a new diagnosis" and "minor and generally self-limiting." Montana Code Annotated. 37-7-106. (Pharmacist prescribing authority -- exception"). Otherwise maybe if included in terms of collaborative practice agreement. Montana Administrative Rule 24.174.524 "Collaborative practice agreement requirements")	Maybe - possible if included in collaborative practice agreement, Montana Administrative Rule 24.174.524. ("Collaborative practice agreement requirements")	Maybe - possible if included in collaborative practice agreement. Montana Administrative Rule 24.174.524. ("Collaborative practice agreement requirements")	Maybe - possible if in collaborative practice agreement. Montana Administrative Rule 24.174.524. ("Collaborative practice agreement requirements")	Maybe - possible if included in collaborative practice agreement, Montana Administrative Rule 24.174.524. ("Collaborative practice agreement requirements")	Maybe - possible if included in collaborative practice agreement, Montana Administrative Rule 24.174.524. ("Collaborative practice agreement requirements")
**Medical Assistant**	Likely no - health care providers have broad authority to delegate tasks to medical assistants that are within the "education, training, knowledge, and skill to perform", but prescribing is likely not within the training or education of medical assistants (24.156.401. MEDICAL ASSISTANT -- DELEGATION AND SUPERVISION, Mont. Admin. R. 24.156.401)	Likely no - health care providers have broad authority to delegate tasks to medical assistants that are within the "education, training, knowledge, and skill to perform", but prescribing is likely not within the training or education of medical assistants (24.156.401. MEDICAL ASSISTANT -- DELEGATION AND SUPERVISION, Mont. Admin. R. 24.156.401)	Likely no - health care providers have broad authority to delegate tasks to medical assistants that are within the "education, training, knowledge, and skill to perform", but prescribing is likely not within the training or education of medical assistants (24.156.401. MEDICAL ASSISTANT -- DELEGATION AND SUPERVISION, Mont. Admin. R. 24.156.401)	Unclear - If delegated to MA by authorized health care provider. Montana Administrative Rule 24.156.401. ("Medical Assistant -- delegation and supervision"), see also Montana Code Annotated 2023 37-3-104. ("Medical Assistants -- Guidelines")	Unclear - If delegated to MA by authorized health care provider. Montana Administrative Rule 24.156.401. ("Medical Assistant -- delegation and supervision"), see also Montana Code Annotated 2023 37-3-104. ("Medical Assistants -- Guidelines")	Unclear - If delegated to MA by authorized health care provider. Montana Administrative Rule 24.156.401. ("Medical Assistant -- delegation and supervision"), see also Montana Code Annotated 2023 37-3-104. ("Medical Assistants -- Guidelines")	Unclear - If delegated to MA by authorized health care provider. Montana Administrative Rule 24.156.401. ("Medical Assistant -- delegation and supervision"), see also Montana Code Annotated 2023 37-3-104. ("Medical Assistants -- Guidelines")	Unclear - If delegated to MA by authorized health care provider. Montana Administrative Rule 24.156.401. ("Medical Assistant -- delegation and supervision"), see also Montana Code Annotated 2023 37-3-104. ("Medical Assistants -- Guidelines")
**Emergency Medical Technician**	No - EMT scope of practice does not include general prescribing authority (ECP SCOPE OF PRACTICE, Mont. Admin. R. 24.156.2771)	No - EMT scope of practice does not include general prescribing authority (ECP SCOPE OF PRACTICE, Mont. Admin. R. 24.156.2771)	No - EMT scope of practice does not include general prescribing authority (ECP SCOPE OF PRACTICE, Mont. Admin. R. 24.156.2771)	Likely no - EMT Scope of Practice does not mention administering thrombolytics and fibrinolytic not listed for EMT level. ("Montana Prehospital Treatment Protocols"). [77]	Likely no - EMT scope of practice does not reference ultrasound or imaging Montana 24.156.2771. ("ECP Scope of Practice")[77].	Likely no - practice guidelines do not reference ankle brachial index test (Administrative Rules of Montana 24.156.2771, ECP Scope of Practice";"Montana Prehospital Treatment Protocols"). [77]	No - interpretting EKG limited to paramedic (Administrative Rules of Montana 24.156.2771, ECP Scope of Practice";"Montana Prehospital Treatment Protocols").	Yes - Montana ECP Scope of Practice includes obtaining and recording vital signs. ("Montana Prehospital Treatment Protocols")[77] Administrative Rules of Montana 24.156.2771. ("ECP Scope of Practice").
**Paramedic**	No - paramedic scope of practice does not include general prescribing authority (ECP SCOPE OF PRACTICE, Mont. Admin. R. 24.156.2771)	No - paramedic scope of practice does not include general prescribing authority (ECP SCOPE OF PRACTICE, Mont. Admin. R. 24.156.2771)	No - paramedic scope of practice does not include general prescribing authority (ECP SCOPE OF PRACTICE, Mont. Admin. R. 24.156.2771)	Maybe - practice guidelines include reference for paramedics to consider fibrinolytic if EKG shows STEMI and includes a reference tp "(per local protocol)" ("Montana Prehospital Treatment Protocols") [77]	Likely no - paramedic scope of practice does not reference ultrasound or imaging Montana 24.156.2771. ("ECP Scope of Practice")[77].	Likely no - practice guidelines do not reference ankle brachial index test (Administrative Rules of Montana 24.156.2771, ECP Scope of Practice";"Montana Prehospital Treatment Protocols"). [77]	Likely yes - paramedic practice guidelines reference interpret ECG ("Montana Prehospital Treatment Protocols"). [77]	Yes - scope includes obtaining and recording vital signs. ("Montana Prehospital Treatment Protocols"). [77]
**Community Heath Worker**	No - CHWs do not have a licensed scope of practice in MT and Montana Code Annotated 2023 37-3-101 states "practice of medicine within the state of Montana is a privilege granted by the legislative authority…". Montana Code Annotated 2023 37-3-101 ("Purpose")	No - CHWs do not have a licensed scope of practice in MT and Montana Code Annotated 2023 37-3-101 states "practice of medicine within the state of Montana is a privilege granted by the legislative authority…". Montana Code Annotated 2023 37-3-101 ("Purpose")	No - CHWs do not have a licensed scope of practice in MT and Montana Code Annotated 2023 37-3-101 states "practice of medicine within the state of Montana is a privilege granted by the legislative authority…". Montana Code Annotated 2023 37-3-101 ("Purpose")	No - CHWs do not have a licensed scope of practice in MT and Montana Code Annotated 2023 37-3-101 states "practice of medicine within the state of Montana is a privilege granted by the legislative authority…". Montana Code Annotated 2023 37-3-101 ("Purpose")	No - CHWs do not have a licensed scope of practice in MT and Montana Code Annotated 2023 37-3-101 states "practice of medicine within the state of Montana is a privilege granted by the legislative authority…". Montana Code Annotated 2023 37-3-101 ("Purpose")	No - CHWs do not have a licensed scope of practice in MT and Montana Code Annotated 2023 37-3-101 states "practice of medicine within the state of Montana is a privilege granted by the legislative authority…". Montana Code Annotated 2023 37-3-101 ("Purpose")	No - CHWs do not have a licensed scope of practice in MT and Montana Code Annotated 2023 37-3-101 states "practice of medicine within the state of Montana is a privilege granted by the legislative authority…". Montana Code Annotated 2023 37-3-101 ("Purpose")	No - CHWs do not have a licensed scope of practice in MT and Montana Code Annotated 2023 37-3-101 states "practice of medicine within the state of Montana is a privilege granted by the legislative authority…". Montana Code Annotated 2023 37-3-101 ("Purpose")

Coding Framework = Yes, Likely Yes, Maybe, Unclear, Likely No, No

North Carolina	Prescribing antihypertensives	Prescribing heart failure medicines	Monitoring INR and modifying warfarin Rx	Administering thrombolytics for STEMI	Conducting point of care ultrasound screening for heart disease	Conducting Ankle Brachial Index Test	Conducting ECG	Conducting blood pressure test
**Nurse Practitioner**	Maybe - scope includes "prescribing, administering, and dispensing therapeutic measures, tests, procedures, and drugs" 21 N.C. Admin. Code 36.0802. Must be included in collaborative practice agreement. 21 NCAC 32M.0109. ("Prescribing authority")	Maybe - scope includes "prescribing, administering, and dispensing therapeutic measures, tests, procedures, and drugs" 21 N.C. Admin. Code 36.0802. Must be included in collaborative practice agreement. 21 NCAC 32M.0109. ("Prescribing authority")	Maybe - scope includes "prescribing, administering, and dispensing therapeutic measures, tests, procedures, and drugs" 21 N.C. Admin. Code 36.0802. Must be included in collaborative practice agreement. 21 NCAC 32M.0109. ("Prescribing authority")	Likely Yes - scope includes "prescribing, administering, and dispensing therapeutic measures, tests, procedures, and drugs;" 21 N.C. Admin. Code 36.0802	Likely Yes - scope includes "prescribing, administering, and dispensing therapeutic measures, tests, procedures, and drugs;" 21 N.C. Admin. Code 36.0803	Likely Yes - scope includes "prescribing, administering, and dispensing therapeutic measures, tests, procedures, and drugs;" 21 N.C. Admin. Code 36.0804	Likely Yes - scope includes "prescribing, administering, and dispensing therapeutic measures, tests, procedures, and drugs;" 21 N.C. Admin. Code 36.0805	Likely Yes - scope includes "prescribing, administering, and dispensing therapeutic measures, tests, procedures, and drugs;" 21 N.C. Admin. Code 36.0806
**Registered Nurse**	Likely no - Practice of nursing by a registered nurse does not include prescribing, but does allow a registered nurse to prescribe treatments under the supervision of a physician N.C.G.S.A. § 90-171.20 ("Definitions")	Likely no - Practice of nursing by a registered nurse does not include prescribing, but does allow a registered nurse to prescribe treatments under the supervision of a physician N.C.G.S.A. § 90-171.20 ("Definitions")	Likely no - Practice of nursing by a registered nurse does not include prescribing, but does allow a registered nurse to prescribe treatments under the supervision of a physician N.C.G.S.A. § 90-171.20 ("Definitions")	Likely Yes - Implementing the treatment and pharmaceutical regimen prescribed by any person authorized by State law to prescribe the regimen. N.C.G.S.A. § 90-171.20 (subsection 7) ("Definitions") Practice of nursing by a registered nurse described here N.C.G.S.A. § 90-171.20 ("Definitions")	Unclear - RN scope includes "Assessing the patient's physical and mental health, including the patient's reaction to illnesses and treatment regimens." N.C. Gen. Stat. Ann. § 90-171.20" and see 21 NCAC 36.0224. (".0224 Components of Nursing Practice for the Registered Nurse")	Unclear - RN scope includes "Assessing the patient's physical and mental health, including the patient's reaction to illnesses and treatment regimens." N.C. Gen. Stat. Ann. § 90-171.20" and see 21 NCAC 36.0224. (".0224 Components of Nursing Practice for the Registered Nurse")	Unclear - RN scope includes "Assessing the patient's physical and mental health, including the patient's reaction to illnesses and treatment regimens." N.C. Gen. Stat. Ann. § 90-171.20" and see 21 NCAC 36.0224. (".0224 Components of Nursing Practice for the Registered Nurse")	Likely Yes - RN scope includes "Assessing the patient's physical and mental health, including the patient's reaction to illnesses and treatment regimens." N.C. Gen. Stat. Ann. § 90-171.20" and see 21 NCAC 36.0224. (".0224 Components of Nursing Practice for the Registered Nurse")
**Licensed Practical Nurse**	Likely No - Practice of nursing by a practical nurse does not include prescribing authority N.C.G.S.A. § 90-171.20 ("Definitions")	Likely No - Practice of nursing by a practical nurse does not include prescribing authority N.C.G.S.A. § 90-171.20 ("Definitions")	Likely No - Practice of nursing by a practical nurse does not include prescribing authority N.C.G.S.A. § 90-171.20 ("Definitions")	Likely no - LPN scope does not include independent authority to administer pharmaceuticals N.C.G.S.A. § 90-171.20 (subsection 8) ("Definitions")	Likely no - 21 NCAC 36.0225, (".0225 Components of Nursing Practice for the Licensed Practical Nurse") Practice of nursing by a practical nurse described here N.C.G.S.A. § 90-171.20 ("Definitions")	Likely no - 21 NCAC 36.0225, (".0225 Components of Nursing Practice for the Licensed Practical Nurse") Practice of nursing by a practical nurse described here N.C.G.S.A. § 90-171.20 ("Definitions")	Likely no - 21 NCAC 36.0225, (".0225 Components of Nursing Practice for the Licensed Practical Nurse") Practice of nursing by a practical nurse described here N.C.G.S.A. § 90-171.20 ("Definitions")	Unclear - LPN scope does not reference independent assessment of patient's health (".0225 Components of Nursing Practice for the Licensed Practical Nurse") Practice of nursing by a practical nurse described here N.C.G.S.A. § 90-171.20 ("Definitions")
**Physician Assistant**	Maybe - a PA may perform medical acts, tasks, or functions under the supervision of a physician but scope of practice must be defined in a supervisory agreement, which must include "written prescribing instructions" 21 N.C. Admin. Code 32S.0213. NCAC 32S.0213. (".0213 Physician Supervision of Physician Assistants"). See details on prescriptive authority. 21 NCAC 32S.0212. ("Prescriptive authority")	Maybe - a PA may perform medical acts, tasks, or functions under the supervision of a physician but scope of practice must be defined in a supervisory agreement, which must include "written prescribing instructions" 21 N.C. Admin. Code 32S.0213. NCAC 32S.0213. (".0213 Physician Supervision of Physician Assistants"). See details on prescriptive authority. 21 NCAC 32S.0212. ("Prescriptive authority")	Maybe - a PA may perform medical acts, tasks, or functions under the supervision of a physician but scope of practice must be defined in a supervisory agreement, which must include "written prescribing instructions" 21 N.C. Admin. Code 32S.0213. NCAC 32S.0213. (".0213 Physician Supervision of Physician Assistants"). See details on prescriptive authority. 21 NCAC 32S.0212. ("Prescriptive authority")	Maybe - a PA may perform medical acts, tasks, or functions under the supervision of a physician, but scope of practice must be defined in a supervisory agreement 21 N.C. Admin. Code 32S.0213. NCAC 32S.0213. (".0213 Physician Supervision of Physician Assistants").	Maybe - a PA may perform medical acts, tasks, or functions under the supervision of a physician, but scope of practice must be defined in a supervisory agreement 21 N.C. Admin. Code 32S.0213. NCAC 32S.0213. (".0213 Physician Supervision of Physician Assistants").	Maybe - a PA may perform medical acts, tasks, or functions under the supervision of a physician, but scope of practice must be defined in a supervisory agreement 21 N.C. Admin. Code 32S.0213. NCAC 32S.0213. (".0213 Physician Supervision of Physician Assistants").	Maybe - a PA may perform medical acts, tasks, or functions under the supervision of a physician, but scope of practice must be defined in a supervisory agreement 21 N.C. Admin. Code 32S.0213. NCAC 32S.0213. (".0213 Physician Supervision of Physician Assistants").	Maybe - a PA may perform medical acts, tasks, or functions under the supervision of a physician, but scope of practice must be defined in a supervisory agreement 21 N.C. Admin. Code 32S.0213. NCAC 32S.0213. (".0213 Physician Supervision of Physician Assistants").
**Pharmacist**	Likely No - prescribing not included in practice of pharmacy. N.C.G.S.A. 90-85.3A. ("Practice of pharmacy"). N.C.G.S.A 90-18.4 describes limitations on clinical pharmacist practitioners. NOTE: proposed legislation	Likely No - prescribing not included in practice of pharmacy. N.C.G.S.A. 90-85.3A. ("Practice of pharmacy") N.C.G.S.A 90-18.4 describes limitations on clinical pharmacist practitioners. NOTE: proposed legislation	Maybe - a pharmacist who is approved to be a clinical pharmacist practitioner may modify prescribed drug dosages. N.C.G.S.A. 90-18.4. ("Limitations on clinical pharmacist practitioners")	Likely No - not included in practice of pharmacy N.C.G.S.A. 90-85.3A. ("Practice of pharmacy") and scope of collaborative practice authority is limited to drug therapy agreements. N.C.G.S.A 90-18.4 describes limitations on clinical pharmacist practitioners. NOTE: proposed legislation	Likely No - not included in practice of pharmacy N.C.G.S.A. 90-85.3A. ("Practice of pharmacy") and scope of collaborative practice authority is limited to drug therapy agreements. N.C.G.S.A 90-18.4 describes limitations on clinical pharmacist practitioners. NOTE: proposed legislation	Likely No - not included in practice of pharmacy N.C.G.S.A. 90-85.3A. ("Practice of pharmacy") and scope of collaborative practice authority is limited to drug therapy agreements. N.C.G.S.A 90-18.4 describes limitations on clinical pharmacist practitioners. NOTE: proposed legislation	Likely No - not included in practice of pharmacy N.C.G.S.A. 90-85.3A. ("Practice of pharmacy") and scope of collaborative practice authority is limited to drug therapy agreements. N.C.G.S.A 90-18.4 describes limitations on clinical pharmacist practitioners. NOTE: proposed legislation	Likely No - not included in practice of pharmacy N.C.G.S.A. 90-85.3A. ("Practice of pharmacy") and scope of collaborative practice authority is limited to drug therapy agreements. N.C.G.S.A 90-18.4 describes limitations on clinical pharmacist practitioners. NOTE: proposed legislation

**Medical Assistant**	Likely No - but physicians can delegate to a qualified person any acts, tasks, and functions that are otherwise permitted by law or established by custom. N.C.G.S.A. 90-18 (c)(13) ("Practicing without license; penalties") [78]	Likely No - but physicians can delegate to a qualified person any acts, tasks, and functions that are otherwise permitted by law or established by custom. N.C.G.S.A. 90-18 (c)(13) ("Practicing without license; penalties") [78]	Likely No - but physicians can delegate to a qualified person any acts, tasks, and functions that are otherwise permitted by law or established by custom. N.C.G.S.A. 90-18 (c)(13) ("Practicing without license; penalties") [78]	Maybe - RN and LPN may delegate nursing care activities to unlicensed personnel. 21 NCAC 36.0401. (".0401 Roles of Unlicensed Personnel"). Physicians can delegate to a qualified person any acts, tasks, and functions that are otherwise permitted by law or established by custom. N.C.G.S.A. 90-18 (c)(13) ("Practicing without license; penalties") [78]	Unclear - RN and LPN may delegate nursing care activities to unlicensed personnel. 21 NCAC 36.0401. (".0401 Roles of Unlicensed Personnel"). Physicians can delegate to a qualified person any acts, tasks, and functions that are otherwise permitted by law or established by custom. N.C.G.S.A. 90-18 (c)(13) ("Practicing without license; penalties") [78]	Unclear - RN and LPN may delegate nursing care activities to unlicensed personnel. 21 NCAC 36.0401. (".0401 Roles of Unlicensed Personnel"). Physicians can delegate to a qualified person any acts, tasks, and functions that are otherwise permitted by law or established by custom. N.C.G.S.A. 90-18 (c)(13) ("Practicing without license; penalties") [78]	Unclear - RN and LPN may delegate nursing care activities to unlicensed personnel. 21 NCAC 36.0401. (".0401 Roles of Unlicensed Personnel"). Physicians can delegate to a qualified person any acts, tasks, and functions that are otherwise permitted by law or established by custom. N.C.G.S.A. 90-18 (c)(13) ("Practicing without license; penalties") [78]	Unclear - RN and LPN may delegate nursing care activities to unlicensed personnel. 21 NCAC 36.0401. (".0401 Roles of Unlicensed Personnel"). Physicians can delegate to a qualified person any acts, tasks, and functions that are otherwise permitted by law or established by custom. N.C.G.S.A. 90-18 (c)(13) ("Practicing without license; penalties") [78]
**Emergency Medical Technician**	Likely no - NC Medical Board determines scope of practice for EMS personnel. N.C.G.S.A. 143-514. ("Scope of practice for credentialed emergency medical services personnel") and the list includes diuretics for paramedics, but not for EMTs	Likely no - NC Medical Board determines scope of practice for EMS personnel. N.C.G.S.A. 143-514. ("Scope of practice for credentialed emergency medical services personnel") and the list includes diuretics for paramedics, but not for EMTs	No - NC Medical Board determines scope of practice for EMS personnel. N.C.G.S.A. 143-514. ("Scope of practice for credentialed emergency medical services personnel") and the list does not include warfarin	No - NC Medical Board determines scope of practice for EMS personnel. N.C.G.S.A. 143-514. ("Scope of practice for credentialed emergency medical services personnel"). thrombolytics not listed as approved medication for EMT or AEMT. ("North Carolina Medical Board Approved Medications for Credentialed EMS Personnel") [79]	Likely No - NC Medical Board determines scope of practice for EMS personnel. N.C.G.S.A. 143-514. ("Scope of practice for credentialed emergency medical services personnel"). Ultrasound is not listed . [80]	Likely No - NC Medical Board determines scope of practice for EMS personnel. N.C.G.S.A. 143-514. ("Scope of practice for credentialed emergency medical services personnel"). ABI is not listed . [80]	Maybe - NC Medical Board determines scope of practice for EMS personnel. N.C.G.S.A. 143-514. ("Scope of practice for credentialed emergency medical services personnel"). 12-lead ECG acquisition and interpretation allowed for Paramedic, EMR, and EMTs. 15-lead acquisition only allowed for paramedic. ("North Carolina Medical Board Approved Medications for Credentialed EMS Personnel") [79]	Yes - orthostatic blood pressure is listed in "2021 Procedures Combined" document under "EMS Procedures" heading. . [80]
**Paramedic**	Unclear - NC Medical Board determines scope of practice for EMS personnel. N.C.G.S.A. 143-514. ("Scope of practice for credentialed emergency medical services personnel") and list includes diuretics, but authority may be limited to emergency administration (not prescribing)	Unclear - NC Medical Board determines scope of practice for EMS personnel. N.C.G.S.A. 143-514. ("Scope of practice for credentialed emergency medical services personnel") and list includes diuretics and ace inhibitors, but authority may be limited to emergency administration (not prescribing)	Likely no - NC Medical Board determines scope of practice for EMS personnel. N.C.G.S.A. 143-514. ("Scope of practice for credentialed emergency medical services personnel") but list does not include warfarin, and authority may be limited to emergency administration (not prescribing)	Yes - NC Medical Board determines scope of practice for EMS personnel. N.C.G.S.A. 143-514. ("Scope of practice for credentialed emergency medical services personnel"). Thrombolytics listed as approved medication for paramedic. ("North Carolina Medical Board Approved Medications for Credentialed EMS Personnel") [79]	Likely No - NC Medical Board determines scope of practice for EMS personnel. N.C.G.S.A. 143-514. ("Scope of practice for credentialed emergency medical services personnel"). Ultrasound is not listed. [80]	Likely No - NC Medical Board determines scope of practice for EMS personnel. N.C.G.S.A. 143-514. ("Scope of practice for credentialed emergency medical services personnel"). ABI is not listed. [80]	Maybe - NC Medical Board determines scope of practice for EMS personnel. N.C.G.S.A. 143-514. ("Scope of practice for credentialed emergency medical services personnel"). 12-lead ECG acquisition and interpretation allowed for Paramedic, EMR, and EMTs. 15-lead acquisition only allowed for paramedic. ("North Carolina Medical Board Approved Medications for Credentialed EMS Personnel") [79]	Yes - orthostatic blood pressure is listed in "2021 Procedures Combined" document under "EMS Procedures" heading. [80]
**Community Heath Worker**	No - no licensed scope of practice for community health workers in NC	No - no licensed scope of practice for community health workers in NC	No - no licensed scope of practice for community health workers in NC	No - no licensed scope of practice for community health workers in NC	No - no licensed scope of practice for community health workers in NC	No - no licensed scope of practice for community health workers in NC	No - no licensed scope of practice for community health workers in NC	No - no licensed scope of practice for community health workers in NC

Coding Framework = Yes, Likely Yes, Maybe, Unclear, Likely No, No

Oregon	Prescribing antihypertensives	Prescribing heart failure medicines	Monitoring INR and modifying warfarin Rx	Administering thrombolytics for STEMI	Conducting point of care ultrasound screening for heart disease	Conducting Ankle Brachial Index Test	Conducting ECG	Conducting blood pressure test
**Nurse Practitioner**	Maybe - APRN scope incluides "Prescribing, dispensing, and administration of medications, therapeutic devices and measures." Or. Admin. R. 851-055-0010 ("Scope and Standards of Practice for All Licensed Advanced Practice Registered Nurses"). Must complete 45 hours of advanced pharmacology concepts to obtain prescriptive privilege. Oregon Administrative Rule 851-053-0010. ("Prescriptive Privilege and Dispensing Authority for APRNs")	Maybe - APRN scope incluides "Prescribing, dispensing, and administration of medications, therapeutic devices and measures." Or. Admin. R. 851-055-0010 ("Scope and Standards of Practice for All Licensed Advanced Practice Registered Nurses"). Must complete 45 hours of advanced pharmacology concepts to obtain prescriptive privilege. Oregon Administrative Rule 851-053-0010. ("Prescriptive Privilege and Dispensing Authority for APRNs")	Maybe - APRN scope incluides "Prescribing, dispensing, and administration of medications, therapeutic devices and measures." Or. Admin. R. 851-055-0010 ("Scope and Standards of Practice for All Licensed Advanced Practice Registered Nurses"). Must complete 45 hours of advanced pharmacology concepts to obtain prescriptive privilege. Oregon Administrative Rule 851-053-0010. ("Prescriptive Privilege and Dispensing Authority for APRNs")	Likely Yes - APRN scope incluides "Prescribing, dispensing, and administration of medications, therapeutic devices and measures." Or. Admin. R. 851-055-0010 ("Scope and Standards of Practice for All Licensed Advanced Practice Registered Nurses"). Must complete 45 hours of advanced pharmacology concepts to obtain prescriptive privilege. Oregon Administrative Rule 851-053-0010. ("Prescriptive Privilege and Dispensing Authority for APRNs")	Likely Yes - APRN indpendently provides healthcare services and manages health care. Oregon Administrative Rule 851-055-0010. ("Scope and Standards of Practice for All Licensed Advanced Practice Registered Nurses").	Likely Yes - APRN indpendently provides healthcare services and manages health care. Oregon Administrative Rule 851-055-0010. ("Scope and Standards of Practice for All Licensed Advanced Practice Registered Nurses").	Likely Yes - APRN indpendently provides healthcare services and manages health care. Oregon Administrative Rule 851-055-0010. ("Scope and Standards of Practice for All Licensed Advanced Practice Registered Nurses").	Likely Yes - APRN indpendently provides healthcare services and manages health care. Oregon Administrative Rule 851-055-0010. ("Scope and Standards of Practice for All Licensed Advanced Practice Registered Nurses").
**Registered Nurse**	Likely No - scope of practice for RNs does not include independent prescribing Oregon Administrative Rule 851-045-0060 ("Scope of Practice Standards for Registered Nurses")	Likely No - scope of practice for RNs does not include independent prescribing Oregon Administrative Rule 851-045-0060 ("Scope of Practice Standards for Registered Nurses")	Likely No - scope of practice for RNs does not include independent prescribing Oregon Administrative Rule 851-045-0060 ("Scope of Practice Standards for Registered Nurses")	Unclear - "The licensee may accept and implement orders from a licensed independent practitioner authorized by Oregon statute to independently diagnose and treat." Oregon Administrative Rule 851-045-0040 ("Scope of Practice Standards for all Licensed Nurses"), Oregon Administrative Rule 851-045-0060 ("Scope of Practice Standards for Registered Nurses")	Unclear - "Collecting data from observations, examinations, interviews, and records..." Oregon Administrative Rule 851-045-0060 ("Scope of Practice Standards for Registered Nurses")	Unclear - "Collecting data from observations, examinations, interviews, and records..." Oregon Administrative Rule 851-045-0060 ("Scope of Practice Standards for Registered Nurses")	Unclear - "Collecting data from observations, examinations, interviews, and records..." Oregon Administrative Rule 851-045-0060 ("Scope of Practice Standards for Registered Nurses")	Likely Yes - "Collecting data from observations, examinations, interviews, and records..." Oregon Administrative Rule 851-045-0060 ("Scope of Practice Standards for Registered Nurses")
**Licensed Practical Nurse**	Likely No - LPN scope of practice described at 851-045-0050, which does not list prescribing medications ("Scope of Practice Standards for Licensed Practical Nurses" Or. Admin. R. 851-045-0050	Likely No - LPN scope of practice described at 851-045-0050, which does not list prescribing medications ("Scope of Practice Standards for Licensed Practical Nurses" Or. Admin. R. 851-045-0050	Likely No - LPN scope of practice described at 851-045-0050, which does not list prescribing medications ("Scope of Practice Standards for Licensed Practical Nurses" Or. Admin. R. 851-045-0051	Unclear - "The licensee may accept and implement orders from a licensed independent practitioner authorized by Oregon statute to independently diagnose and treat." Oregon Administrative Rule 851-045-0040 ("Scope of Practice Standards for all Licensed Nurses"), Oregon Administrative Rule 851-045-0050 ("Scope of Practice Standards for Licensed Practical Nurses")	Unclear - "Collecting data from observations, examinations, interviews, and records..." Oregon Administrative Rule 851-045-0050 ("Scope of Practice Standards for Registered Nurses")	Unclear - "Collecting data from observations, examinations, interviews, and records..." Oregon Administrative Rule 851-045-0050 ("Scope of Practice Standards for Registered Nurses")	Unclear - "Collecting data from observations, examinations, interviews, and records..." Oregon Administrative Rule 851-045-0050 ("Scope of Practice Standards for Registered Nurses")	Likely Yes - "Collecting data from observations, examinations, interviews, and records..." Oregon Administrative Rule 851-045-0050 ("Scope of Practice Standards for Registered Nurses")
**Physician Assistant**	Maybe - "PA may issue written or electronic prescriptions … based on PA education, training, experience, and commensurate with collaboration agreement." Oregon Administrative Rule 847-050-0041. ("Prescribing and dispensing privileges")	Maybe - "PA may issue written or electronic prescriptions … based on PA education, training, experience, and commensurate with collaboration agreement." Oregon Administrative Rule 847-050-0041. ("Prescribing and dispensing privileges")	Maybe - "PA may issue written or electronic prescriptions … based on PA education, training, experience, and commensurate with collaboration agreement." Oregon Administrative Rule 847-050-0041. ("Prescribing and dispensing privileges")	Maybe - PA may practice medicine commensorate with their collaborative practice agreement. Oregon Administrative Rule 847-050-0080. ("Collaborative Practice Model")	Maybe - PA may practice medicine commensorate with their collaborative practice agreement. Oregon Administrative Rule 847-050-0080. ("Collaborative Practice Model")	Maybe - PA may practice medicine commensorate with their collaborative practice agreement. Oregon Administrative Rule 847-050-0080. ("Collaborative Practice Model")	Maybe - PA may practice medicine commensorate with their collaborative practice agreement. Oregon Administrative Rule 847-050-0080. ("Collaborative Practice Model")	Maybe - PA may practice medicine commensorate with their collaborative practice agreement. Oregon Administrative Rule 847-050-0080. ("Collaborative Practice Model")
**Pharmacist**	Maybe- not included in list of drugs pharmacists may prescribe. OAR 855-115-0345 ("Services: Prescribing - Protocol Compendium"). Maybe, if included in Collaborative Drug Therapy Management agreement. OAR 855-115-0315. ("Collaborative Drug Therapy Management")	Maybe- not included in list of drugs pharmacists may prescribe. OAR 855-115-0345 ("Services: Prescribing - Protocol Compendium"). Maybe, if included in Collaborative Drug Therapy Management agreement. OAR 855-115-0315. ("Collaborative Drug Therapy Management")	Maybe- not included in list of drugs pharmacists may prescribe. OAR 855-115-0345 ("Services: Prescribing - Protocol Compendium"). Maybe, if included in Collaborative Drug Therapy Management agreement. OAR 855-115-0315. ("Collaborative Drug Therapy Management")	Maybe if included in CDTM "A detailed description of the methods, procedures, decision criteria, and plan…" OAR 855-115-0315. ("Collaborative Drug Therapy Management").	Maybe if included in CDTM "A detailed description of the methods, procedures, decision criteria, and plan…" OAR 855-115-0315. ("Collaborative Drug Therapy Management").	Maybe if included in CDTM "A detailed description of the methods, procedures, decision criteria, and plan…" OAR 855-115-0315. ("Collaborative Drug Therapy Management").	Maybe if included in CDTM "A detailed description of the methods, procedures, decision criteria, and plan…" OAR 855-115-0315. ("Collaborative Drug Therapy Management").	Maybe if included in CDTM "A detailed description of the methods, procedures, decision criteria, and plan…" OAR 855-115-0315. ("Collaborative Drug Therapy Management").

**Medical Assistant**	Likely No - MAs are not licensed in Oregon and prescribe or administer drug defined as practice of medicine. Oregon Revised Statute 677.085. ("Activities constituting practice of medicine").	Likely No - MAs are not licensed in Oregon and prescribe or administer drug defined as practice of medicine. Oregon Revised Statute 677.085. ("Activities constituting practice of medicine").	Likely No - MAs are not licensed in Oregon and prescribe or administer drug defined as practice of medicine. Oregon Revised Statute 677.085. ("Activities constituting practice of medicine").	Unclear -- statement of philosophy from Oregon.gov states "medical assistants should not provide assessments, interpretations, or diagnoses and should not perform invasive procedures" official. ("Use of Unlicensed Healthcare Personnel") [81]	Unclear -- statement of philosophy from Oregon.gov states "medical assistants should not provide assessments, interpretations, or diagnoses and should not perform invasive procedures" official. ("Use of Unlicensed Healthcare Personnel") [81]	Unclear -- statement of philosophy from Oregon.gov states "medical assistants should not provide assessments, interpretations, or diagnoses and should not perform invasive procedures" official. ("Use of Unlicensed Healthcare Personnel") [81]	Unclearo -- statement of philosophy from Oregon.gov states "medical assistants should not provide assessments, interpretations, or diagnoses and should not perform invasive procedures" official. ("Use of Unlicensed Healthcare Personnel") [81]	Unclear -- statement of philosophy from Oregon.gov is only guidance identified [81]
**Emergency Medical Technician**	No - Medication not listed in scope of practice. OAR 847-035-0030. ("Scope of Practice")	No - Medication not listed in scope of practice. OAR 847-035-0030. ("Scope of Practice")	No - Medication not listed in scope of practice. OAR 847-035-0030. ("Scope of Practice")	No - Not listed under drugs EMTS can administer. OAR 847-035-0050. ("Scope of Pratice")	Maybe - "utilize noninvasive diagnostic devices in accordance with manufacturer's recommendation" OAR 847-035-0050. ("Scope of Practice")	Likely no. OAR 847-035-0050. ("Scope of Practice")	Maybe - an EMT-Intermediate may "perform electrocardiographic rhythm interpretation" (see subsection 11 e) OAR 847-035-0050. ("Scope of Practice")	Yes - Take and record vital signs OAR 847-035-0050. ("Scope of Practice")
**Paramedic**	No - Medication not listed in scope of practice. OAR 847-035-0030. ("Scope of Practice")	No - Medication not listed in scope of practice. OAR 847-035-0030. ("Scope of Practice")	No - Medication not listed in scope of practice. OAR 847-035-0030. ("Scope of Practice")	Maybe - if falls under specific written protocols authorized by supervising physician or under direct orders from a licensed physician ORS (See subsection 12 L) OAR 847-035-0050. "Scope of Practice").	Maybe - "utilize noninvasive diagnostic devices in accordance with manufacturer's recommendation" OAR 847-035-0050. ("Scope of Practice")	Likely no. OAR 847-035-0050. ("Scope of Practice")	Yes - a paramedic may "perform electrocardiographic rhythm interpretation" (see subsection 11 e) OAR 847-035-0050. ("Scope of Practice")	Yes - Take and record vital signs OAR 847-035-0050. ("Scope of Practice")
**Community Heath Worker**	No - CHWs do not have a licensed scope of practice in OR. Oregon Revised Statute 677.085. ("Activities constituting practice of medicine").	No - CHWs do not have a licensed scope of practice in OR. Oregon Revised Statute 677.085. ("Activities constituting practice of medicine").	No - CHWs do not have a licensed scope of practice in OR. Oregon Revised Statute 677.085. ("Activities constituting practice of medicine").	No - CHWs do not have a licensed scope of practice in OR. Oregon Revised Statute 677.085. ("Activities constituting practice of medicine").	No - CHWs do not have a licensed scope of practice in OR. Oregon Revised Statute 677.085. ("Activities constituting practice of medicine").	No - CHWs do not have a licensed scope of practice in OR. Oregon Revised Statute 677.085. ("Activities constituting practice of medicine").	No - CHWs do not have a licensed scope of practice in OR. Oregon Revised Statute 677.085. ("Activities constituting practice of medicine").	No - CHWs do not have a licensed scope of practice in OR. Oregon Revised Statute 677.085. ("Activities constituting practice of medicine").

Coding Framework = Yes, Likely Yes, Maybe, Unclear, Likely No, No

Virginia	Prescribing antihypertensives	Prescribing heart failure medicines	Monitoring INR and modifying warfarin Rx	Administering thrombolytics for STEMI	Conducting point of care ultrasound screening for heart disease	Conducting Ankle Brachial Index Test	Conducting ECG	Conducting blood pressure test
**Nurse Practitioner**	Maybe - With appropriate collaboration and consultation with a physician if the NP has less than 5 years experience. Virginia Code - 54.1 - 2957 ("Licensure and practice of advanced practice registered nurses") An NP with at least 5 years of experience can practice without a practice agreement ("Licensure and practice of advanced practice registered nurses, subsection I") A NP with prescriptive authority must obtain 8 hours of CE in pharmacology or pharmacotherapeutics for each biennium. 18VAC90-30-105 ("Continuing competency requirements")	Maybe - With appropriate collaboration and consultation with a physician if the NP has less than 5 years experience. Virginia Code - 54.1 - 2957 ("Licensure and practice of advanced practice registered nurses") An NP with at least 5 years of experience can practice without a practice agreement ("Licensure and practice of advanced practice registered nurses, subsection I") A NP with prescriptive authority must obtain 8 hours of CE in pharmacology or pharmacotherapeutics for each biennium. 18VAC90-30-105 ("Continuing competency requirements")	Maybe - With appropriate collaboration and consultation with a physician if the NP has less than 5 years experience. Virginia Code - 54.1 - 2957 ("Licensure and practice of advanced practice registered nurses") An NP with at least 5 years of experience can practice without a practice agreement ("Licensure and practice of advanced practice registered nurses, subsection I") A NP with prescriptive authority must obtain 8 hours of CE in pharmacology or pharmacotherapeutics for each biennium. 18VAC90-30-105 ("Continuing competency requirements")	Maybe - Virginia Code - 54.1 - 2957 With appropriate collaboration and consultation with a physician if the NP has less than 5 years experience ("Licensure and practice of advanced practice registered nurses") An NP with at least 5 years of experience can practice without a practice agreement ("Licensure and practice of advanced practice registered nurses, subsection I")	Maybe - Virginia Code - 54.1 - 2957 With appropriate collaboration and consultation with a physician if the NP has less than 5 years experience ("Licensure and practice of advanced practice registered nurses") An NP with at least 5 years of experience can practice without a practice agreement ("Licensure and practice of advanced practice registered nurses, subsection I")	Maybe - Virginia Code - 54.1 - 2957 With appropriate collaboration and consultation with a physician if the NP has less than 5 years experience ("Licensure and practice of advanced practice registered nurses") An NP with at least 5 years of experience can practice without a practice agreement ("Licensure and practice of advanced practice registered nurses, subsection I")	Maybe - Virginia Code - 54.1 - 2957 With appropriate collaboration and consultation with a physician if the NP has less than 5 years experience ("Licensure and practice of advanced practice registered nurses") An NP with at least 5 years of experience can practice without a practice agreement ("Licensure and practice of advanced practice registered nurses, subsection I")	Likely Yes - Virginia Code - 54.1 - 2957 With appropriate collaboration and consultation with a physician if the NP has less than 5 years experience ("Licensure and practice of advanced practice registered nurses") An NP with at least 5 years of experience can practice without a practice agreement ("Licensure and practice of advanced practice registered nurses, subsection I")
**Registered Nurse**	Likely No - prescribing not listed within scope of practice of RN. See Virginia Code 54.1-3000 ("Definitions") and A prescription for a controlled substance may be issued only by a practitioner of medicine, osteopathy … licensed ARNP, licensed PA … Virginia Code 54.1-3303 ("Prescriptions to be issued and drugs to be dispended for medical or therapeutic purposes only")	Likely No - prescribing not listed within scope of practice of RN. See Virginia Code 54.1-3000 ("Definitions") and A prescription for a controlled substance may be issued only by a practitioner of medicine, osteopathy … licensed ARNP, licensed PA … Virginia Code 54.1-3303 ("Prescriptions to be issued and drugs to be dispended for medical or therapeutic purposes only")	Likely No - prescribing not listed within scope of practice of RN. See Virginia Code 54.1-3000 ("Definitions") and A prescription for a controlled substance may be issued only by a practitioner of medicine, osteopathy … licensed ARNP, licensed PA … Virginia Code 54.1-3303 ("Prescriptions to be issued and drugs to be dispended for medical or therapeutic purposes only")	Likely Yes - The definition of registered nurse includes, "the administration of medications and treatments as prescribed by any person authorized by law to prescribe such medications and treatments." Virginia Code - 54.1 3000. ("Definitions")	Unclear - Describes registered nursing practice as observation, care and counsel of groups… prevention of illness or disease. Virginia Code 54.1-3000 ("Definitions"),see also Virginia Board of Nursing Guidance Document 90-6 [82]	Unclear - Describes registered nursing practice as observation, care and counsel of groups… prevention of illness or disease. Virginia Code 54.1-3000 ("Definitions"),see also Virginia Board of Nursing Guidance Document 90-6 [82]	Unclear - Describes registered nursing practice as observation, care and counsel of groups… prevention of illness or disease. Virginia Code 54.1-3000 ("Definitions"),see also Virginia Board of Nursing Guidance Document 90-6 [82]	Unclear - Describes registered nursing practice as observation, care and counsel of groups… prevention of illness or disease. Virginia Code 54.1-3000 ("Definitions"),see also Virginia Board of Nursing Guidance Document 90-6 [82]
**Licensed Practical Nurse**	Likely No - prescribing not listed within scope of practice of LPNs. See Virginia Code 54.1-3000 ("Definitions") and A prescription for a controlled substance may be issued only by a practitioner of medicine, osteopathy … licensed ARNP, licensed PA … Virginia Code 54.1-3303 ("Prescriptions to be issued and drugs to be dispended for medical or therapeutic purposes only")	Likely No - prescribing not listed within scope of practice of LPNs. See Virginia Code 54.1-3000 ("Definitions") and A prescription for a controlled substance may be issued only by a practitioner of medicine, osteopathy … licensed ARNP, licensed PA … Virginia Code 54.1-3303 ("Prescriptions to be issued and drugs to be dispended for medical or therapeutic purposes only")	Likely No - prescribing not listed within scope of practice of LPNs. See Virginia Code 54.1-3000 ("Definitions") and A prescription for a controlled substance may be issued only by a practitioner of medicine, osteopathy … licensed ARNP, licensed PA … Virginia Code 54.1-3303 ("Prescriptions to be issued and drugs to be dispended for medical or therapeutic purposes only")	Unclear - the definition of LPN includes, "selected nursing acts in the care of individuals or groups … maintenance of health, prevention of illness or disease" Virginia Code 54.1-3000. ("Definitions"). See also, Virginia Board of Nursing Guidance Document 90-6 [82]	Unclear - the definition of LPN includes, "selected nursing acts in the care of individuals or groups … maintenance of health, prevention of illness or disease" Virginia Code 54.1-3000. ("Definitions"). See also, Virginia Board of Nursing Guidance Document 90-6 [82]	Unclear - the definition of LPN includes, "selected nursing acts in the care of individuals or groups … maintenance of health, prevention of illness or disease" Virginia Code 54.1-3000. ("Definitions"). See also, Virginia Board of Nursing Guidance Document 90-6 [82]	Unclear - the definition of LPN includes, "selected nursing acts in the care of individuals or groups … maintenance of health, prevention of illness or disease" Virginia Code 54.1-3000. ("Definitions"). See also, Virginia Board of Nursing Guidance Document 90-6 [82]	Unclear - the definition of LPN includes, "selected nursing acts in the care of individuals or groups … maintenance of health, prevention of illness or disease" Virginia Code 54.1-3000. ("Definitions"). See also, Virginia Board of Nursing Guidance Document 90-6 [82]
**Physician Assistant**	Maybe - Scope determined by practice agreement. 18 VAC 85-50-40. ("General requirements"). Perform medical care services that are within the scope of practice and proficiency of the patient care team physicians. 18VAC 85-50-115. ("Responsibilities of the physician assistant"). Must submit evidence of successful completion of minimum 35 hours of acceptable training to the board of pharmacology for prescriptive authority. 18VAC85-50-130	Maybe - Scope determined by practice agreement. 18 VAC 85-50-40. ("General requirements"). Perform medical care services that are within the scope of practice and proficiency of the patient care team physicians. 18VAC 85-50-115. ("Responsibilities of the physician assistant"). Must submit evidence of successful completion of minimum 35 hours of acceptable training to the board of pharmacology for prescriptive authority. 18VAC85-50-131	Maybe - Scope determined by practice agreement. 18 VAC 85-50-40. ("General requirements"). Perform medical care services that are within the scope of practice and proficiency of the patient care team physicians. 18VAC 85-50-115. ("Responsibilities of the physician assistant"). Must submit evidence of successful completion of minimum 35 hours of acceptable training to the board of pharmacology for prescriptive authority. 18VAC85-50-132	Maybe - depends on scope in practice agreement. 18 VAC 85-50-40. ("General requirements"). Perform medical care services that are within the scope of practice and proficiency of the patient care team physicians. 18VAC 85-50-115. ("Responsibilities of the physician assistant")	Maybe - depends on scope in practice agreement. 18 VAC 85-50-40. ("General requirements"). Perform medical care services that are within the scope of practice and proficiency of the patient care team physicians. 18VAC 85-50-115. ("Responsibilities of the physician assistant")	Maybe - depends on scope in practice agreement. 18 VAC 85-50-40. ("General requirements"). Perform medical care services that are within the scope of practice and proficiency of the patient care team physicians. 18VAC 85-50-115. ("Responsibilities of the physician assistant")	Maybe - depends on scope in practice agreement. 18 VAC 85-50-40. ("General requirements"). Perform medical care services that are within the scope of practice and proficiency of the patient care team physicians. 18VAC 85-50-115. ("Responsibilities of the physician assistant")	Maybe - depends on scope in practice agreement. 18 VAC 85-50-40. ("General requirements"). Perform medical care services that are within the scope of practice and proficiency of the patient care team physicians. 18VAC 85-50-115. ("Responsibilities of the physician assistant")

**Pharmacist**	Maybe - Collaborative agreements can "authorize cooperative procedures related to treatment using drug therapy, laboratory tests, or medical devices..." Code of Virginia 54.1-3300.1. ("Participation in collaborative agreements; regulations to be promulgated by the Boards of Medicine and Pharmacy").	Maybe - Collaborative agreements can "authorize cooperative procedures related to treatment using drug therapy, laboratory tests, or medical devices..." Code of Virginia 54.1-3300.1. ("Participation in collaborative agreements; regulations to be promulgated by the Boards of Medicine and Pharmacy").	Maybe - Collaborative agreements can "authorize cooperative procedures related to treatment using drug therapy, laboratory tests, or medical devices..." Code of Virginia 54.1-3300.1. ("Participation in collaborative agreements; regulations to be promulgated by the Boards of Medicine and Pharmacy").	Maybe - Collaborative agreements can "authorize cooperative procedures related to treatment using drug therapy, laboratory tests, or medical devices..." Code of Virginia 54.1-3300.1. ("Participation in collaborative agreements; regulations to be promulgated by the Boards of Medicine and Pharmacy").	Maybe - Collaborative agreements can "authorize cooperative procedures related to treatment using drug therapy, laboratory tests, or medical devices..." Code of Virginia 54.1-3300.1. ("Participation in collaborative agreements; regulations to be promulgated by the Boards of Medicine and Pharmacy").	Maybe - Collaborative agreements can "authorize cooperative procedures related to treatment using drug therapy, laboratory tests, or medical devices..." Code of Virginia 54.1-3300.1. ("Participation in collaborative agreements; regulations to be promulgated by the Boards of Medicine and Pharmacy").	Maybe - Collaborative agreements can "authorize cooperative procedures related to treatment using drug therapy, laboratory tests, or medical devices..." Code of Virginia 54.1-3300.1. ("Participation in collaborative agreements; regulations to be promulgated by the Boards of Medicine and Pharmacy").	Maybe - Collaborative agreements can "authorize cooperative procedures related to treatment using drug therapy, laboratory tests, or medical devices..." Code of Virginia 54.1-3300.1. ("Participation in collaborative agreements; regulations to be promulgated by the Boards of Medicine and Pharmacy").
**Medical Assistant**	No - MAs are not licensed in Virginia and a prescription for a controlled substance may be issued only by a practitioner of medicine, osteopathy ... licensed ARNP, licensed PA ... Virginia Code 54.1-3303 ("Prescriptions to be issued and drugs to be dispended for medical or therapeutic purposes only")	No - MAs are not licensed in Virginia and a prescription for a controlled substance may be issued only by a practitioner of medicine, osteopathy ... licensed ARNP, licensed PA ... Virginia Code 54.1-3303 ("Prescriptions to be issued and drugs to be dispended for medical or therapeutic purposes only")	No - MAs are not licensed in Virginia and a prescription for a controlled substance may be issued only by a practitioner of medicine, osteopathy ... licensed ARNP, licensed PA ... Virginia Code 54.1-3303 ("Prescriptions to be issued and drugs to be dispended for medical or therapeutic purposes only")	Likely No - Prescriber may authorize the administration of controlled substances by personnel who have been properly trained to assist a doctor ... provided the method does not include intravenous, intrathecal, or epidural administration... Code of Virginia 54.1-3408 ("Professional use by practitioners - subsection U")	Unclear - MAs are not licensed in VA, but nurses can delegate tasks to unlicensed personnel. 12VAC90-19-250 ("Criteria for delegation")	Unclear - MAs are not licensed in VA, but nurses can delegate tasks to unlicensed personnel. 12VAC90-19-250 ("Criteria for delegation")	Unclear - MAs are not licensed in VA, but nurses can delegate tasks to unlicensed personnel. 12VAC90-19-250 ("Criteria for delegation")	Unclear - MAs are not licensed in VA, but nurses can delegate tasks to unlicensed personnel. 12VAC90-19-250 ("Criteria for delegation")
**Emergency Medical Technician**	No - A prescription for a controlled substance may be issued only by a practitioner of medicine, osteopathy ... licensed ARNP, licensed PA ... Virginia Code 54.1-3303 ("Prescriptions to be issued and drugs to be dispended for medical or therapeutic purposes only")	No - A prescription for a controlled substance may be issued only by a practitioner of medicine, osteopathy ... licensed ARNP, licensed PA ... Virginia Code 54.1-3303 ("Prescriptions to be issued and drugs to be dispended for medical or therapeutic purposes only")	No - A prescription for a controlled substance may be issued only by a practitioner of medicine, osteopathy ... licensed ARNP, licensed PA ... Virginia Code 54.1-3303 ("Prescriptions to be issued and drugs to be dispended for medical or therapeutic purposes only")	No - Scope of practice for EMS personnel authorized in EMS Procedures and Medications Schedule. 12 VAC 5-31-1050. ("Scope of Practice"). Scope of Practice Procedures and Formulary [83]	No - Scope of practice for EMS personnel authorized in EMS Procedures and Medications Schedule. 12 VAC 5-31-1050. ("Scope of Practice"). Scope of Practice Procedures and Formulary [83]	Likely no. Scope of practice for EMS personnel authorized in EMS Procedures and Medications Schedule. 12 VAC 5-31-1050. ("Scope of Practice") Scope of Practice Procedures and Formulary [83]	Maybe - Scope of practice for EMS personnel authorized in EMS Procedures and Medications Schedule. 12 VAC 5-31-1050. ("Scope of Practice"). Acquisition of ECG permitted, and rhythm interpretation permitted. Scope of Practice Procedures and Formulary [83]	Yes - Scope of practice for EMS personnel authorized in EMS Procedures and Medications Schedule. 12 VAC 5-31-1050. ("Scope of Practice"). Scope of Practice Procedures and Formulary [83]
**Paramedic**	No - A prescription for a controlled substance may be issued only by a practitioner of medicine, osteopathy ... licensed ARNP, licensed PA ... Virginia Code 54.1-3303 ("Prescriptions to be issued and drugs to be dispended for medical or therapeutic purposes only")	No - A prescription for a controlled substance may be issued only by a practitioner of medicine, osteopathy ... licensed ARNP, licensed PA ... Virginia Code 54.1-3303 ("Prescriptions to be issued and drugs to be dispended for medical or therapeutic purposes only")	No - A prescription for a controlled substance may be issued only by a practitioner of medicine, osteopathy ... licensed ARNP, licensed PA ... Virginia Code 54.1-3303 ("Prescriptions to be issued and drugs to be dispended for medical or therapeutic purposes only")	Maybe - paramedic can administer thrombolytics with additional training. Scope of practice for EMS personnel authorized in EMS Procedures and Medications Schedule. 12 VAC 5-31-1050. ("Scope of practice"). Documentation of EMS agency (post-affiliation training, specifc to red dot skills and medications -- must have written authorization kept and maintained with the EMS Agency) ("Scope of Practice Procedures and Formulary") [4]	Maybe - Scope of practice for EMS personnel authorized in EMS Procedures and Medications Schedule. 12 VAC 5-31-1050. ("Scope of Practice"). Ultrasonography is listed under "diagnostic procedures" permitted by paramedic with additional training, but not listed under "POC testing." ("Scope of Practice Procedures and Formulary") [83]	Likely no. Scope of practice for EMS personnel authorized in EMS Procedures and Medications Schedule. 12 VAC 5-31-1050. ("Scope of Practice") Scope of Practice Procedures and Formulary [83]	Maybe - Scope of practice for EMS personnel authorized in EMS Procedures and Medications Schedule. 12 VAC 5-31-1050. ("Scope of Practice"). Scope of Practice Procedures and Formulary [83]	Yes - Scope of practice for EMS personnel authorized in EMS Procedures and Medications Schedule. 12 VAC 5-31-1050. ("Scope of Practice"). Scope of Practice Procedures and Formulary [83]
**Community Heath Worker**	No - CHWs do not have a licensed scope of practice. A prescription for a controlled substance may be issued only by a practitioner of medicine, osteopathy ... licensed ARNP, licensed PA ... Virginia Code 54.1-3303 ("Prescriptions to be issued and drugs to be dispended for medical or therapeutic purposes only")	No - CHWs do not have a licensed scope of practice. A prescription for a controlled substance may be issued only by a practitioner of medicine, osteopathy ... licensed ARNP, licensed PA ... Virginia Code 54.1-3303 ("Prescriptions to be issued and drugs to be dispended for medical or therapeutic purposes only")	No - CHWs do not have a licensed scope of practice. A prescription for a controlled substance may be issued only by a practitioner of medicine, osteopathy ... licensed ARNP, licensed PA ... Virginia Code 54.1-3303 ("Prescriptions to be issued and drugs to be dispended for medical or therapeutic purposes only")	No - No legal scope of practice for CHWs in VA. Unlawful to practice without license. Code of Virginia 54.1-2902 ("Unlawful to practice without license")	No - No legal scope of practice for CHWs in VA. Unlawful to practice without license. Code of Virginia 54.1-2902 ("Unlawful to practice without license")	No - No legal scope of practice for CHWs in VA. Unlawful to practice without license. Code of Virginia 54.1-2902 ("Unlawful to practice without license")	No - No legal scope of practice for CHWs in VA. Unlawful to practice without license. Code of Virginia 54.1-2902 ("Unlawful to practice without license")	No - No legal scope of practice for CHWs in VA. Unlawful to practice without license. Code of Virginia 54.1-2902 ("Unlawful to practice without license")

Coding Framework = Yes, Likely Yes, Maybe, Unclear, Likely No, No

WASHINGTON	Prescribe antihypertensives	Prescribe heart failure medicines	Monitor INR and modifying warfarin Rx	Administer thrombolytics for STEMI	Conduct point of care ultrasound screening for heart disease	Conduct Ankle Brachial Index Test	Conduct electrocardiogram (ECG or EKG)	Conduct blood pressure test
**Nurse Practitioner**	Maybe - RCW 18.79.250; WAC 246-840-300; WA State Nursing Commission[84] Education/application requirement. WAC 246-840-410	Maybe - RCW 18.79.250; WAC 246-840-300; WA State Nursing Commission[84] Education/application requirement. WAC 246-840-411	Maybe - RCW 18.79.250; WAC 246-840-300; WA State Nursing Commission[84] Education/application requirement. WAC 246-840-412	Likely yes - an ARNP can prescribe medications and implement treatment plans per WAC 246-840-300	Likely yes - RCW 18.79.250; WAC 246-840-300	Likely yes - an ARNP can "Examine patients and establish diagnoses by patient history, physical examination, and other methods of assessment" per WAC 246-840-300	Likely yes. An ARNP can " Order, collect, perform, and interpret diagnostic tests" per WAC 246-840-300	Likely yes - an ARNP can "Examine patients and establish diagnoses by patient history, physical examination, and other methods of assessment" per WAC 246-840-300
**Registered Nurse**	Likely no - not included in scope of practice. (RCW 18.79.260; WAC 246-840-705)	Likely no - not included in scope of practice. (RCW 18.79.260; WAC 246-840-706)	Likely no - not included in scope of practice. (RCW 18.79.260; WAC 246-840-707)	Yes - a registered nurse can administer medications at or under the general direction of practitioners listed at RCW 18.79.260 (2)	Yes - ("Washington State Board of Nursing - Commonly Asked Questions - Registered Nurse") [84]	Unclear, WAC 246-840-705 permitting a registered nurse to conduct "the assessment, analysis, diagnosis, planning, implementation and evaluation of nursing care"; RCW 18.79.040	Likely yes, WAC 246-840-705 permitting a registered nurse to conduct "the assessment, analysis, diagnosis, planning, implementation and evaluation of nursing care"; RCW 18.79.040. LPN can perform EKG. ("Washington State Board of Nursing. Licensed Practical Nurse | Washington State Board of Nursing") [85]	Likely yes, WAC 246-840-705 permitting a registered nurse to conduct "the assessment, analysis, diagnosis, planning, implementation and evaluation of nursing care"; RCW 18.79.040
**Licensed Practical Nurse**	Likely no. Not included in scope of practice. RCW 18.79.270; WAC 246-840-705. ("Licensed practical nurse--Activities allowed")	Likely no. Not included in scope of practice. RCW 18.79.270; WAC 246-840-705. ("Licensed practical nurse--Activities allowed")	Likely no. Not included in scope of practice. RCW 18.79.270; WAC 246-840-705. ("Licensed practical nurse--Activities allowed")	Maybe, if there is a written order RCW 18.79.270 ("Licensed practical nurse--Activities allowed")	Yes, Washington State Board of Nursing - Commonly Asked Questions - Licensed Practical Nurse [85] RCW 18.79.270 ("Licensed practical nurse--Activities allowed")	Unclear - an LPN may "perform nursing care" RCW 18.79.270 ("Licensed practical nurse--Activities allowed")	Yes, Washington board of nursing comparison of LPN and MA scope of practice states that an LPN can conduct an EKG [86]] RCW 18.79.270 ("Licensed practical nurse--Activities allowed")	Likely yes, an LPN may "perform nursing care" RCW 18.79.270 ("Licensed practical nurse--Activities allowed")
**Physician Assistant**	Maybe - per RCW 18.71A.030 a Physician Assistant has the same prescriptive authority as the sponsoring physician and scope of practice is determined by practice agreement with sponsoring physician	Maybe - per RCW 18.71A.030 a Physician Assistant has the same prescriptive authority as the sponsoring physician and scope of practice is determined by practice agreement with sponsoring physician	Maybe - per RCW 18.71A.030 a Physician Assistant has the same prescriptive authority as the sponsoring physician and scope of practice is determined by practice agreement with sponsoring physician	Maybe - per RCW 18.71A.030 a Physician Assistant has the same prescriptive authority as the sponsoring physician and scope of practice is determined by practice agreement with sponsoring physician	Maybe - per RCW 18.71A.030 a Physician Assistant has the same prescriptive authority as the sponsoring physician and scope of practice is determined by practice agreement with sponsoring physician	Maybe - per RCW 18.71A.030 a Physician Assistant has the same prescriptive authority as the sponsoring physician and scope of practice is determined by practice agreement with sponsoring physician	Maybe - per RCW 18.71A.030 a Physician Assistant has the same prescriptive authority as the sponsoring physician and scope of practice is determined by practice agreement with sponsoring physician	Maybe - per RCW 18.71A.030 a Physician Assistant has the same prescriptive authority as the sponsoring physician and scope of practice is determined by practice agreement with sponsoring physician
**Pharmacist**	Maybe - may initiate, modify or continue drug therapy under collaborative practice agreement with authorized prescriber (RCW 18.64.011) ("Definitions")[86]	Maybe - may initiate, modify or continue drug therapy under collaborative practice agreement with authorized prescriber (RCW 18.64.011) ("Definitions")[86]	Maybe - may initiate, modify or continue drug therapy under collaborative practice agreement with authorized prescriber (RCW 18.64.011) ("Definitions")[86]	Maybe. if included in the terms of the CDTA as part of the procedures to follow when making drug treatment decisions. RCW 18.64.011) ("Definitions") [86]	Maybe. if included in the terms of the CDTA as part of the procedures to follow when making drug treatment decisions. RCW 18.64.011) ("Definitions") [86]	Maybe. if included in the terms of the CDTA as part of the procedures to follow when making drug treatment decisions. RCW 18.64.011) ("Definitions") [86]	Maybe. if included in the terms of the CDTA as part of the procedures to follow when making drug treatment decisions. RCW 18.64.011) ("Definitions") [86]	Maybe. if included in the terms of the CDTA as part of the procedures to follow when making drug treatment decisions. RCW 18.64.011) ("Definitions") [86]
**Medical Assistant**	No. RCW 18.360.050. ("Authorized duties")	No. RCW 18.360.050. ("Authorized duties")	No. RCW 18.360.050. ("Authorized duties")	No - "A medical assistant-certified may establish intravenous lines for diagnostic or therapeutic purposes, without administering medications..." - RCW 18.360.050 ("Authorized duties")	Unclear. "diagnostic testing" is listed per RCW 18.360.050(1)(d)(i), but not specifically ultrasound. RCW 18.360.050. ("Authorized duties")	Unclear - not listed in scope of practice. RCW 18.360.050(1)(b)(3) ("Authorized duties")	Maybe - if "delegated by, and under the supervision of, a heath care practitioner" Electrocardiography explicitly listed.- RCW 18.360.050(1)(d)(i) ("Authorized duties")	Maybe, if "delegated by, and under the supervision of, a heath care practitioner" - RCW 18.360.050(1)(b)(3) and (4)€ ("Authorized duties")
**Emergency Medical Technician**	No. ("Who May Prescribe and Administer Prescriptions in Washington State") [87]	No. ("Who May Prescribe and Administer Prescriptions in Washington State") [87]	No. ("Who May Prescribe and Administer Prescriptions in Washington State") [87]	No. "Approved Procedures and Skills for Certified EMS Providers" [88]	No, ultrasound and imaging not listed in listed in Approved Procedures and Skills for Certified EMS Providers [88]	Likely no - not listed in scope of practice. ("Approved Procedures and Skills for Certified EMS Providers") [88]	Yes. ("Approved Procedures and Skills for Certified EMS Providers") [88]	Yes. ("Approved Procedures and Skills for Certified EMS Providers") [88]
**Paramedic**	No. ("Who May Prescribe and Administer Prescriptions in Washington State") [87]	No. ("Who May Prescribe and Administer Prescriptions in Washington State") [87]	No. ("Who May Prescribe and Administer Prescriptions in Washington State") [87]	Yes. ("Approved Procedures and Skills for Certified EMS Providers") [88]	No, ultrasound and imaging not listed in listed in "Approved Procedures and Skills for Certified EMS Providers" [88]	Likely no - not listed in scope of practice. ("Approved Procedures and Skills for Certified EMS Providers") [88]	Yes. ("Approved Procedures and Skills for Certified EMS Providers") [88]	Yes. ("Approved Procedures and Skills for Certified EMS Providers") [88]
**Community Heath Worker**	No, WA does not have a legal scope of practice for CHWs. ("Who May Prescribe and Administer Prescriptions in Washington State") [87]	No, WA does not have a legal scope of practice for CHWs. ("Who May Prescribe and Administer Prescriptions in Washington State") [87]	No, WA does not have a legal scope of practice for CHWs. ("Who May Prescribe and Administer Prescriptions in Washington State") [87]	No, WA does not have a legal scope of practice for CHWs. ("Who May Prescribe and Administer Prescriptions in Washington State") [87]	No, WA does not have a legal scope of practice for CHWs.	No, WA does not have a legal scope of practice for CHWs.	No, WA does not have a legal scope of practice for CHWs.	No, WA does not have a legal scope of practice for CHWs.

Appendix C.

Table S9. Jurisdiction Alignment Scores for Scope of Practice Laws Effective July 1, 2024

	Prescribing antihyper-tensive medicines	Prescribing heart failure medicines	Monitoring of INR and modifying anticoagulant dosing	Administering thrombo-lytics for STEMI	Conducting ultrasound screening for heart disease	Conducting Ankle Brachial Index Test	Conducting electrocard-iogram	Conducting blood pressure test	Average
NP	0.69	0.69	0.69	0.92	0.85	0.85	0.85	1.00	0.82
RN	1.00	1.00	0.92	0.69	0.92	1.00	0.85	0.85	0.90
LPN	1.00	1.00	1.00	0.46	0.77	0.92	0.77	0.62	0.83
PA	0.92	0.92	0.92	0.92	0.92	0.92	0.92	0.85	0.91
Pharm	0.77	0.77	0.85	0.77	0.77	0.77	0.77	0.77	0.78
MA	1.00	1.00	1.00	0.64	0.91	0.91	0.73	0.64	0.85
EMT	1.00	1.00	1.00	0.91	0.82	1.00	0.45	1.00	0.90
Para	0.91	0.91	1.00	0.55	0.64	1.00	0.82	1.00	0.85
CHW	1.00	1.00	1.00	1.00	1.00	1.00	1.00	0.92	0.99

Note: NP – Nurse Practitioner, RN – Registered Nurse, LPN – Licensed Practical Nurse, PA – Physician Assistant, Pharm – Pharmacist, MA – Medical Assistant, EMT – Emergency Medical Technician, Para – Paramedic, CHW – Community Health Worker.
